# Design, Synthesis, and In Vitro Evaluation of 4-(Arylchalcogenyl)methyl)-1H-1,2,3-triazol-1-yl-menadione: Exploring Their Potential Against *Tuberculosis*

**DOI:** 10.3390/ph18060797

**Published:** 2025-05-26

**Authors:** Nathália L. B. Santos, Luana S. Gomes, Ruan C. B. Ribeiro, Alcione S. de Carvalho, Maria Cristina S. Lourenço, Laís Machado Marins, Sandy Polycarpo Valle, Thiago H. Doring, Adriano D. Andricopulo, Aldo S. de Oliveira, Vitor F. Ferreira, Fernando de C. da Silva, Luana da Silva Magalhães Forezi, Vanessa Nascimento

**Affiliations:** 1 Instituto de Química, Universidade Federal Fluminense, Campus do Valonguinho, Niterói 24020-141, RJ, Brazil; nathalia.l.b.s@gmail.com (N.L.B.S.); luanagomes@id.uff.br (L.S.G.); ruancarlos@id.uff.br (R.C.B.R.); alcionecarvalho@id.uff.br (A.S.d.C.); laismachado@id.uff.br (L.M.M.); sandypolycarpo@id.uff.br (S.P.V.); luanaforezi@id.uff.br (L.d.S.M.F.); 2 Laboratório de Bacteriologia e Bioensaios, Campus Manguinhos—Fiocruz—Fiocruz, Rio de Janeiro 21040-361, RJ, Brazil; cristina.lourenco@ini.fiocruz.br; 3 Departamento de Ciências Exatas e Educação (CEE), Centro Tecnológico, de Ciências Exatas e Educação (CTE), Universidade Federal de Santa Catarina (UFSC), Blumenau 89036-256, SC, Brazil; thiago.doring@ufsc.br; 4 Laboratório de Química Medicinal e Computacional (LQMC), Instituto de São Carlos de Física (IFSC), Universidade de São Paulo (USP), Av. João Dagnone, 1100, São Carlos 13563-120, SP, Brazil; aandrico@ifsc.usp.br; 5 Instituto Gulbenkian Institute de Medicina Molecular (GIMM), Faculdade de Medicina, Universidade de Lisboa, 1649-028 Lisboa, Portugal; aldo.sena@ufsc.br; 6 Departamento de Tecnologia Farmacêutica, Faculdade de Farmácia, Universidade Federal Fluminense, Niterói 24241-000, RJ, Brazil; vitorferreira@id.uff.br

**Keywords:** 1,4-naphthoquinones, selenium, sulfur, CuAAC, *Mycobacterium tuberculosis* H37Rv (ATCC 27294)

## Abstract

**Background/Objectives:** In this study, a novel series of 4-(arylchalcogenyl)methyl)-1H-1,2,3-Triazol-1-yl-menadione derivatives were synthesized to explore their potential as new antituberculosis (anti-TB) agents. Selenium-containing compounds are known for their significant antimycobacterial activity, which motivated their inclusion in the design. **Methods:** The target compounds were synthesized via a copper(I)-catalyzed azide-alkyne cycloaddition (CuAAC) reaction, affording yields ranging from 34% to 93%. All compounds were evaluated in vitro for anti-TB activity against *Mycobacterium tuberculosis* H37Rv (ATCC 27294), as well as a drug-resistant strain (T113/09). **Results:** Several selenium-containing derivatives exhibited promising activity. Compounds **9b** and **9g** were equipotent to the first-line anti-TB drug, and one compound surpassed its activity. Notably, compounds **9a**, **9b**, **9g**, and **9h** also showed efficacy against the INH- and RIF-resistant *Mtb* strain T113/09. **Conclusions:** The efficacy of selenium-containing triazole-menadione hybrids against both sensitive and resistant *Mtb* strains highlight their potential as candidates for addressing antimicrobial resistance in TB treatment. Further investigations are required to understand their mechanisms of action and assess their in vivo therapeutic potential..

## 1. Introduction

Tuberculosis (TB) remains a critical global health issue, ranking as the second leading cause of death from an infectious agent in 2022, only behind COVID-19, and causing nearly twice as many deaths as HIV/AIDS. According to the World Health Organization (WHO), 10.6 million people developed TB in 2022, with 1.3 million deaths reported—167,000 of which were among individuals living with HIV [1]. The disease is caused by *Mycobacterium tuberculosis* (*Mtb*), which is transmitted through airborne particles expelled by infected individuals, especially during coughing. Symptoms such as cough, fever, night sweats, and weight loss often present mildly, leading to delays in diagnosis and treatment. In this context, one infected person can transmit TB to 10–15 others in a year. The disease primarily affects the lungs but can also spread to other organs. Without treatment, TB has a mortality rate of approximately 50% [1,2].

Despite global efforts having saved over 75 million lives since 2000, challenges in diagnosis and treatment persist. In 2022, only about 7.5 million of the estimated 10.6 million TB cases were detected and reported, with 3.1 million cases remaining unaccounted for [3]. Treatment for TB is lengthy and includes a six-month regimen with four first-line drugs, isoniazid, pyrazinamide, ethambutol, and rifampicin, followed by a maintenance phase with isoniazid and rifampicin. Nevertheless, the emergence of multidrug-resistant (MDR-TB) and extensively drug-resistant (XDR-TB) strains of *Mtb* highlights the need for second-line drugs in treatment. These include fluoroquinolones, d-cycloserine, linezolid, bedaquiline, and delamanid. Treating these resistant forms of TB requires longer treatments, lasting up to 28 months. However, there are drawbacks associated with this approach, including a higher likelihood of adverse effects, increased costs due to prolonged treatment, and limitations in effectiveness against latent mycobacterial infections. These factors can potentially hinder the efficacy and safety of the treatment. Therefore, the quest for novel therapeutic agents that offer enhanced efficacy and improved safety in the treatment of TB remains a challenge for scientists [4].

**Figure 1:**
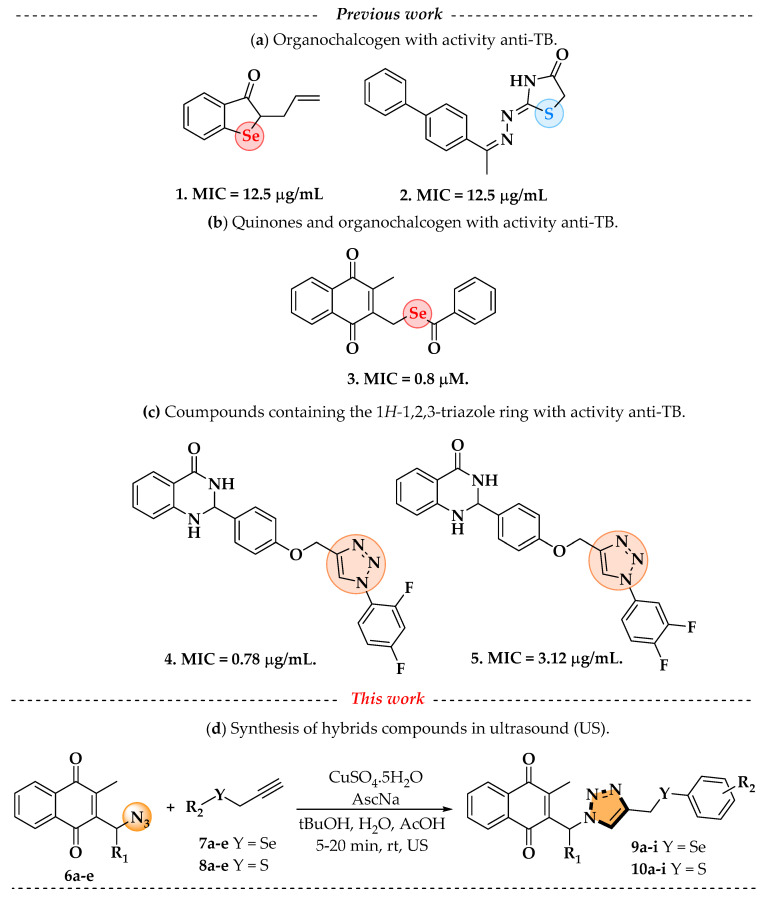
(**a**) Organochalcogens with activity anti-TB; (**b**) quinones–chalcogens with anti-TB activity; (**c**) 1*H*-1,2,3-triazole with their anti-TB activity; (**d**) Compounds proposed in this work in US.

In this context, organochalcogens (organosulfur and organoselenium) have garnered significant interest among synthetic and medicinal chemists in the past decade due to their remarkable biological properties, as well as their versatility as reagents and intermediates in organic synthesis and catalysis [5,6]. Given their wide range of applications, particularly as therapeutic agents, these compounds have shown promising antibacterial capabilities and may be a viable choice for the development of new TB medicines, especially considering the urgent need for novel remedies, owing to medication resistance [7,8]. Notably, compounds containing chalcogens with anti-TB activity have already been described in the literature, such as the work by Thanna et al. [9], with structure **1** presenting a minimum inhibitory concentration (MIC) of 12.5 µg/mL and that of Pasha et al. [10], with structure **2** also showing a MIC of 12.5 µg/mL (Figure 1a). These promising results are associated with the mechanisms by which chalcogen-containing compounds act, such as the inhibition of thiol-dependent enzymes and the modulation of redox homeostasis in *Mtb*, which can impair bacterial antioxidant defenses and metabolic balance [11,12].

In addition, naphthoquinones are a part of the quinone family and are classified as secondary metabolites. They play a crucial role in energy production by acting as electron transport agents. The structure of naphthoquinones consists of various isomers that determine the position of their carbonyl groups, influencing their physical, chemical, and biological properties. Therefore, these compounds have a wide spectrum of activities, including antiviral [13], antibacterial [14], antineoplastic [15], insecticidal [16], trypanocidal [17], leishmanicidal [18], and antifungal [19,20]. Their therapeutic activity arises from the generation of oxidative stress through oxygen reactivity, as well as their redox activity and acid–base properties that promote apoptosis [15]. Their therapeutic activity arises mainly from redox cycling and the generation of reactive oxygen species (ROS), leading to oxidative stress and the disruption of vital processes such as respiration and DNA synthesis in *Mtb* [21,22]. It should be noted that substantial research and development have been conducted on hybrid molecules including two redox centers, notably quinones containing selenium. Among these researchers, given the varied biological actions of these two nuclei, it is also worth noting that a recent work by Ribeiro et al. [23] detailed the synthesis of novel selenium–menadione conjugates that showed outstanding efficacy against multidrug-resistant strains of *Mtb*, with compound **3** having a MIC of 0.8 µM (Figure 1b).

The triazole nucleus is another important family of compounds that has received a lot of interest due to its wide variety of therapeutic applications. These privileged structures, recognized as bioisosteres of amides, have been used to enhance the binding affinity and metabolic stability of bioactive molecules, exhibiting several biological activities, including anti-TB action [24,25]. For instance, Dutta and coworkers [26] reported that compounds **4** and **5** demonstrated a MIC of 0.78 µg/mL and 3.12 µg/mL (Figure 1c). Given the significance of organochalcogens, naphthoquinones, and the 1*H*-1,2,3-triazole scaffold, this study explores the impact of integrating these pharmacophoric entities within a single molecule, based on the medicinal chemistry strategies (hybridization). Here, this work presents the design, synthesis, and biological evaluation of a novel series of 4-((arylchalcogenyl)methyl)-1*H*-1,2,3-triazol-1-yl-menadione compounds as potential candidates for TB treatment, and through this approach, we aimed to integrate potentially enhanced biological activity via additive effects. The method involved a 1,3-dipolar cycloaddition reaction at room temperature between an azido–menadione and an alkyne derived from the chalcogen, with a copper salt acting as a catalyst, resulting in the exclusive formation of the 1,4-disubstituted isomer (Figure 1d).

To enhance the in vitro biological evaluations, in silico studies were performed to further investigate the potential of the synthesized compounds as anti-TB agents. With the growing threat of drug-resistant *Mtb*, the identification of novel drug targets is crucial for the development of effective new therapies. One promising target is protein kinase B (PknB), a serine/threonine kinase that plays a crucial role in mycobacterial growth and survival. PknB has been well documented as a key regulator of cell wall biosynthesis, making it a critical target for drug development against *Mtb* [27]. Therefore, in silico molecular docking simulations were performed to evaluate the binding affinity of the synthesized 4-((arylchalcogenyl)methyl)-1*H*-1,2,3-triazol-1-yl-menadione. This computational approach provided valuable insights into the potential interactions between the compounds and the target, helping to identify promising candidates for further experimental validation.

In addition to docking studies, the ADMET (absorption, distribution, metabolism, excretion, and toxicity) properties of the compounds were also predicted to assess their drug-likeness and pharmacokinetic profiles. ADMET predictions are a crucial step in drug discovery, as they provide early-stage insights into a compound’s potential for oral bioavailability, distribution within the body, metabolic stability, and toxicity [28]. These analyses allow for the identification of compounds with favorable properties, thus reducing the likelihood of late-stage failure in the drug development process. By combining both molecular docking and ADMET predictions, we aim to identify not only potent but also safe and viable drug candidates for the treatment of TB, particularly for overcoming the growing threat of drug-resistant strains.

## 2. Results and Discussion

**Scheme 1:**
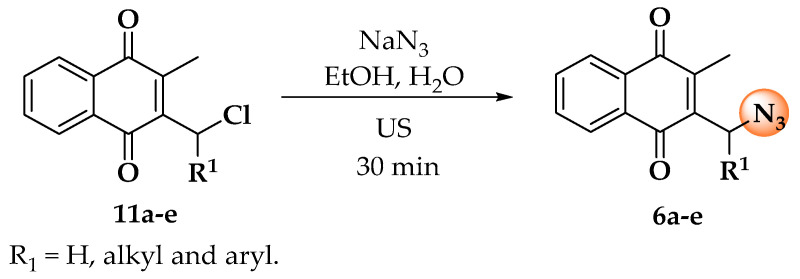
Synthesis of menadione–azides **6a**–**e** in ultrasound (US).

The synthesis of molecules started with the preparation of menadione–azides **6a**–**e**, which were obtained through a nucleophilic substitution reaction between sodium azide and chlorinated menadione derivatives **11a**–**e** [29,30], as described in the literature. These compounds were then used in the following reaction without prior purification (Scheme 1).

**Scheme 2:**
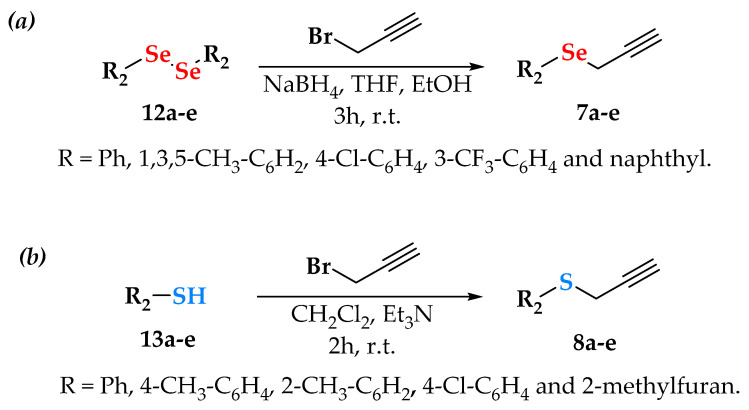
Synthesis of alkynes **7a**–**e** from diselenides (**a**) and **8a**–**e** from thiols (**b**).

Simultaneously, alkynes containing selenium **7a**–**e** and sulfur **8a**–**e** were synthesized using well-established procedures [31]. The propargyl selenides were prepared from aryl diselenides (**12a**–**e**) in an ethanol–THF solution, with sodium borohydride (NaBH_4_) as the reducing agent and propargyl bromide. These alkynes were obtained as yellowish oils after 3 h of reaction, with yields ranging from 30% to 85% (Scheme 2a) [32].

On the other hand, for the synthesis of propargylated sulfides **8a**–**e**, they were prepared by the method described by Denmark and coworkers [33]. The substituted thiols **13a**–**e** were treated with propargyl bromide in the presence of triethylamine (Et_3_N) to generate the desired products **8a**–**e**, all as yellowish oils, in good to excellent yields (46–95%, Scheme 2b). It is important to note that all chalcogen-propargylated compounds did not require purification and were obtained in satisfactory yields. Finally, with both intermediates (azides and alkynes) in hand, we proceeded to generate the desired **9a**–**i** and **10a**–**i**.

**Scheme 3:**
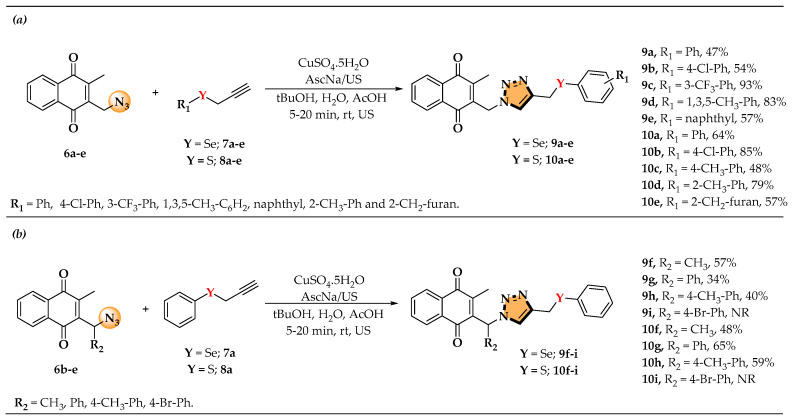
(**a**) Synthesis of products **9a**–**e** and **10a**–**e** obtained by varying the alkyne portion with different chalcogenides. (**b**) Synthesis of products **9f**–**i** and **10f**–**i** obtained by varying the azides. N.R. = No reaction. US = Ultrasound irradiation.

The triazole nucleus was chosen to connect two key molecular blocks—naphthoquinone (menadione) and organochalcogens (selenides and sulfides)—due to their importance in medicinal chemistry and the development of hybrid molecules [34]. To explore the synthetic scope, different variations in propargylated selenides and sulfides (**7a**–**e** and **8a–e**, respectively) were reacted with azides **6a**–**e** via click chemistry, yielding triazole-containing derivatives (**9a**–**i** and **10a**–**i**) (Scheme 3) [35,36].

Then, the influence of R_1_ and R_2_ on the efficiency of the click reaction leading to selenium- and sulfur-containing triazole derivatives was investigated. In general, compounds where R_2_ = H exhibited higher yields compared to their substituted counterparts. For instance, in the selenium series, **9a** (R_1_ = Ph, R_2_ = H) resulted in a 47% yield, while introducing a methyl group at R_2_ (**9f**, R_2_ = CH_3_) increased the yield to 57%, suggesting a slight positive effect. However, when R_2_ = Ph (**9g**), the yield significantly dropped to 34%, indicating that bulkier or more conjugated groups in this position negatively impact the reaction efficiency. A similar trend was observed in the sulfur-containing compounds, where **10a** (R_1_ = Ph, R_2_ = H) gave a yield of 64%, whereas **10f** (R_2_ = CH_3_) dropped to 48%, reinforcing the idea that any substitution at R_2_ tends to reduce reactivity. Notably, in both series, when R_2_ = 4-Br-Ph (**9i**, **10i**), no reaction was observed, suggesting a strong steric or electronic hindrance effect, preventing product formation. These results highlight that, while R_1_ variations lead to diverse outcomes, the most consistent trend is that any substitution at R_2_ tends to lower the reaction efficiency, particularly for bulkier or electron-withdrawing groups.

In summary, although no clear electronic or steric trends were observed for the aryl substituents, the nature of R_1_ had a more pronounced impact on reaction efficiency. Compounds with R_1_ = H consistently exhibited higher yields, whereas bulkier groups, particularly phenyl, significantly reduced yield. Further investigations, including computational modeling, could offer deeper insights into the steric and electronic factors influencing these transformations.

To confirm the structures of the desired compounds, all novel 4-((arylchalcogenyl)methyl)-1*H*-1,2,3-triazol-1-yl-menadione derivatives were characterized by ^1^H NMR spectroscopy. As an example, for the compound **9a**, in the high-field region, a singlet at 2.33 ppm with a relative integration of three hydrogens corresponds to the methyl group of menadione. For the aliphatic region linked to the naphthoquinone core, the CH_2_-type hydrogens attached to the selenium-containing moiety appear as a singlet at 4.05 ppm. Additionally, a singlet at 5.39 ppm (integrating for two hydrogens) corresponds to the methylene group adjacent to both the naphthoquinone core and the triazole moiety. Between these signals, at 7.19 ppm, a singlet integrating for one hydrogen is attributed to the triazole proton. In the aromatic region (8.04–7.03 ppm), four multiplets integrating for nine aromatic hydrogens confirm the presence of both the naphthoquinone ring and the aromatic system attached to selenium. These findings strongly support the structural assignment of **9a**.

To further validate the proposed structure, ^13^C NMR (APT mode) was performed, revealing all expected 21 carbon signals. The most shielded carbon, at 13.33 ppm, corresponds to the methyl group. At 20.74 ppm, a signal attributed to the methylene carbon linked to the selenium-containing moiety is observed, while at 44.95 ppm, a signal is assigned to another methylene carbon, in this case, attached to the naphthoquinone core. The characteristic triazole carbon appears at 122.89 ppm, and the remaining aromatic carbons are detected between 122.89 and 148.60 ppm. Finally, the two distinct carbonyl signals at 184.78 and 183.91 ppm confirm the presence of the naphthoquinone core, further corroborating the successful synthesis of **9a**.

As a result, it is important to highlight the rapid and effective synthesis achieved using this methodology, demonstrating its potential for identifying new molecules with biological activity. Furthermore, the newly synthesized compounds are air stable, easy to handle, and were thoroughly characterized using ^1^H and ^13^C NMR spectroscopy, as well as HRMS.

### 2.1. Antimycobacterial Evaluation

All chalcogen–naphthoquinones–1,2,3-triazoles hybrids **9a**–**h** and **10a**–**h** had their antimycobacterial activity evaluated, and isoniazid (INH), rifampicin (RIF), ethambutol (EMB), and pyrazinamide (PZA) were used as positive controls, with MICs of 0.12 µg/mL, 3.25 µg/mL, 1.00 µg/mL, and 100.06 µg/mL, respectively (Table 1). The inactivity of all the derivatives from series sulfur-containing compounds **10a**–**h** highlights the importance of selenium-containing compounds **9a**–**h** for the antimycobacterial activity, as most of them proved to be important inhibitors of *Mtb*. This is evident when comparing derivative **9a** with **10a**, where the sole difference in chemical structures was replacing the sulfur atom with selenium. Compound **9a** was active, showing a MIC of 6.23 µg/mL, equipotent to the drug ethambutol (EMB) (MIC 3.23 µg/mL), while **10a** was inactive with a MIC of 100.08 µg/mL.

Compound **9f** also exhibited a MIC of 6.23 µg/mL, with equally potent EMB. It is important to notice that the addition of methyl between the naphthoquinone and 1,2,3-triazole groups did not affect the anti-*Mtb* activity seen in **9f** (MIC 6.23 µg/mL) and **9a** (MIC 6.23 µg/mL), respectively. In contrast, introducing a bulky group like the phenyl group, as in compound **9g**, reduced the activity to a MIC of 12.49 µg/mL. Compound **9b**, containing a chloro substituent in the phenyl ring, was significantly more potent than the others, with a MIC of 3.20 µg/mL.

Given that the cell wall of *Mtb* is highly lipophilic, compounds with a higher LogP are expected to penetrate it more effectively [37]. Hence, the LogP of all compounds was computed to determine any potential correlation with antimycobacterial activity (Table 1). Compounds **9a**, **9b**, and **9f**, the most powerful in the series, are all more lipophilic than EMB. Although the correlation between higher lipophilicity and higher antimycobacterial activity did not allow us to notice a better activity between **9a** and **9f** than EMB, it suggests that the antimycobacterial activity of these substances is more related to structural aspects than to this physicochemical factor [38]. The lack of correlation between higher lipophilicity and higher antimycobacterial activity has also been reported elsewhere [39,40]. On the other hand, in addition to being more potent in vitro than EMB, the substance **9b** may also have improved pharmacokinetic profiles due to its increased lipophilicity, which can increase its volume of distribution and its half-life, which is short for EMB.

The six derivatives that are most potent, **9a**, **9b**, **9c**, **9f**, **9g,** and **9h,** were also screened against resistant strains of *Mtb* (Table 2). Compounds **9a**, **9b**, **9g,** and **9h** proved to be at least 7-fold more potent than INH against this strain, which shows that it is somehow able to partially bypass the mechanism of INH resistance of this mycobacterium. Unexpectedly, compound **9f** showed no activity against the *Mtb* strain T113/09, which exhibited resistance to INH (KatG mutation) and RIF (rpoB mutation), indicating that for this strain variety, introducing bulky groups between naphthoquinones and 1*H*-1,2,3-triazole should significantly enhance activity, as observed in the comparison of compounds **9f**, **9g**, and **9h**.

Compounds **9a**, **9b**, **9g**, and **9h** were assessed for cytotoxic effects on cell lines using the MTT method with the VERO cell line. This method measures metabolic activity via mitochondrial enzymes, and all compounds demonstrated minimal cytotoxicity.

### 2.2. In Silico Pharmacokinetic and Toxicity Profile (ADMET) Analysis

The antimycobacterial activity and ADMET profiles of compounds **9a**, **9b**, **9f**, and **9g** provide valuable insights into the relationship between structural features, biological activity, and pharmacokinetics. These findings emphasize the importance of a balanced approach to molecular design for optimizing both efficacy and drug-like properties in anti-TB agents.

The selection of compounds **9a**, **9b**, **9f**, and **9g** for analysis was based on their structural diversity and potential to provide meaningful comparisons of their biological and pharmacokinetic properties. Each compound represents distinct substituents and electronic characteristics designed to probe specific interactions with *Mtb* and evaluate their drug-like attributes. Compounds **9a** and **9b** were chosen due to their favorable predicted ADMET properties and preliminary biological activity. Compound **9f** was included to assess the effects of higher lipophilicity and bulkier substituents on efficacy and pharmacokinetics. Finally, **9g** was selected as a promising candidate with structural modifications aimed at improving potency and drug-likeness. This strategic selection enabled a comprehensive evaluation of structure–activity relationships (SAR) and the impact of ADMET parameters on biological performance.

Compound **9a** exhibited moderate antimycobacterial activity with a MIC of 6.76 µg/mL. Its physicochemical and pharmacokinetic properties are promising, including high predicted intestinal absorption (83.5%) and balanced lipophilicity (LogP: 3.92), supporting good oral bioavailability and cellular uptake. Additionally, **9a** demonstrated low toxicity risks, with a minimal inhibition of human liver microsomes (HLMs), which minimizes the likelihood of metabolic interactions. However, its lower potency compared to **9b** and **9g** highlights the potential for further structural refinements to improve its antimycobacterial efficacy without compromising its favorable ADMET profile.

Among the tested derivatives, **9b** displayed the most potent antimycobacterial activity, with a MIC of 3.20 µg/mL, comparable to the efficacy of the first-line drug ethambutol. The presence of a chlorine substituent at the *para*-position of the aromatic ring likely enhances interactions with the bacterial target. Its ADMET profile is similarly encouraging, with high intestinal absorption (88.8%), favorable blood–brain barrier (BBB) permeability, and moderate solubility, making it suitable for potential central nervous system (CNS) infections. Moreover, **9b** showed a low risk of mutagenicity and carcinogenicity, further solidifying its potential as a lead candidate for the development of novel anti-TB agents.

Compound **9f**, despite its high lipophilicity (LogP: 4.97), showed limited antimycobacterial activity, with a MIC > 43.64 µg/mL. The bulky phenyl substituent likely impairs both target binding and membrane permeability. ADMET analysis revealed moderate intestinal absorption (65.2%) and limited BBB permeability, which could restrict its utility for CNS infections. Additionally, the high lipophilicity may contribute to poor solubility, negatively affecting bioavailability. While **9f** displayed a favorable toxicity profile, its lack of efficacy highlights the necessity of significant structural modifications to enhance both its pharmacodynamic and pharmacokinetic properties.

Compound **9g** demonstrated excellent antimycobacterial activity, with a MIC of 3.12 µg/mL, surpassing that of **9a** and comparable to **9b**. Its high potency may be attributed to the optimized balance between lipophilicity (LogP: 4.43) and steric factors that facilitate efficient target interactions. The ADMET profile of **9g** further supports its potential, with high intestinal absorption (87.4%) and moderate BBB permeability, suggesting its applicability for systemic and CNS infections. Moreover, **9g** exhibited low toxicity, reinforcing its promise as a potent and safe anti-TB agent. The combination of superior activity and favorable ADMET characteristics positions **9g** alongside **9b** as a strong candidate for further development.

Compounds **9b** and **9g** emerged as the most promising derivatives, exhibiting superior antimycobacterial activity and favorable ADMET profiles, including high intestinal absorption, BBB permeability, and low toxicity. Compound **9a** showed moderate activity and a favorable safety profile, making it a potential candidate for optimization. In contrast, **9f** demonstrated limited efficacy, likely due to its structural features, which hinder its pharmacological and pharmacokinetic performance. These results highlight the critical interplay between structural design and ADMET optimization in the development of effective anti-TB agents. Future studies should focus on fine-tuning the structures of **9a**, **9b**, and **9g** to further enhance their therapeutic potential.

### 2.3. Molecular Docking Studies

Protein kinase B (PknB) is a serine/threonine kinase in *Mtb* that plays a critical role in modulating cell division and cell wall synthesis. It is essential for both in vitro growth and survival of the pathogen in vivo [41].

Given its pivotal role in the bacterial life cycle, PknB has been identified as a potential drug target for TB treatment. The inhibition of PknB has been shown to disrupt cell division and metabolism in *Mtb*, leading to growth arrest and cell death [42].

The compounds discussed in this study were explored with the aim of investigating possible interactions with the PknB active site, potentially shedding light on the mechanisms underlying their observed antimycobacterial activity. While the docking studies offer initial insights into these interactions, the biological relevance of these findings will require validation through in vitro experiments to provide more definitive data. This approach helps guide the ongoing development of these compounds, contributing to a deeper understanding of their potential mechanisms of action.

In this context, PknB represents a promising target, as its inhibition could interfere with the bacterial cell’s survival mechanisms. The binding interactions of the selenium-containing derivatives observed in the docking models suggest that these compounds might disrupt the key functions of PknB, offering a new avenue for TB treatment. However, a more comprehensive evaluation through experimental validation will be necessary to confirm the role of PknB and refine the potential of these compounds as therapeutic agents.

**Figure 2:**
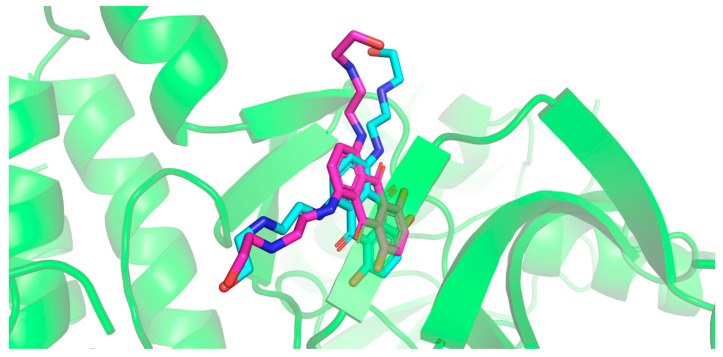
Superposition between co-crystallized ligand (IUPAC NAME: 1,4-dihydroxy-5,8-bis({2-[(2-hydroxyethyl)amino]ethyl}amino)-9,10-anthracenedione; IC_50_ = 800 nM—cyan structure) with the best RMSD value for redocking inside protein kinase B (PknB—magenta structure; PDB ID: 2FUM). Image generated by BIOVIA Discovery Studio Visualizer v24.1.0.23298 (Dassault Systèmes, 2023).

The redocking using the GoldScore function showed an RMSD = 1.6095 and a score = 64.0275, as demonstrated in Figure 2. The hydroxyl group on the right showed a different orientation compared to that of the co-crystallized ligand, while the rest of the structure showed satisfactory superposition.

**Figure 3:**
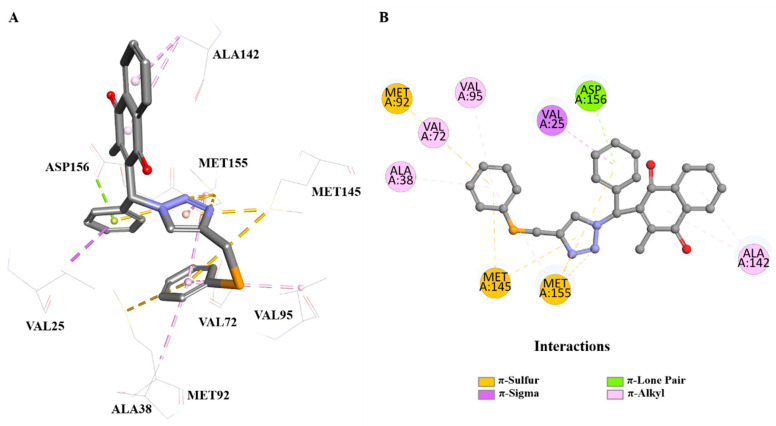
(**A**): Three-dimensional view of ligand–receptor interactions between PknB and (**9g**). (**B**): Two-dimensional diagram of ligand–receptor interactions between PknB and (**9g**) (PDB ID: 2FUM). Image generated by BIOVIA Discovery Studio Visualizer v24.1.0.23298 (Dassault Systèmes, 2023).

Compound **9g**, the selenium-containing derivative, demonstrated the highest docking performance, achieving a GoldScore of 68.98, surpassing the score of the co-crystallized ligand. Its binding interactions are crucially influenced by several key residues in the PknB active site, highlighting their importance in catalytic activity (Figure 3). The π–sulfur interactions with Met92, Met145, and Met155 are particularly significant, as these residues play vital roles in stabilizing the transition state and facilitating substrate binding in PknB. Additionally, the π–lone pair interaction with Asp156 is critical for coordinating the phosphorylation process, contributing to the enzyme’s catalytic function. The interactions with Ala38, Val72, Val95, and Ala142 through π–alkyl contacts further reinforce the binding affinity, providing additional stabilization to the ligand–receptor complex. These interactions with key catalytic residues in the PknB active site suggest that compound **9g** may effectively inhibit the kinase’s activity. The presence of selenium likely enhances the electronic properties of the compound, strengthening these interactions and contributing to its superior docking performance.

Compound **9b** also demonstrated a strong binding affinity to PknB, supported by its notable antimycobacterial activity (MIC = 3.20 µg/mL). The interactions of **9b** with the PknB active site involve several key residues (Figure S59), each playing an essential role in the enzyme’s catalytic function. The π–sulfur interactions with Met92 and Met155 are critical for stabilizing the enzyme–substrate complex and facilitating the enzyme’s phosphoryl transfer activity. The π–sigma bond formed with Met145 further contributes to the stability of the complex, as Met145 is involved in the stabilization of the transition state during the catalytic cycle. Additionally, the halogen interaction with Phe19 is particularly significant, as aromatic residues like Phe19 are often involved in the precise positioning of ligands within the binding site. The hydrogen bonding with Gly97 is also noteworthy, as Gly97 is crucial for maintaining the structural integrity of the binding pocket, and the consistent π–alkyl interactions with Val25, Val72, and Val95 enhance the overall binding affinity by providing further stabilization. These interactions suggest that the chlorine atom at the 4-position of the aromatic ring enhances binding specificity, likely improving the compound’s inhibitory potential against PknB (Figure S59).

Compounds **9g** and **9b**, both selenium-containing derivatives, demonstrated the most favorable docking scores among the series, with GoldScores of 68.98 and 67.21, respectively, both surpassing that of the co-crystallized ligand (GoldScore = 64.03). These results suggest stronger binding affinity and potential inhibitory action against PknB.

**Figure 4:**
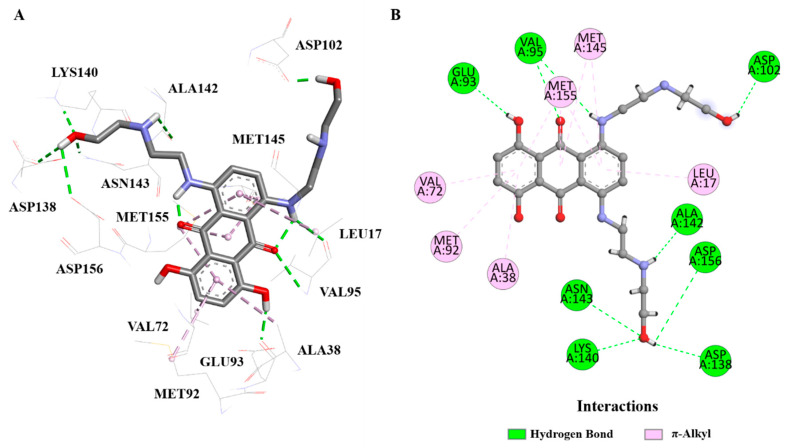
(**A**): 3D view of ligand–receptor interactions between PknB and the co-crystallized ligand. (**B**): 2D interaction diagram showing hydrogen bonds and π–alkyl interactions with key residues (PDB ID: 2FUM). Image generated using BIOVIA Discovery Studio Visualizer v24.1.0.23298 (Dassault Systèmes, 2023).

To provide a direct reference, the co-crystallized ligand (IUPAC name: 1,4-dihydroxy-5,8-bis({2-[(2-hydroxyethyl)amino]ethyl}amino)-9,10-anthracenedione) was redocked into the PknB active site. As shown in Figure 4, the ligand formed key hydrogen bonds with residues Asp138, Asn143, Asp156, and Asp102, as well as π–alkyl interactions with Met92, Met145, Met155, Val72, and Val95, which are essential for stabilizing the active site conformation. However, the central scaffold of the co-crystallized ligand showed a less optimal superposition compared to **9g** and **9b**, which may explain its lower docking score. These comparisons confirm that both **9g** and **9b** possess interaction patterns that mimic, and in some aspects exceed, those of the native ligand. The improved electronic interactions (e.g., π–sulfur and halogen bonding) and better spatial complementarity contribute to their stronger predicted binding to PknB.

### 2.4. Integrating Docking Results with Biological Activity

The docking outcomes align with the experimental MIC values, particularly for compounds **9b** and **9g**, which showed both potent antimycobacterial activity and high docking scores. The structural features of **9g** and **9b**, including the strategic placement of electron-donating and electron-withdrawing groups, appear to enhance their binding affinities to PknB, corroborating their roles as promising candidates for further development. Conversely, the suboptimal docking and biological profiles of **9f** highlight the need for structural modifications to optimize its target interactions.

These findings underline the critical interplay between molecular design, target engagement, and ADMET properties in advancing the development of novel anti-TB agents. Future studies will focus on leveraging these insights to refine the chemical structures of these derivatives, enhancing both their pharmacodynamic and pharmacokinetic profiles.

## 3. Materials and Methods

For the isolation and purification of the compounds via column chromatography, a glass column was used, with silica gel (0.063–0.2 mm mesh, Merck, Darmstadt, Germany) as the stationary phase. A suitable solvent or solvent mixture was employed as the eluent to achieve optimal separation. The fractions and compounds obtained were analyzed by thin-layer chromatography (TLC), using aluminum plates coated with silica gel 60 GF_254_, provided by Merck (Darmstadt, Germany), 0.25 mm thick and with particles between 5 and 40 µm in diameter. The substances separated on the chromatographic plates were visualized by using several development methods: in an iodine chamber, in an ultraviolet light chamber, or with a vanillin reagent followed by heating at 110 °C. Melting points were obtained on a Fisatom 430D (Sao Paulo, Brazil) apparatus and were uncorrected. All solvents and reagents used in the synthesis, purification, and characterization were purchased from commercial sources Sigma-Aldrich, Merck (Darmstadt, Germany), and Synth (Sao Paulo, Brazil) and used without prior purification. APPI-Q-TOFMS measurements were taken on a mass spectrometer equipped with an automatic syringe pump for sample injection. The ^13^C {^1^H} NMR spectra were obtained on a Bruker Advance NEO spectrometer (Bruker BioSpin, Rheinstetten, Germany), operating at 500 MHz, employing a direct broadband probe at 125 MHz. The mycobacterial cells were from Difco Laboratories, Detroit, MI, USA.

### 3.1. General Procedure

#### 3.1.1. General Procedure for the Synthesis of Propargyl Selenides **7a–e** [20]

In a 50 mL double-mouth flask with a nitrogen atmosphere, 0.5 mmol of diselenide was added, followed by 4 mL of dry tetrahydrofuran (THF) and 2 mL of dry ethanol. Then, 1 mmol of sodium borohydride (NaBH_4_) was added until the system was light in color. Finally, the mixture was cooled in an ice bath, and 1 mmol of propargyl bromide and 2 mL of dry THF were added. The reaction was kept under magnetic stirring for 3 h at room temperature. Finally, the reaction was extracted with water and dichloromethane, and the organic phase was dried with anhydrous sodium sulfate (Na_2_SO_4_). The solvent was then evaporated under vacuum. The desired products were purified by flash chromatography on silica gel using hexane as eluent.

#### 3.1.2. Characterization

Phenyl(prop-2-ynyl)selanyl (Compound **7a**). Yellow oil, yield 60%. ^1^H NMR (CDCl_3_, 500 MHz) δ (ppm): 7.60-7.56 (m, 2H), 7.31-7.27 (m, 3H), 3.49-3.46 (d, *J* = 2.7 Hz; 2H), 2.23-2.22 (t, *J* = 2.7 Hz; 1H).

(4-chlorophenyl)(prop-2-ynyl)selanyl (Compound **7b**). Yellow oil, yield 63%. ^1^H NMR (CDCl_3_, 500 MHz) δ (ppm): 7.54-7.52 (m, 2H), 7.29-7.26 (m, 2H), 3.48-3.45 (d, *J* = 2.7 Hz; 2H), 2.25-2.24 (t, *J* = 2.7 Hz; 1H).

(3-(trifluoromethyl)phenyl)(prop-2-ynyl)selanyl (Compound **7c**). Yellow oil, yield 46%. ^1^H NMR (CDCl_3_, 500 MHz) δ (ppm): 7.86-7.77 (m, 2H), 7.56-7.40 (m, 2H), 3.54-3.51 (d, *J* = 2.7 Hz; 2H), 2.27-2.26 (t, *J* = 2.7 Hz; 1H).

2,4,6-trimethyl-phenyl(prop-2-ynyl)selanyl (Compound **7d**). Orange oil, yield 30%. ^1^H NMR (CDCl_3_, 500 MHz) δ (ppm): 6.94 (m, 2H), 3.26-3.23 (d, *J* = 2.7 Hz; 2H), 2.57 (s, 6H), 2.27 (s, 3H), 2.15-2.14 (t, *J* = 2.7 Hz; 1H).

Naphthalen-2-yl(prop-2-ynyl)selanyl (Compound **7e**). Orange oil, yield 85%. ^1^H NMR (CDCl_3_, 500 MHz) δ (ppm): 8.41-8.39 (m, 1H), 7.92-7.91 (m, 1H), 7.85-7.83 (m, 2H), 7.59-7.50 (m, 2H), 7.42-7.39 (m, 1H), 3.52-3.49 (d, *J* = 2.7 Hz; 2H), 2.21-2.20 (t, *J* = 2.7 Hz; 1H).

#### 3.1.3. General Procedure for the Synthesis of Propargyl Sulfides **8a**–**e** [21]

In a 50 mL single-mouthed flask equipped with a magnetic stirring bar, 1.1 mmol of the appropriate thiophenol was added and dissolved in a solution of dichloromethane (10 mL) and 1.0 mmol of propargyl bromide. The liquid was then chilled in an ice bath before gradually adding 1.2 mmol of distilled triethylamine (Et_3_N). The reaction was stirred magnetically for 2 h at room temperature. Finally, the reaction was extracted with water and dichloromethane, and the organic phase was dried over anhydrous sodium sulfate (Na_2_SO_4_), while the solvent was removed under vacuum. The desired products did not require further purification.

#### 3.1.4. Characterization

Phenyl(prop-2-ynyl)sulfanyl (Compound **8a**). Yellow oil, yield 60%. ^1^H NMR (CDCl_3_, 500 MHz) δ (ppm): 7.46-7.44 (m, 2H), 7.33-7.30 (m, 2H), 7.26-7.22 (m, 1H), 3.60 (d, *J* = 2.7 Hz; 2H), 2.23-2.22 (t, *J* = 2.7 Hz; 1H).

(4-chlorophenyl)(prop-2-ynyl)sulfanyl (Compound **8b**). Yellow oil, yield 63%. ^1^H NMR (CDCl_3_, 500 MHz) δ (ppm): 7.40-7.38 (m, 2H), 7.31-7.29 (m, 2H), 3.58 (d, *J* = 2.7 Hz; 2H), 2.24-2.23 (t, *J* = 2.7 Hz; 1H).

Prop-2-ynyl(p-methylphenyl)sulfanyl (Compound **8c**). Yellow oil, yield 46%. ^1^H NMR (CDCl_3_, 500 MHz) δ (ppm): 7.39-7.37 (m, 2H), 7.15-7.13 (m, 2H), 3.56 (d, *J* = 2.6 Hz; 2H), 2.34 (s, 3H), 2.23-2.22 (t, *J* = 2.6 Hz; 1H).

Prop-2-ynyl(o-methyl phenyl)sulfanyl (Compound **8d**). Yellow oil, yield 65%. ^1^H NMR (CDCl_3_, 500 MHz) δ (ppm): 7.43-7.41 (m, 1H), 7.20-7.13 (m, 3H), 3.58 (d, *J* = 2.6 Hz; 2H), 2.40 (s, 3H), 2.22-2.20 (t, *J* = 2.6 Hz; 1H).

2-(prop-2-ynylthiol)furan (Compound **8e**). Yellow oil, yield 95%. ^1^H NMR (CDCl_3_, 500 MHz) δ (ppm): 7.36-7.33 (m, 1H), 6.32-6.28 (m, 1H), 6.22-6.15 (m, 1H), 3.18 (d, *J* = 2.6 Hz; 2H), 2.28-2.27 (t, *J* = 2.6 Hz; 1H).

#### 3.1.5. General Procedure for the Synthesis of Chalcogen–Naphthoquinones–1,2,3-Triazoles **9a**–**i** and **10a**–**i** [22]

In total, 0.25 mmol (1.0 eq) of azide and 0.3 mmol (1.2 eq) of propargyl chalcogenide were combined in a 10 mL single-mouthed flask with a tiny magnetic stir bar. They were then suspended in a 1:1 solution of water and *tert-*butanol (3 mL each). Two drops of acetic acid were then added. To this, 0.015 eq. of CuSO_4_.5H_2_O was added, followed by 0.15 eq. of sodium ascorbate. The mixture was agitated for 5 to 20 min at room temperature before being submitted to ultrasound. Finally, the reaction was extracted using water and ethyl acetate, and the solvent was then evaporated under vacuum. The desired products were purified by flash chromatography on silica gel using hexane/ethyl acetate as eluent.

#### 3.1.6. Characterization

2-methyl-3-((4-((phenylselanyl)methyl)-1*H*-1,2,3-triazolyl)methyl)naphthalene-1,4-dione (Compound **9a**), 0.1057 g, yield: 47%, yellow solid, m.p. 103 °C, ^1^H NMR (CDCl_3_, 500 MHz) δ (ppm): 8.06-8.01 (m, 2H), 7.71-7.67 (m, 2H), 7.35-7.34 (m, 2H), 7.19 (s, 1H), 7.09-7.02 (m, 3H), 5.39 (s, 2H), 4.05 (s, 2H), 2.33 (s, 3H). ^13^C NMR (CDCl_3_, 75 MHz) δ (ppm): 184.78, 183.91, 148.60, 145.73, 138.26, 134.35, 134.18, 133.68, 132.25, 131.57, 129.17, 127.57, 126.93, 126.71, 122.89, 44.95, 20.74, 13.33. HRMS (APPI+) *m*/*z*: calculated for C_21_H_17_N_3_O_2_Se [M+Na]^+^: 446.0384; found: 446,0383 (+1.08 ppm).

2-((4-(((4-chlorophenyl)selanyl)methyl)-1*H*-1,2,3-triazolyl)methyl)-3-methylnaphthalene-1,4-dione (Compound **9b**), 0.1142 g, yield: 54%, yellow solid, m.p. 118 °C, ^1^H NMR (CDCl_3_, 500 MHz) δ (ppm): 8.13-8.09 (m, 2H), 7.78-7.74 (m, 2H), 7.42 (s, 1H), 7.37-7.35 (m, 2H), 7.15-7.13 (m, 2H), 5.48 (s, 2H), 4.12-4.09 (s, 2H), 2.43 (s, 3H). ^13^C NMR (CDCl_3_, 75 MHz) δ (ppm): 184.43, 183.77, 148.44, 137.92, 134.83, 134.17, 134.04, 133.73, 132.03, 131.31, 129.18, 127.71, 126.79, 126.51, 45.24, 20.87, 13.16. HRMS (APPI+) *m*/*z*: calculated for C_21_H_16_ClN_3_O_2_Se [M+Na]^+^: 479.9994; found: 479.9988 (−0.10 ppm).

2-methyl-3-((4-((((3-(trifluoromethyl)phenyl)selanyl)methyl)-1*H*-1,2,3-triazolyl)methyl) naphthalene-1,4-dione (Compound **9c**), 0.1227 g, yield: 23%, dark brown oil, ^1^H NMR (CDCl_3_, 500 MHz) δ (ppm): 8.06-7.99 (m, 2H), 7.69-7.67 (m, 2H), 7.59 (s, 1H), 7.56-7.54 (m, 1H), 7.34-7.32 (m, 1H), 7.24-7.19 (m, 2H), 5.41 (s, 2H), 4.11-4.09 (s, 2H), 2.35 (s, 3H). ^13^C NMR (CDCl_3_, 75 MHz) δ (ppm): 184.52, 183.77, 148.45, 144.89, 137.94, 136.32, 134.16, 133.99, 132.03, 131.32, 130.85, 129.30, 126.74, 126.49, 125.88 (q, *J*_C−F_ = 271 Hz; CF_3_), 123.53 (q, *J*_C−F_ = 31 Hz; C_arom_), 122.86, 44.88, 20.61, 13.13. HRMS (APPI+) *m*/*z*: calculated for C_22_H_16_F_3_N_3_O_2_Se [M+Na]^+^: 514.0258; found: 514.0254 (+0.39 ppm).

2-((4-((2,4,6-trimethyl-phenylselanyl)methyl)-1*H*-1,2,3-triazolyl)methyl)-3-methylnaphthalene-1,4-dione (Compound **9d**), 0.1162 g, yield: 83%, orange solid, m.p. 113 °C, ^1^H NMR (CDCl_3_, 500 MHz) δ (ppm): 8.16-8.09 (m, 2H), 7.80-7.77 (m, 2H), 6.99 (m, 1H), 6.71 (m, 2H), 5.45 (s, 2H), 3.88-3.85 (s, 2H), 2.38 (s, 3H), 2.29 (s, 6H), 2.08 (s, 3H).^13^C NMR (CDCl_3_, 75 MHz) δ (ppm): 184.58, 183.64, 148.47, 145.68, 143.33, 138.47, 138.09, 134.18, 134.03, 132.04, 131.36, 128.28, 127.00, 126.78, 126.57, 121.87, 44.56, 24.20, 20.77, 19.52, 13.09. HRMS (APPI+) *m*/*z*: calculated for C_24_H_23_N_3_O_2_Se [M+Na]^+^: 488.0853; found: 488.0852 (+0.88 ppm).

2-methyl-3-((4-((naphthalenylselanyl)methyl)-1*H*-1,2,3-triazolyl)methyl)naphthalene-1,4-dione (Compound **9e**), 0.1182 g, yield: 57%, yellow solid, m.p. 127 °C, ^1^H NMR (CDCl_3_, 500 MHz) δ (ppm): 8.27-8.25 (m, 1H), 8.15-8.14 (m, 1H), 8.07-8.05 (m, 1H), 7.81-7.77 (m, 2H), 7.70-7.69 (m, 1H), 7.56-7.54 (m, 2H), 7.45-7.41 (m, 1H), 7.36-7.32 (m, 1H), 7.22-7.19 (m, 1H), 6.96 (m, 1H), 5.32 (s, 2H), 4.13 (s, 2H), 2.31 (s, 3H). ^13^C NMR (CDCl_3_, 75 MHz) δ (ppm): 184.55, 183.54, 148.33, 145.11, 137.82, 134.49, 134.18, 134.14, 133.95, 133.64, 132.10, 131.36, 128.95, 128.66, 128.41, 127.68, 126.69, 126.65, 126.52, 126.17, 125.60, 122.42, 44.54, 20.64, 13.14. HRMS (APPI+) *m*/*z*: calculated for C_25_H_19_N_3_O_2_Se [M+Na]^+^: 496.0540; found: 496.0538 (+0.67 ppm).

2-methyl-3-((4-((phenylthiol)methyl)-1*H*-1,2,3-triazolyl)methyl)naphthalene-1,4-dione (Compound **10a**), 0.0937 g, yield: 64%, orange solid, m.p. 124 °C, ^1^H NMR (CDCl_3_, 500 MHz) δ (ppm): 8.12-8.08 (m, 2H), 7.77-7.74 (m, 2H), 7.47 (m, 1H), 7.30-7.26 (m, 2H), 7.20-7.17 (m, 2H), 7.10-7.07 (s, 1H), 5.48 (s, 2H), 4.18 (s, 2H), 2.40 (s, 3H). ^13^C NMR (CDCl_3_, 75 MHz) δ (ppm): 184.30, 183.68, 148.78, 137.33, 136.58, 134.32, 134.17, 132.00, 131.37, 131.22, 129.41, 129.04, 127.52, 127.14, 126.86, 126.63, 45.45, 29.69, 13.22. HRMS (APPI+) *m*/*z*: calculated for C_21_H_17_N_3_O_2_S [M+Na]^+^: 398.0939; found: 398.0927 (−1.68 ppm).

2-((4-(((4-chlorophenyl)thiol)methyl)-1*H*-1,2,3-triazolyl)methyl)-3-methylnaphthalene-1,4-dione (Compound **10b**), 0.1022 g, yield: 85%, yellow solid, m.p. 112 °C, ^1^H NMR (CDCl_3_, 500 MHz) δ (ppm): 8.04-7.98 (m, 2H), 7.69-7.65 (m, 2H), 7.45 (s, 1H), 7.19-7.13 (m, 2H), 7.09-7.06 (m, 2H), 5.41 (s, 2H), 4.07 (s, 2H), 2.34 (s, 3H). ^13^C NMR (CDCl_3_, 75 MHz) δ (ppm): 184.43, 183.77, 148.44, 137.92, 134.83, 134.17, 134.04, 133.73, 132.03, 131.31, 129.18, 127.71, 126.79, 126.51, 45.24, 20.87, 13.16. HRMS (APPI+) *m*/*z*: calculated for C_21_H_16_ClN_3_O_2_S [M+Na]^+^: 432.0550; found: 432.0537 (−1.62 ppm).

2-methyl-3-((4-((*p*-methylphenylthiol)methyl)-1*H*-1,2,3-triazolyl)methyl)naphthalene-1,4-dione (Compound **10c**), 0.0972 g, yield: 54%, orange oil, ^1^H NMR (CDCl_3_, 500 MHz) δ (ppm): 8.09 (m, 2H), 7.76-7.72 (m, 2H), 7.45 (s, 1H), 7.19-7.17 (m, 2H), 6.99-6.98 (m, 2H), 5.48 (s, 2H), 4.12 (s, 2H), 2.38 (s, 3H), 2.22 (s, 3H). ^13^C NMR (CDCl_3_, 75 MHz) δ (ppm): 184.65, 183.88, 148.52, 144.67, 138.13, 134.29, 134.17, 133.99, 132.70, 132.14, 131.44, 131.27, 129.15, 126.89, 126.64, 123.35, 45.10, 29.22, 13.29. HRMS (APPI+) *m*/*z*: calculated for C_22_H_19_N_3_O_2_S [M+Na]^+^: 412.1096; found: 412.1084 (−1.50 ppm).

2-methyl-3-((4-((*o*-methylphenylthiol)methyl)-1*H*-1,2,3-triazolyl)methyl)naphthalene-1,4-dione (Compound **10d**), 0.0972 g, yield: 48%, yellow solid, m.p. 116 °C, ^1^H NMR (CDCl_3_, 500 MHz) δ (ppm): 8.12-8.08 (m, 2H), 7.77-7.74 (m, 2H), 7.42 (s, 1H), 7.24 (m, 1H), 7.04-7.01 (m, 2H), 6.99-6.96 (m, 1H), 5.48 (s, 2H), 4.14 (s, 2H), 2.40 (s, 3H), 2.27 (s, 3H). ^13^C NMR (CDCl_3_, 75 MHz) δ (ppm): 184.55, 183.96, 148.70, 138.52, 138.23, 137.61, 137.57, 134.58, 134.41, 134.23, 132.29, 132.24, 131.61, 131.46, 129.68, 127.10, 126.98, 126.87, 126.78, 126.67, 127.66, 45.68, 45.12, 29.92, 28.43, 13.54, 13.37. HRMS (APPI+) *m*/*z*: calculated for C_22_H_19_N_3_O_2_S [M+Na]^+^: 412.1096; found: 412.1083 (−1.75 ppm).

2-((4-(((furan-2-ylmethyl)thiol)methyl)-1*H*-1,2,3-triazolyl)methyl)-3-methylnaphthalene-1,4-dione (Compound **10e**), 0.0947 g, yield: 57%, yellow solid, m.p. 143 °C, ^1^H NMR (CDCl_3_, 500 MHz) δ (ppm): 8.12-8.09 (m, 2H), 7.77-7.73 (m, 2H), 7.62 (s, 1H), 7.33-7.32 (m, 1H), 6.27-6.26 (m, 1H), 6.22-6.21 (m, 1H), 5.54 (s, 2H), 3.74-3.72 (d, *J* = 10 Hz; 4H), 2.46 (s, 3H). ^13^C NMR (CDCl_3_, 75 MHz) δ (ppm): 184.59, 183.83, 148.48, 138.02, 134.16, 134.02, 132.05, 131.37, 126.75, 126.54, 110.40, 108.04, 45.01, 28.05, 25.67, 13.22. HRMS (APPI+) *m*/*z*: calculated for C_20_H_17_N_3_O_3_S [M+Na]^+^: 402.0889; found: 402.0878 (−1.19 ppm).

2-methyl-3-(1-(4-((phenylselanyl)methyl)-1*H*-1,2,3-triazolyl)ethyl)naphthalene-1,4-dione (Compound **9f**), 0,1091 g, yield: 57%, orange oil, ^1^H NMR (CDCl_3_, 500 MHz) δ (ppm): 8.09-8.07 (m, 1H), 8.01-7.99 (m, 1H), 7.73-7.71 (m, 2H), 7.51 (s, 1H), 7.48-7.45 (m, 2H), 7.23-7.17 (m, 3H), 6.08-6.04 (m, 1H), 4.17 (s, 2H), 2.17 (s, 3H), 1.96-1.95 (d, 3H). ^13^C NMR (CDCl_3_, 75 MHz) δ (ppm): 184.88, 183.69, 146.14, 142.50, 134.11, 133.79, 132.06, 131.94, 129.90, 129.26, 127.64, 126.74, 126.61, 54.47, 20.92, 18.15, 12.23. HRMS (APPI+) *m*/*z*: calculated for C_22_H_19_N_3_O_2_Se [M+Na]^+^: 460.0540; found: 460.0533 (−0.37 ppm).

2-methyl-3-(phenyl(4-((phenylselanyl)methyl)-1*H*-1,2,3-triazolyl)methyl)naphthalene-1,4-dione (Compound **9g**), 0.1246 g, yield: 34%, orange oil, ^1^H NMR (CDCl_3_, 500 MHz) δ (ppm): 8.12-8.10 (m, 1H), 8.00-7.99 (m, 1H), 7.76-7.72 (m, 2H), 7.47-7.43 (m, 2H), 7.35-7.32 (m, 5H), 7.19-7.16 (m, 3H), 7.02-7.01 (m, 2H), 4.16 (s, 2H), 2.14 (s, 3H). ^13^C NMR (CDCl_3_, 75 MHz) δ (ppm): 184.92, 183.57, 148.23, 141.45, 136.00, 134.18, 134.01, 132.05, 131.95, 129.63, 129.34, 129.22, 128.77, 127.68, 127.04, 126.88, 126.80, 61.71, 20.93, 13.42. HRMS (APPI+) *m*/*z*: calculated for C_27_H_21_N_3_O_2_Se [M+Na]^+^: 522.0697; found: 522.0692 (+0.15 ppm).

2-methyl-3-((4-((phenylselanyl)methyl)-1*H*-1,2,3-triazolyl)(*p*-methylphenyl)methyl) naphthalene-1,4-dione (Compound **9h**), 0,1281 g, yield: 54%, orange oil, ^1^H NMR (CDCl_3_, 500 MHz) δ (ppm): 8.12-8.10 (m, 1H), 8.00-7.98 (m, 1H), 7.74-7.71 (m, 2H), 7.47-7.44 (m, 2H), 7.26-7.25 (m, 1H), 7.19-7.15 (m, 6H), 6.96-6.94 (m, 2H), 4.16 (s, 2H), 2.36 (s, 3H), 2.12 (s, 3H). ^13^C NMR (CDCl_3_, 75 MHz) δ (ppm): 184.95, 183.61, 147.79, 145.13, 141.62, 138.88, 134.14, 133.95, 132.61, 132.04, 132.01, 130.06, 129.22, 127.65, 127.30, 126.85, 126.76, 61.71, 21.27, 20.94, 13.24. HRMS (APPI+) *m*/*z*: calculated for C_28_H_23_N_3_O_2_Se [M+Na]^+^: 536.0853; found: 536.0851 (+0.62 ppm).

2-methyl-3-(1-(4-((phenylthiol)methyl)-1*H*-1,2,3-triazolyl)ethyl)naphthalene-1,4-dione (Compound **10f**), 0,0971 g, yield: 48%, orange oil, ^1^H NMR (CDCl_3_, 500 MHz) δ (ppm): 8.09-8.07 (m, 1H), 8.01-7.98 (m, 1H), 7.73-7.70 (m, 2H), 7.65 (s, 1H), 7.34-7.31 (m, 2H), 7.26-7.21 (m, 2H), 7.16-7.13 (m, 1H), 6.09-6.05 (m, 1H), 4.23-4.22 (s, 2H), 2.14 (s, 3H), 1.98-1.97 (d, 3H). ^13^C NMR (CDCl_3_, 75 MHz) δ (ppm): 184.65, 183.44, 145.85, 144.66, 142.36, 135.39, 133.86, 131.83, 131.72, 129.91, 128.90, 126.52, 126.40, 121.89, 54.21, 29.11, 17.90, 11.96. HRMS (APPI+) *m*/*z*: calculated for C_22_H_19_N_3_O_2_S [M+Na]^+^: 412.1096; found: 412.1082 (−1.99 ppm).

2-methyl-3-(phenyl(4-((phenylthiol)methyl)-1*H*-1,2,3-triazolyl)methyl)naphthalene-1,4-dione (Compound **10g**), 0,1126 g, yield: 65%, orange oil, ^1^H NMR (CDCl_3_, 500 MHz) δ (ppm): 8.12-8.10 (m, 1H), 8.00-7.99 (m, 1H), 7.75-7.71 (m, 2H), 7.45-7.44 (m, 1H), 7.35-7.32 (m, 6H), 7.26-7.21 (m, 2H), 7.16-7.15 (m, 1H), 7.02-7.01 (m, 2H), 4.23 (s, 2H), 2.13 (s, 3H). ^13^C NMR (CDCl_3_, 75 MHz) δ (ppm): 184.69, 183.34, 148.01, 141.21, 135.72, 135.06, 133.97, 131.77, 131.72, 130.39, 129.15, 128.88, 128.59, 126.85, 126.66, 126.58, 61.54, 29.24, 13.17. HRMS (APPI+) *m*/*z*: calculated for C_27_H_21_N_3_O_2_S [M+Na]^+^: 474.1252; found: 474.1241 (−1.20 ppm).

2-methyl-3-((4-((phenylthiol)methyl)-1*H*-1,2,3-triazolyl)(*p*-methylphenyl)methyl)naphthalene-1,4-dione (Compound **10h**), 0,1161 g, yield: 59%, orange oil, ^1^H NMR (CDCl_3_, 500 MHz) δ (ppm): 8.11-8.09 (m, 1H), 7.99-7.97 (m, 1H), 7.82-7.78 (m, 1H), 7.74-7.70 (m, 2H), 7.33-7.30 (m, 2H), 7.27-7.26 (s, 1H), 7.24-7.21 (m, 2H), 7.17-7.14 (m, 3H), 6.96-6.94 (m, 2H), 4.21 (s, 2H), 2.36 (s, 3H), 2.10 (s, 3H). ^13^C NMR (CDCl_3_, 75 MHz) δ (ppm): 184.73, 183.39, 147.55, 144.27, 141.40, 138.71, 135.14, 133.92, 132.33, 131.81, 131.77, 130.33, 129.87, 128.87, 127.11, 126.63, 126.55, 61.54, 29.12, 21.13, 12.98. HRMS (APPI+) *m*/*z*: calculated for C_28_H_23_N_3_O_2_S [M+Na]^+^: 488.1409; found: 488.1404 (+0.16 ppm).

### 3.2. Antimycobacterial Evaluation

All the obtained molecules (**9a**–**h** and **10a**–**h**) had their antimycobacterial activity evaluated by Microplate Alamar Blue Assay (MABA) [43] against the *Mtb* strain H37Rv ATCC 27294 (American Type Culture Collection, Rockville, MD, USA) and INH/RIF-resistant Mtb isolates. The mycobacteria were grown in 100 mL of Middlebrook 7H9 broth (Difco Laboratories, Detroit, MI, USA) with a supplementation of 10% (*v*/*v*) OADC (oleic acid, albumin, dextrose, and catalase; Difco) and were incubated for 24 h on a rotary shaker at 150 rpm and 37 °C. After washing, the mycobacteria were suspended in 10 mL of 7H9 broth, adjusted to 0.1 Mc on the Farland scale, and then diluted 1:25. Subsequent 2-fold dilutions were performed in 100 µL of 7H9 broth in microplates. To minimize the evaporation of the medium in the test wells during incubation, 200 µL of sterile deionized water was added to all outer-perimeter wells of sterile 96-well plates (Falcon, 3072; Becton Dickinson, Lincoln Park, NJ, USA). The final compound concentrations tested were 0.01–10.0 µg/mL. Plates were incubated at 37 °C for 5 days. Finally, 25 µL of a 1:1 mixture of Alamar Blue (Accumed International, Westlake, OH, USA) reagent and 10% Tween 80 was added to the plate and incubated for an additional 24 h. Bacterial growth was determined by a pink color in the well; pink indicated no bacterial growth present, and purple indicated growth inhibition. The MIC was defined as the lowest drug concentration that was able to prevent a color change from blue to pink. Each assay was carried out in triplicate.

### 3.3. Cytotoxicity Evaluation

First, a VERO cell line suspension was prepared and distributed into each of the 96 wells of the culture plate, which was then incubated for 48 h to allow the cells to adhere. After this period, the suspension medium was discarded, and the cells were exposed to the substances under study, leaving them in the wells for a period of between 24 and 48 h. After this, the substance was discarded, and the cells were washed with a buffer, with MTT being added, followed by a 3 h incubation. After this period, the MTT was discarded, and Dimethyl sulfoxide (DMSO) was added to solubilize the formazan crystals generated. Finally, the absorbance of each well of the plate was obtained using a microplate spectrophotometer. The absorbance was quantified based on the readings that the equipment makes, based on the different intensities of the purple color in the wells of the plate; the more purple the well, the more viable cells there are.

### 3.4. ADMET Analysis

To evaluate the pharmacokinetic and toxicity profiles of the selected compounds, ADMET predictions were performed using the SwissADME [44] and pkCSM [45] online platforms. These tools provide a comprehensive assessment of absorption, distribution, metabolism, excretion, and toxicity properties based on cheminformatic models and experimental datasets.

The chemical structures of the ligands were prepared using MarvinSketch 24.1.0 [46] at the physiological pH of the mycobacterial cytoplasm (pH = 7.4). The ligands were exported in SMILES format, as required for ADMET analysis tools.

The pharmacokinetic parameters analyzed included the following:

- Absorption: gastrointestinal (GI) absorption, P-glycoprotein (P-gp) substrate classification, and skin permeability (log Kp).
- Distribution: blood–brain barrier (BBB) penetration and volume of distribution at steady state (VDss).
- Metabolism: prediction of cytochrome P450 enzyme inhibition (CYP450 isoforms: 1A2, 2C9, 2C19, 2D6, and 3A4).
- Excretion: Total clearance and renal clearance predictions.

To ensure safety profiles, the following toxicity parameters were analyzed:

- Hepatotoxicity.
- Mutagenicity (AMES test).
- Carcinogenicity.
- Potential for cardiotoxicity (hERG channel inhibition).

The ADMET results were compiled to identify compounds with favorable pharmacokinetic properties and minimal toxicity risks. Special attention was given to balancing lipophilicity, solubility, and permeability to maximize drug-likeness.

### 3.5. Molecular Docking

Because protein kinase B (PknB) is essential for mycobacterial growth, the target has undergone several inhibition studies in the literature [47,48]. For this reason, the structure deposited in the Protein Data Bank (PDB) of the catalytic domain of protein kinase PknB from *Mtb* (PDB ID: 2FUM) [49] in a complex with mitoxantrone (MIX) was chosen for molecular docking analysis. The Hermes GOLD software (Genetic Optimization for Ligand Docking, version 2024.1.0) with the GoldScore function was used. The protein was prepared using the GOLD Wizard tool, excluding water molecules not belonging to the active site, the co-crystallized ligand, and double conformations. Hydrogen atoms were added, and the active site was configured by selecting a 6 Å sphere from the co-crystallized ligand cavity. The ligands were prepared using the physiological pH of the mycobacterial cytoplasm (pH 7.4), using MarvinSketch 24.1.0 [50]. The three-dimensional structure was generated in Avogadro 1.2.0. In Avogadro 1.2.0, a preoptimization was performed using the UFF force field until ΔE < 10^−3^ kJ mol^–1^ [51].

## 4. Conclusions

This work describes the effective generation of a new series of sixteen 4-(arylchalcogenyl)methyl-1*H*-1,2,3-triazol-1-yl-menadione compounds. These compounds were synthesized through a Cu(I)-catalyzed 1,3-dipolar cycloaddition reaction involving various menadione–azides and chalcogen–alkynes, resulting in yields ranging from 34% to 93%. The rapid and efficient synthesis achieved using this methodology underscores its potential for discovering novel biologically active molecules. Furthermore, the synthesized compounds possess air stability and are easy to handle. All compounds were characterized using ^1^H and ^13^C NMR spectroscopy, as well as HRMS.

The selenium-containing series displayed significant antimycobacterial properties, highlighting the crucial role of selenium in their anti-*Mtb* efficacy. Among synthesized compounds, **9a**, **9b**, **9c**, **9f**, **9g**, and **9h** exhibited greater potency against the susceptible strain (H37Rv) than the first-line TB drug EMB. Additionally, **9a**, **9b**, **9g**, and **9h** demonstrated higher efficacy than INH against drug-resistant *Mtb*. Even at concentrations of up to 100 µg/mL, these derivatives proved to be non-toxic. Molecular docking and ADMET analysis further supported the promising therapeutic potential of these compounds. The docking studies revealed favorable interactions of the compounds with key targets in *Mtb*, particularly protein kinase B (PknB), an essential enzyme for mycobacterial growth. ADMET predictions highlighted the favorable pharmacokinetic properties of these compounds, including high intestinal absorption, low toxicity, and promising metabolic stability. The observed activity of these selenium-containing compounds toward *Mtb* strains, including drug-resistant ones, coupled with their favorable safety profile at concentrations of up to 100 µg/mL, suggests a promising avenue for the development of novel anti-TB agents. Further investigations into the mechanisms underlying their enhanced antimycobacterial activity and their potential as part of combination therapy regimens, supported by in silico findings, could contribute significantly to the fight against TB. 

## Figures and Tables

**Table 1:** Antimycobacterial evaluation of the new of chalcogen–naphthoquinones-1*H*–1,2,3-triazoles **9a**–**h** and **10a**–**h** against *Mtb* H37Rv.

Entry	Structure	MIC (µg/mL) ^a^	cLogP ^b^	Entry	Structure	MIC (µg/mL) ^a^	cLogP ^b^
**9a**	** 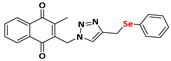 **	≤6.23	4.10	**10a**	** 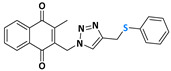 **	100.08	3.73
**9b**	** 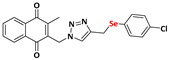 **	≤3.20	4.82	**10b**	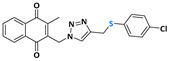	>100.03	4.50
**9c**	** 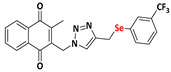 **	≤49.91	4.99	**10c**	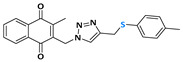	>100.19	4.23
**9d**	** 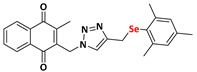 **	>99.86	5.60	**10d**	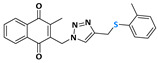	>100.19	4.23
**9e**	** 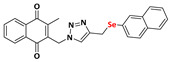 **	99.90	5.28	**10e**	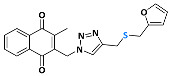	>100.14	3.30
**9f**	** 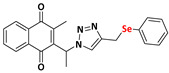 **	≤6.23	4.41	**10f**	** 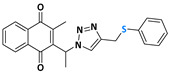 **	100.21	4.04
**9g**	** 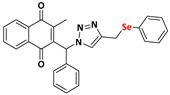 **	≤12.49	5.65	**10g**	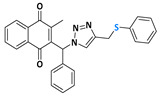	>100.14	5.28
**9h**	** 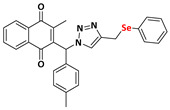 **	24.94	6.15	**10h**	** 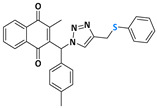 **	>100.19	5.78
INH ^c^		0.12	−0.71	RIF ^d^		1.00	3.85
EMB ^e^		3.26	−0.12	PZA ^f^		100.06	−0.71

^a^ sensitive strain to all first- and second-line drugs against TB. The error was calculated by performing each reaction in triplicate. ^b^ clogP was calculated using ChemBioDraw Ultra 13; ^c^ isoniazid (INH), ^d^ rifampicin (RIF), ^e^ ethambutol (EMB), and ^f^ pyrazinamide (PZA).

**Table 2:** Antimycobacterial evaluation of six derivatives **9a**, **9b**, **9c**, **9f**, **9g,** and **9h** against resistant *Mtb* strains.

Compound	MIC (µg/mL) INH/RIFR-*Mtb* ^a^
**9a**	3
**9b**	≤3
**9c**	>99
**9f**	50
**9g**	≤3
**9h**	≤3
INH	8

^a^ INH- and RIF-resistant *Mtb* (strain T113/09, KatG and rpoB mutation).

## Data Availability

Data are contained within this article and Supplementary Materials.

## Supplementary Materials

The following supporting information can be downloaded at: https://www.mdpi.com/article/10.3390/ph18060797/s1, Figures: Spectroscopic characterization for the Synthesized Compounds.
