# Supplementary material for: Design, Synthesis, and In Vitro Evaluation of 4-(Arylchalcogenyl)methyl)-1H-1,2,3-triazol-1-yl-menadione: Exploring Their Potential Against Tuberculosis
Source: Pharmaceuticals (Basel). 2025 May 26;18(6):797. doi: 10.3390/ph18060797 (PMC12195982; doi:10.3390/ph18060797)
Supplement: Supplementary file 1 [file pharmaceuticals-18-00797-s001.zip › pharmaceuticals-3649661-supplementary.pdf]

Electronic Supplementary Information (ESI) for

Design, Synthesis, and *In Vitro* Evaluation of 4-(arylchalcogenyl)methyl)-1*H*-1,2,3-triazol-1-yl-menadione: Exploring Their Potential Against *Tuberculosis*

Nathália L. B. Santos,[a] Luana S. Gomes,[a] Ruan C. B. Ribeiro,[a] Alcione S. de Carvalho,[a] Maria Cristina S. Lourenço,[b] Laís Machado Marins,[a] Sandy Polycarpo Valle,[a] Thiago H. Doring,[c,d] Adriano D. Andricopulo,[d] Aldo S. de Oliveira,[e] Vitor F. Ferreira,[f] Fernando C. da Silva,[a*] Luana da Silva Magalhães Forezi[a], and Vanessa Nascimento[a*]

[a]Universidade Federal Fluminense, Instituto de Química, Campus do Valonguinho, 24020-141, Niterói-RJ, Brasil.
[b]Laboratório de Bacteriologia e Bioensaios, Campus Manguinhos – Fiocruz – Fiocruz, 21040-361, Rio de Janeiro-RJ, Brasil.
[c]Departamento de Ciências Exatas e Educação (CEE), Centro Tecnológico, de Ciências Exatas e Educação (CTE), Universidade Federal de Santa Catarina (UFSC), Blumenau 89036-256, SC, Brasil.
[d]Laboratório de Química Medicinal e Computacional (LQMC), Instituto de São Carlos de Física (IFSC), Universidade de São Paulo (USP), Av. João Dagnone, 1100, 13563-120, São Carlos, SP, Brasil.
[e]Instituto Gulbenkian Institute de Medicina Molecular (GIMM), Faculdade de Medicina, Universidade de Lisboa, 1649-028, Lisboa, Portugal.
[f]Faculdade de Farmácia, Departamento de Tecnologia Farmacêutica, 24241-000, Niterói-RJ, Brasil.

Contents	Page
^{1}H, ^{13}C NMR Spectra and HRMS (APPI+)	**S2**
Molecular docking studies	**S39**

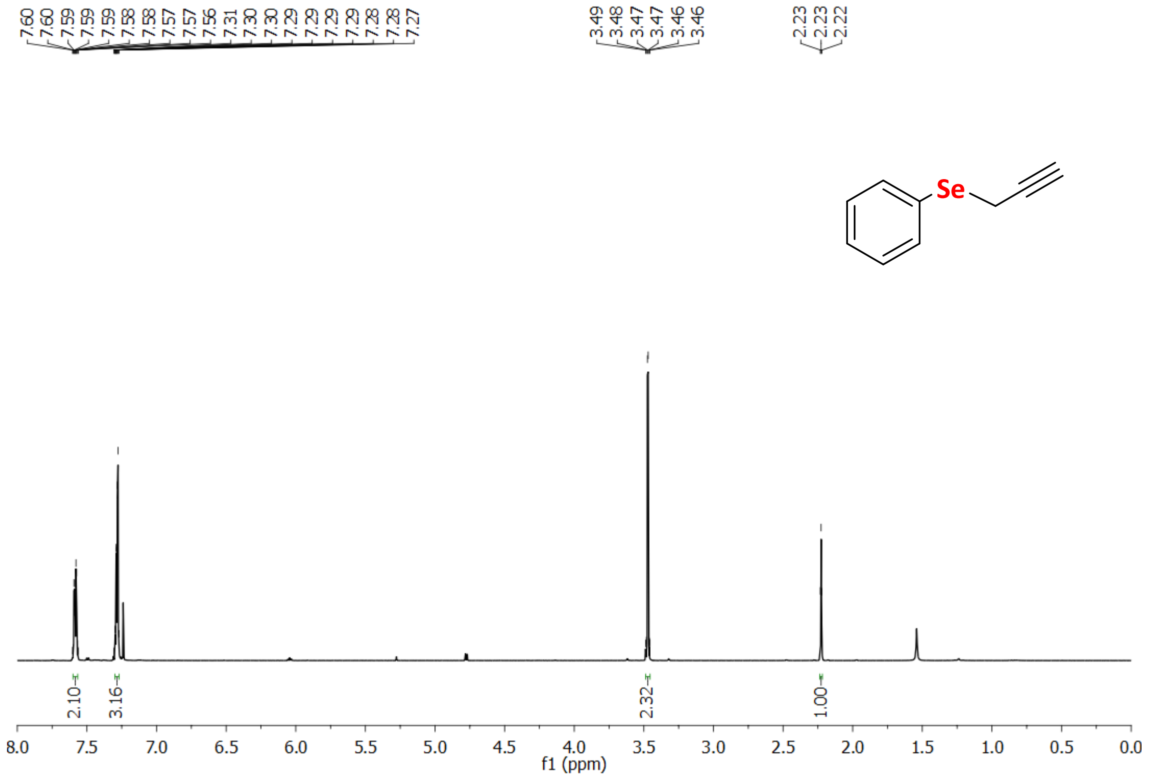

Figure S1. ^{1}H NMR spectrum of compound **7a** in $CDCl_3$ at 500MHz.

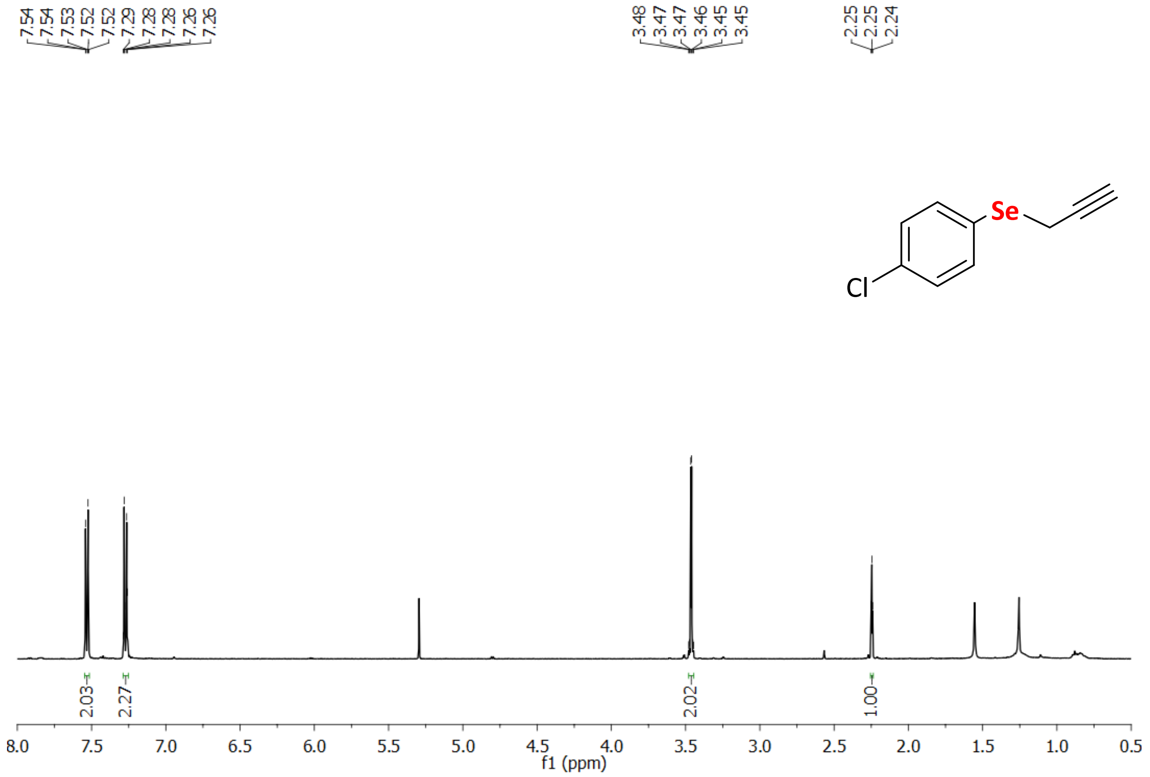

Figure S2. ^{1}H NMR spectrum of compound **7b** in $CDCl_3$ at 500MHz.

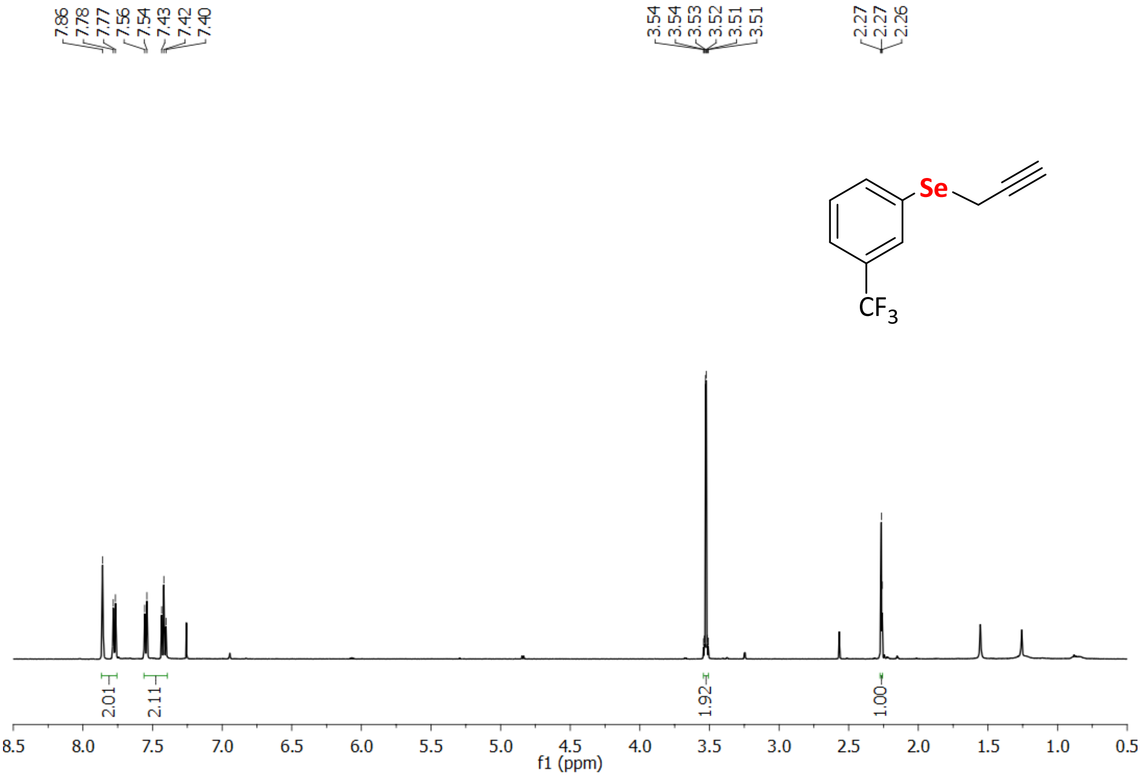

Figure S3. ^{1}H NMR spectrum of compound **7c** in $CDCl_3$ at 500MHz.

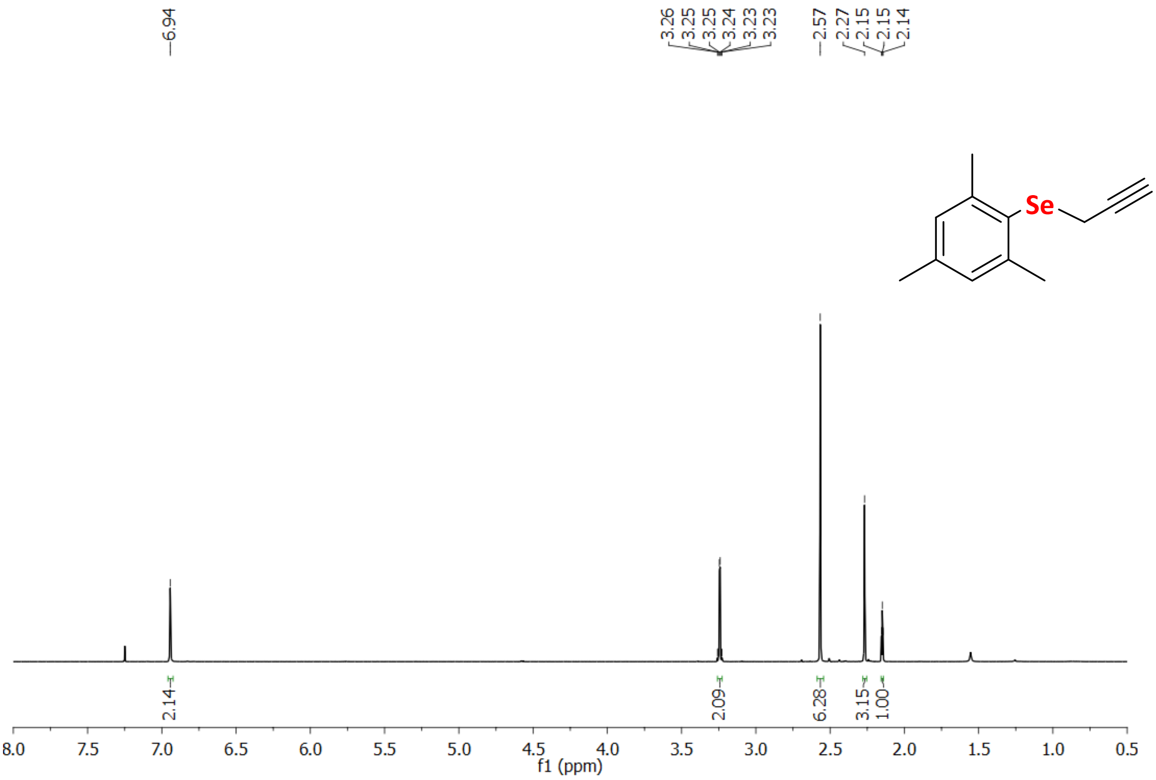

Figure S4. ^{1}H NMR spectrum of compound **7d** in $CDCl_3$ at 500MHz.

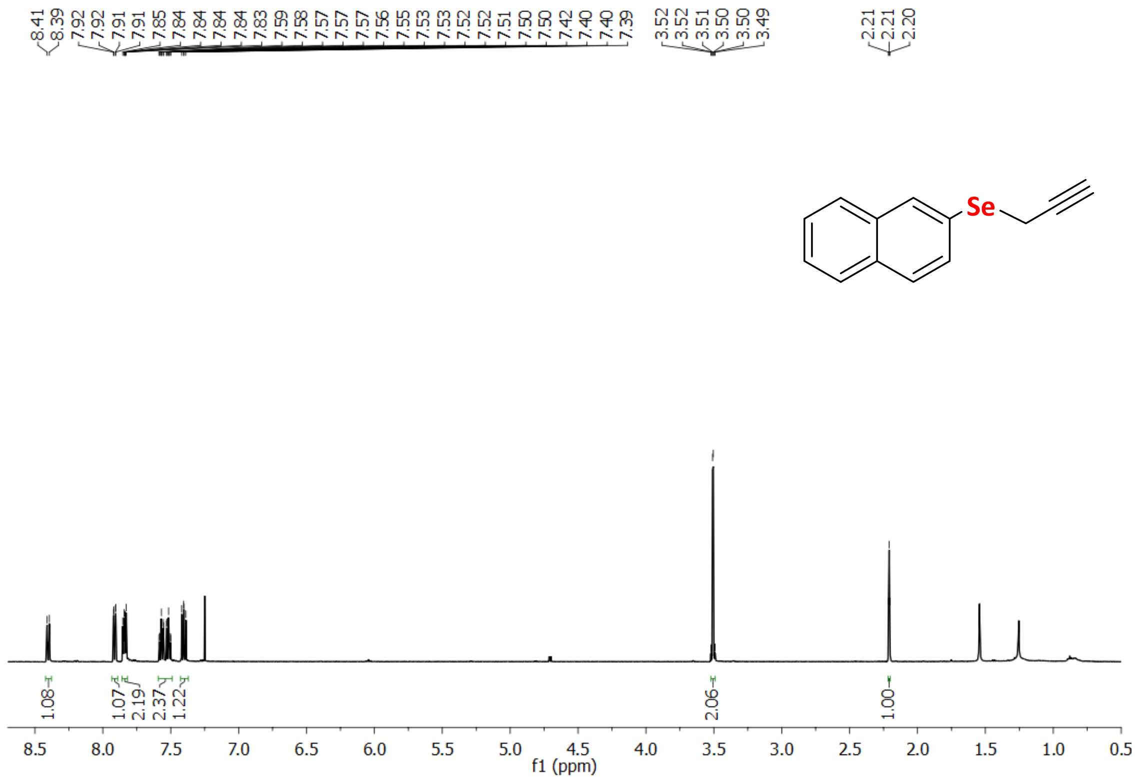

Figure S5. ^{1}H NMR spectrum of compound **7e** in $CDCl_3$ at 500MHz.

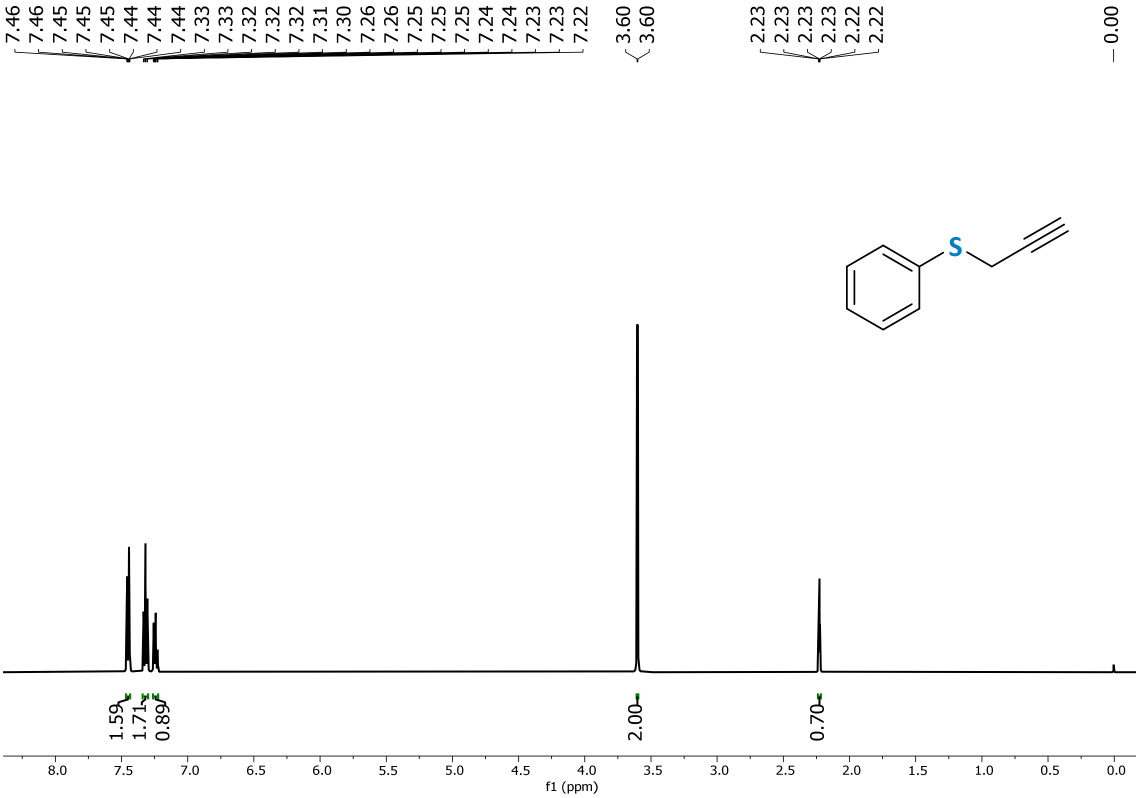

Figure S6. ^{1}H NMR spectrum of compound **8a** in $CDCl_3$ at 500MHz.

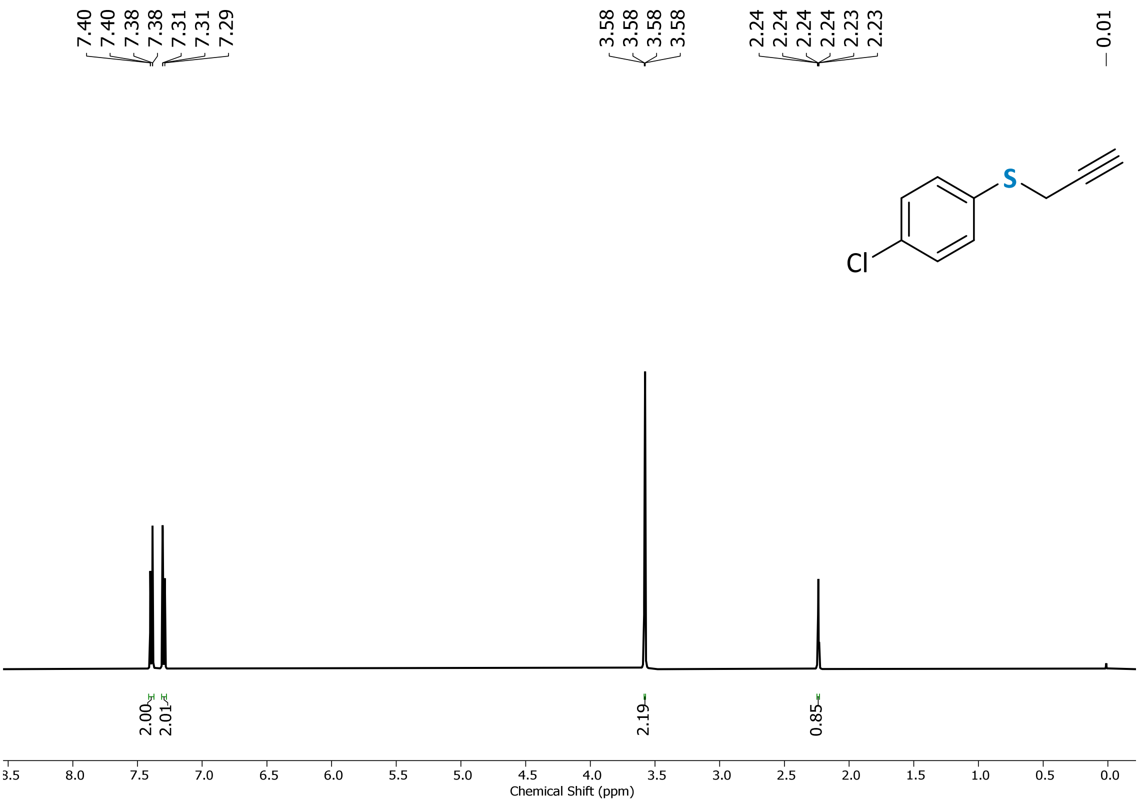

Figure S7. ^{1}H NMR spectrum of compound **8b** in $CDCl_3$ at 500MHz.

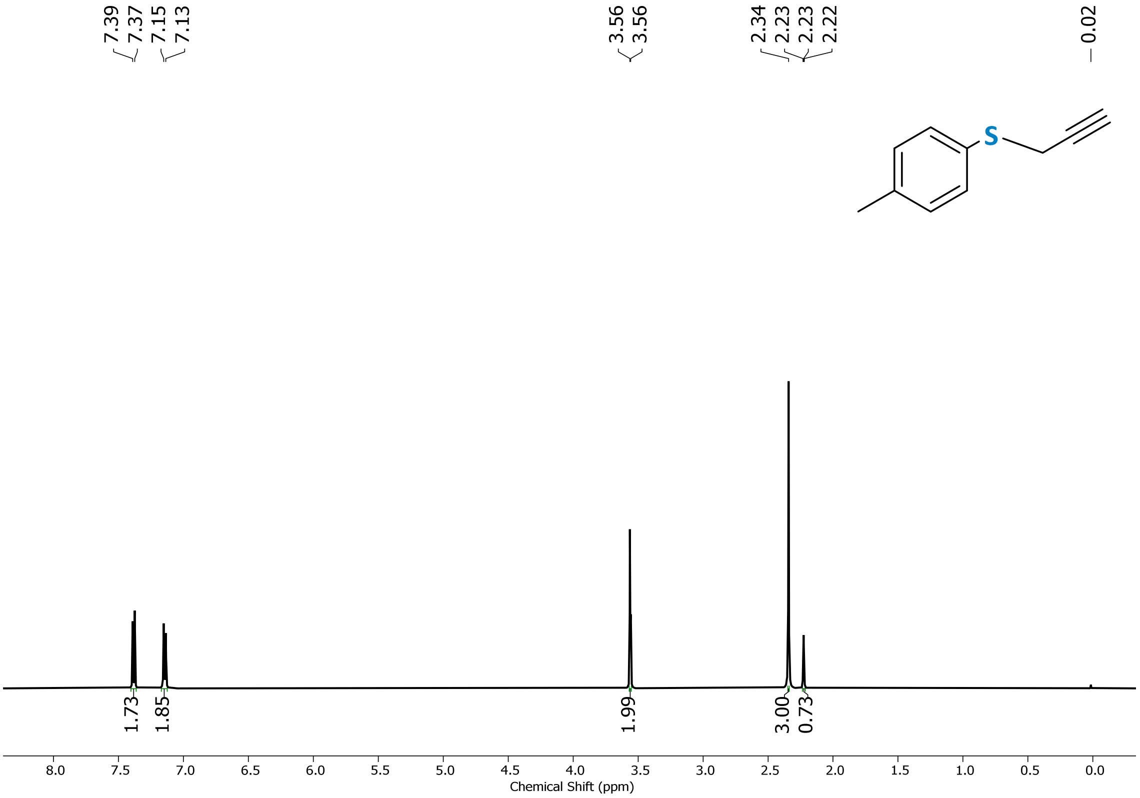

Figure S8. ^{1}H NMR spectrum of compound **8c** in $CDCl_3$ at 500MHz.

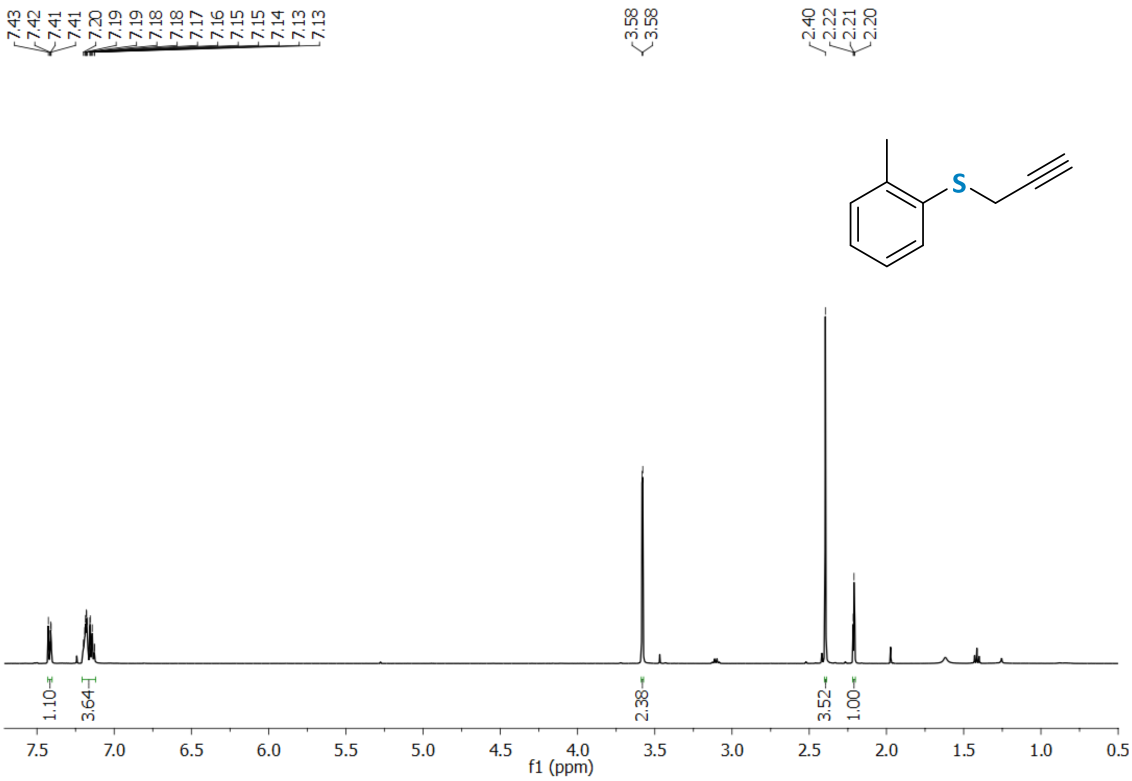

Figure S9. ^{1}H NMR spectrum of compound **8d** in $CDCl_3$ at 500MHz.

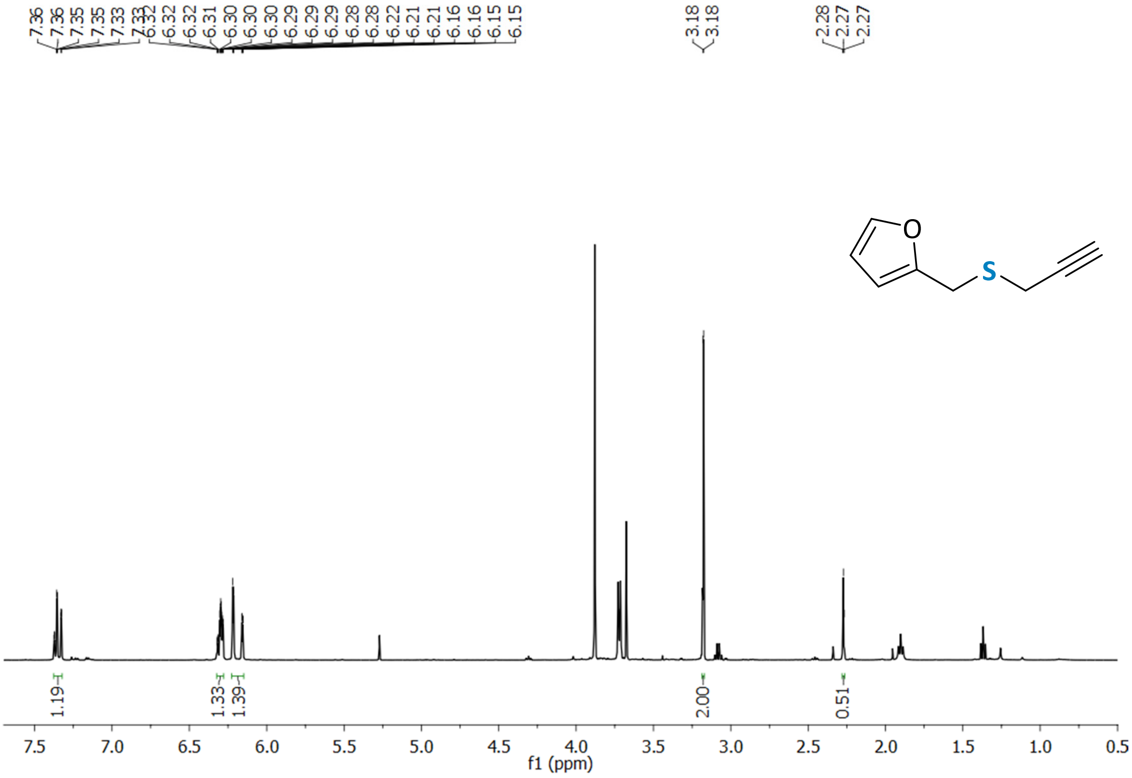

Figure S10. ^{1}H NMR spectrum of compound **8e** in $CDCl_3$ at 500MHz.

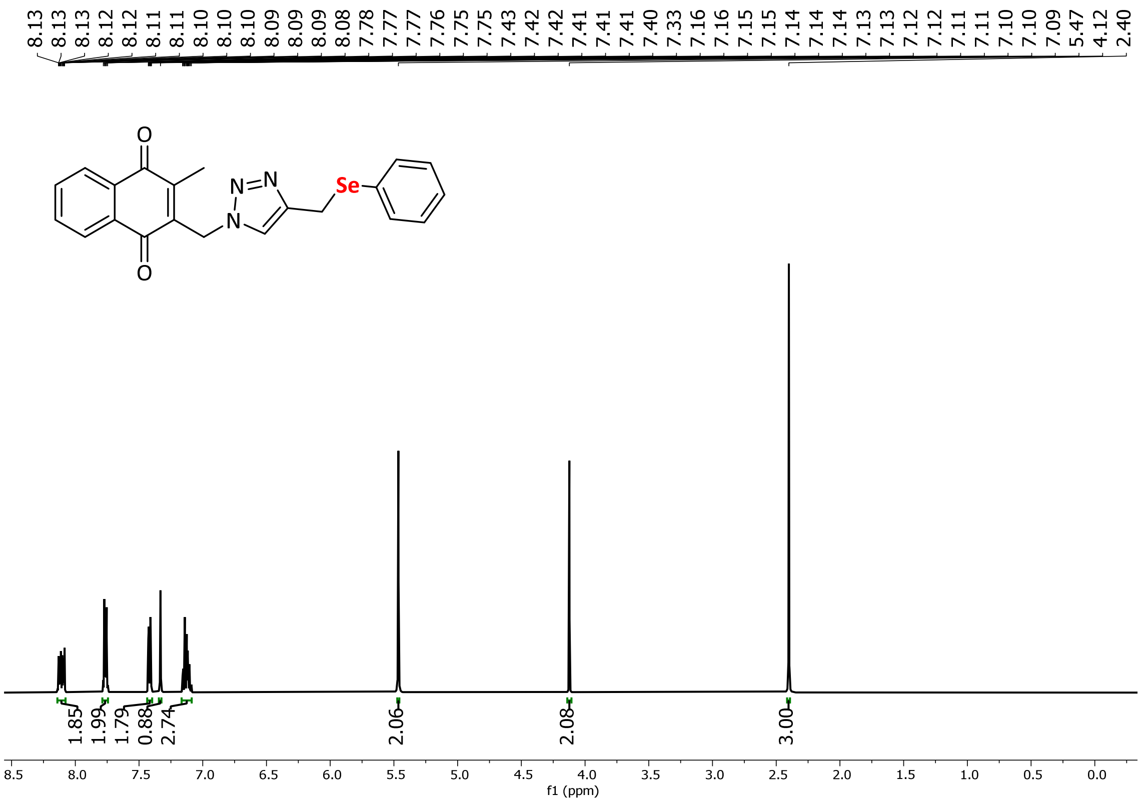

Figure S11. ^{1}H NMR spectrum of compound **9a** in $CDCl_3$ at 500MHz.

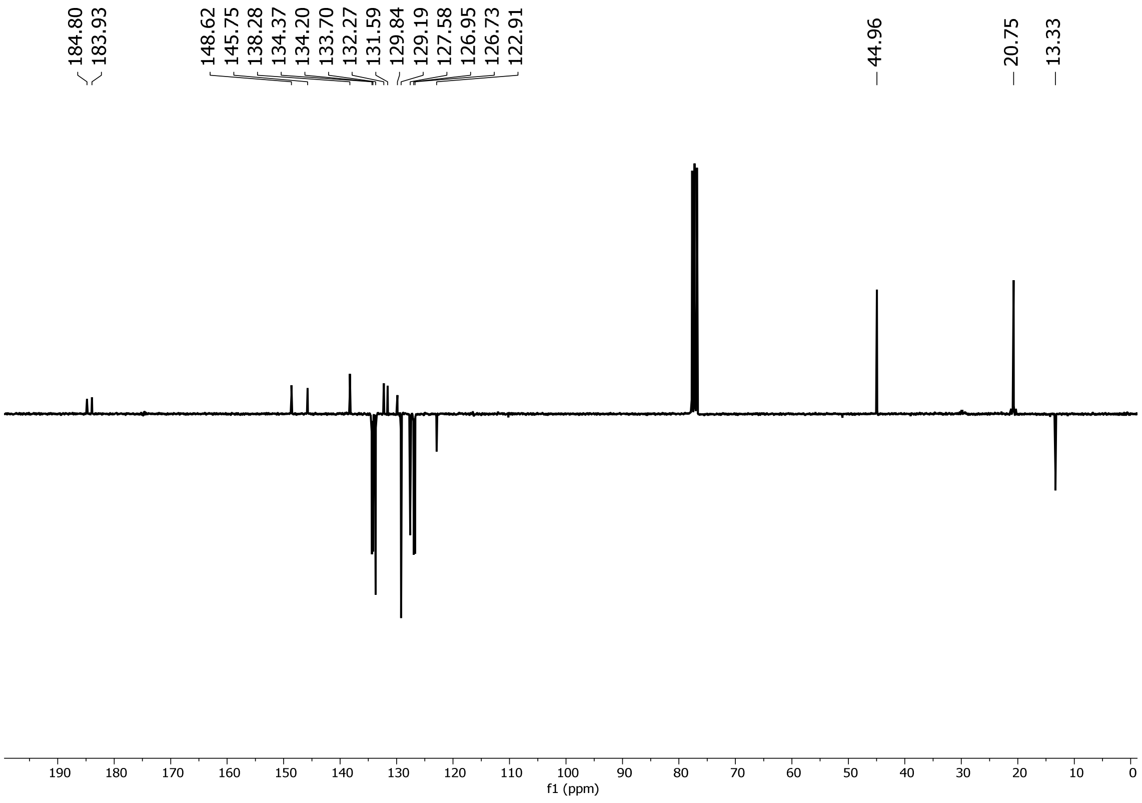

Figure S12. ^{13}C-APT NMR spectrum of compound **9a** in $CDCl_3$ at 75MHz.

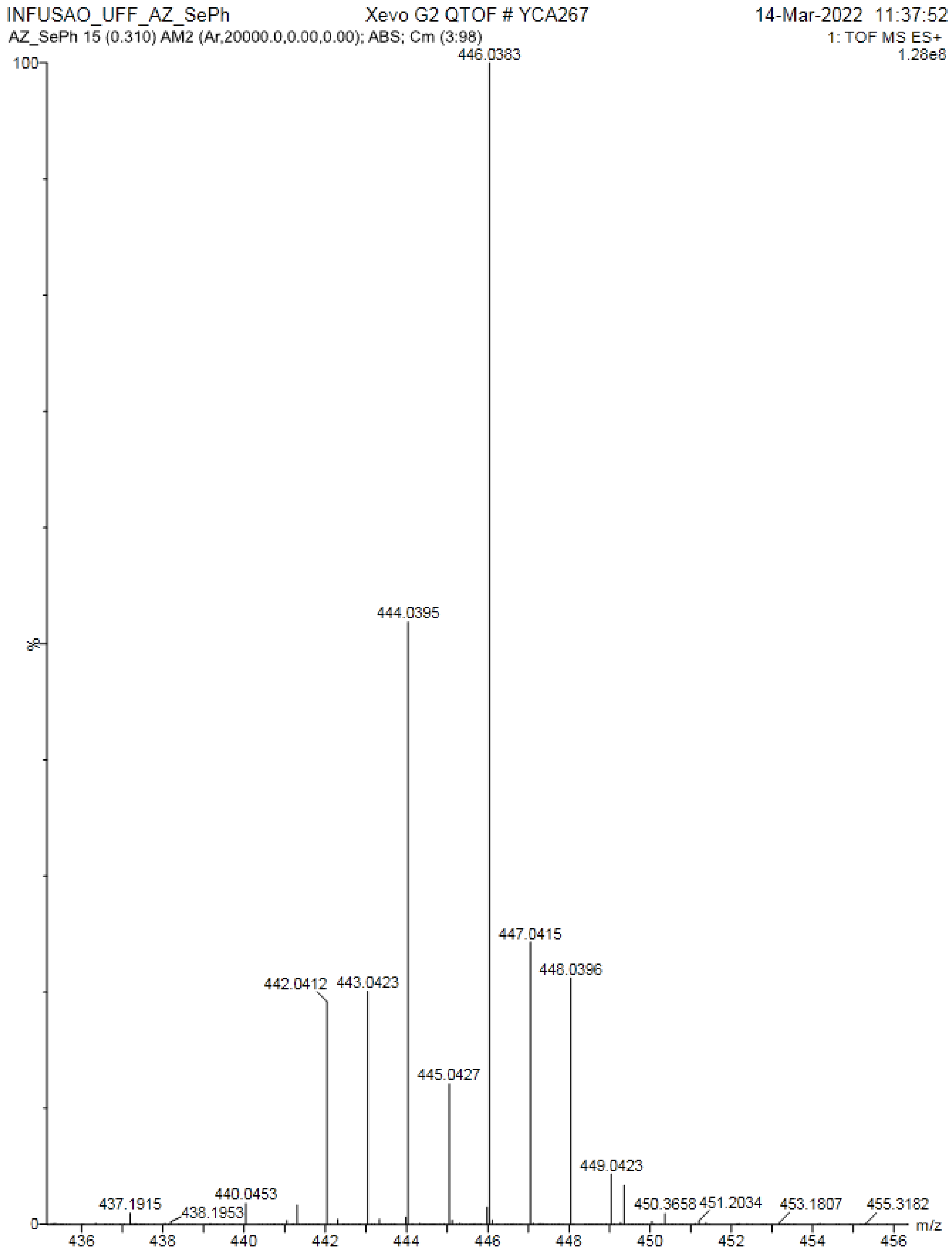

Figure S13. ESI MS spectrum of **9a**.

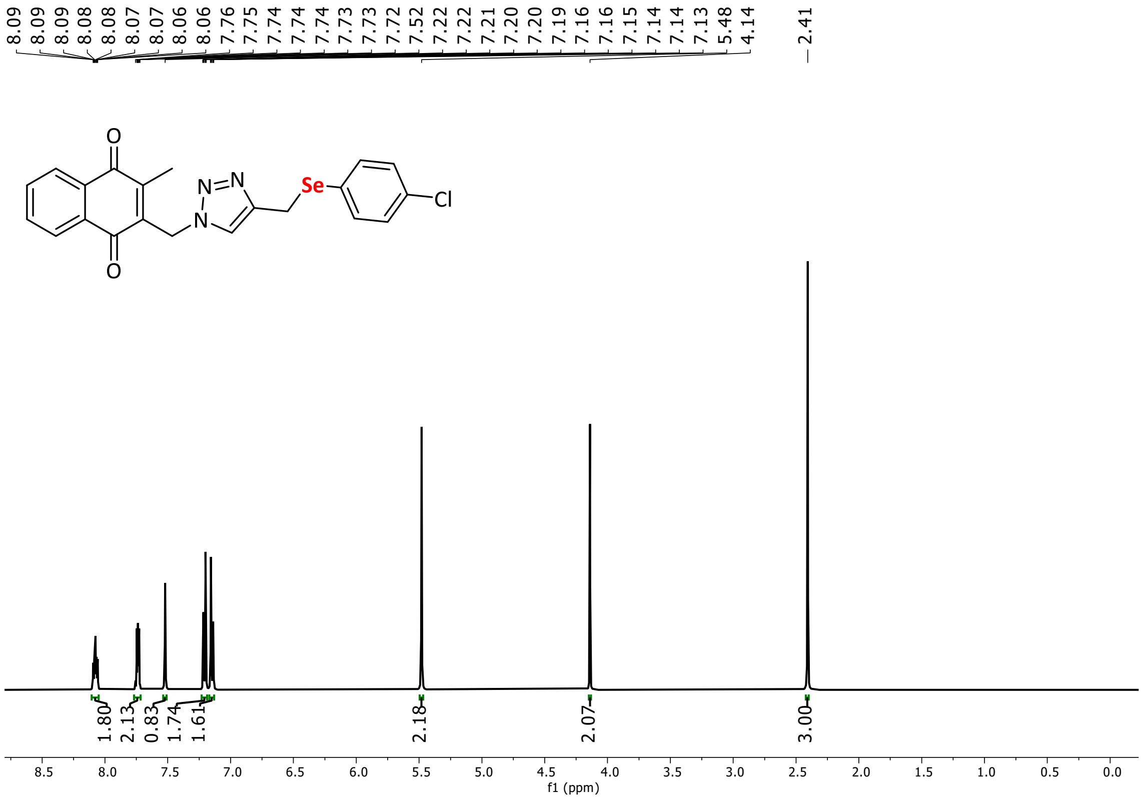

Figure S14. ^{1}H NMR spectrum of compound **9b** in $CDCl_3$ at 500MHz.

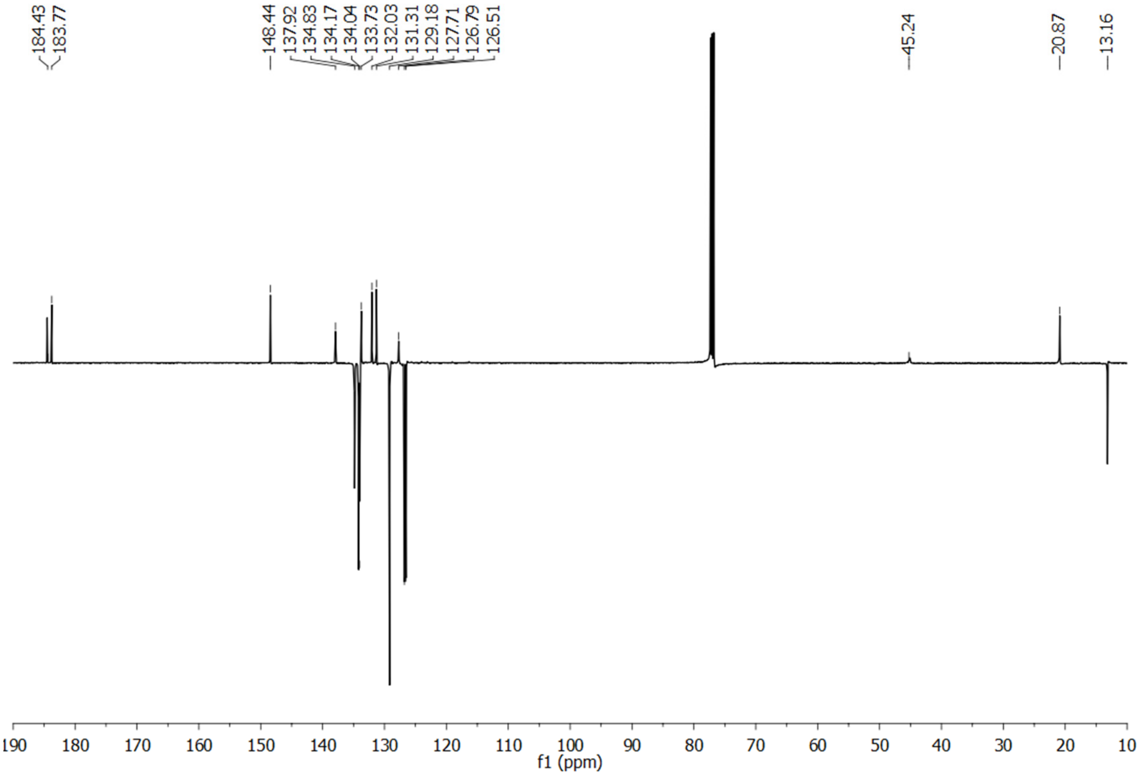

Figure S15. ^{13}C-APT NMR spectrum of compound **9b** in $CDCl_3$ at 75MHz.

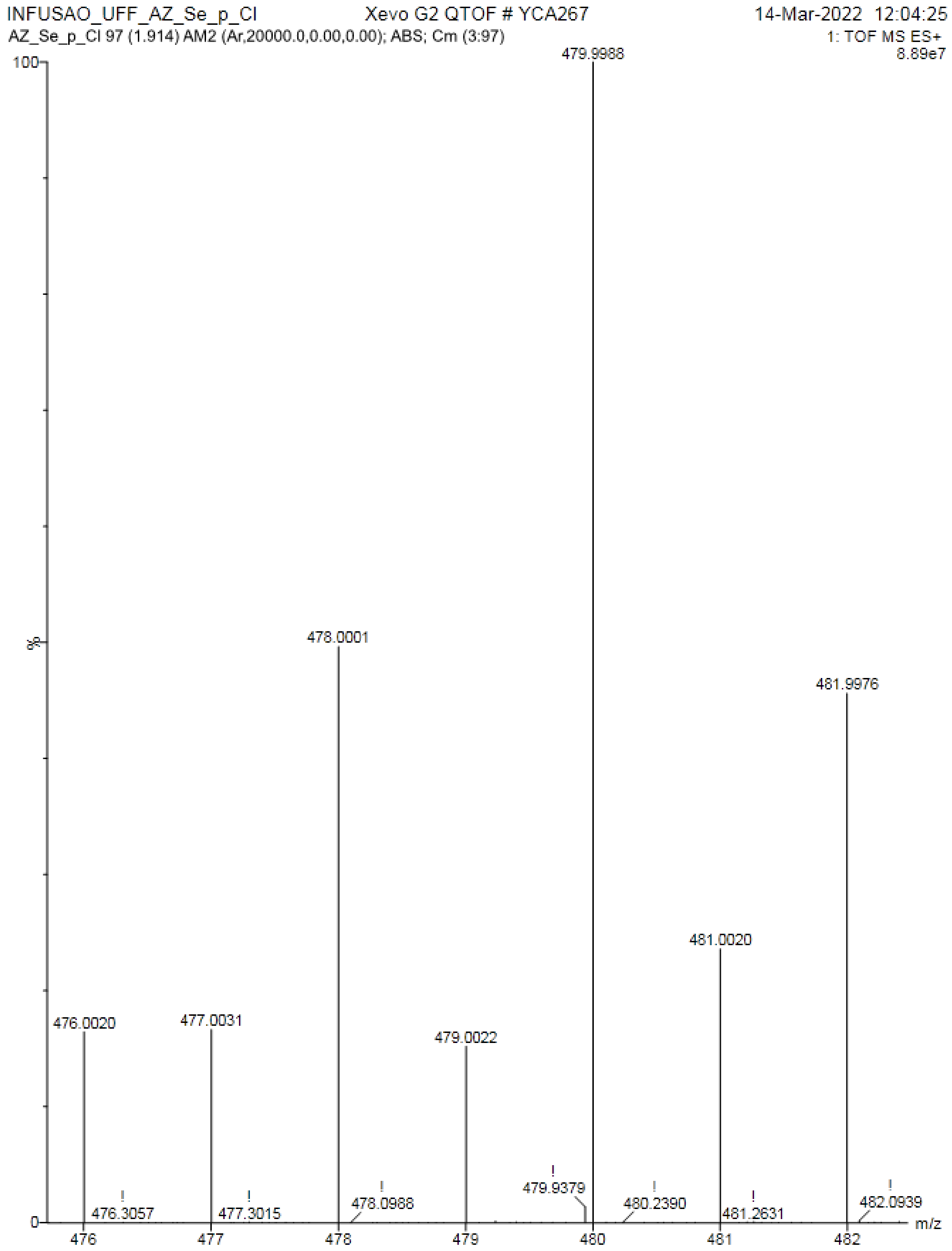

Figure S16. ESI MS spectrum of **9b**.

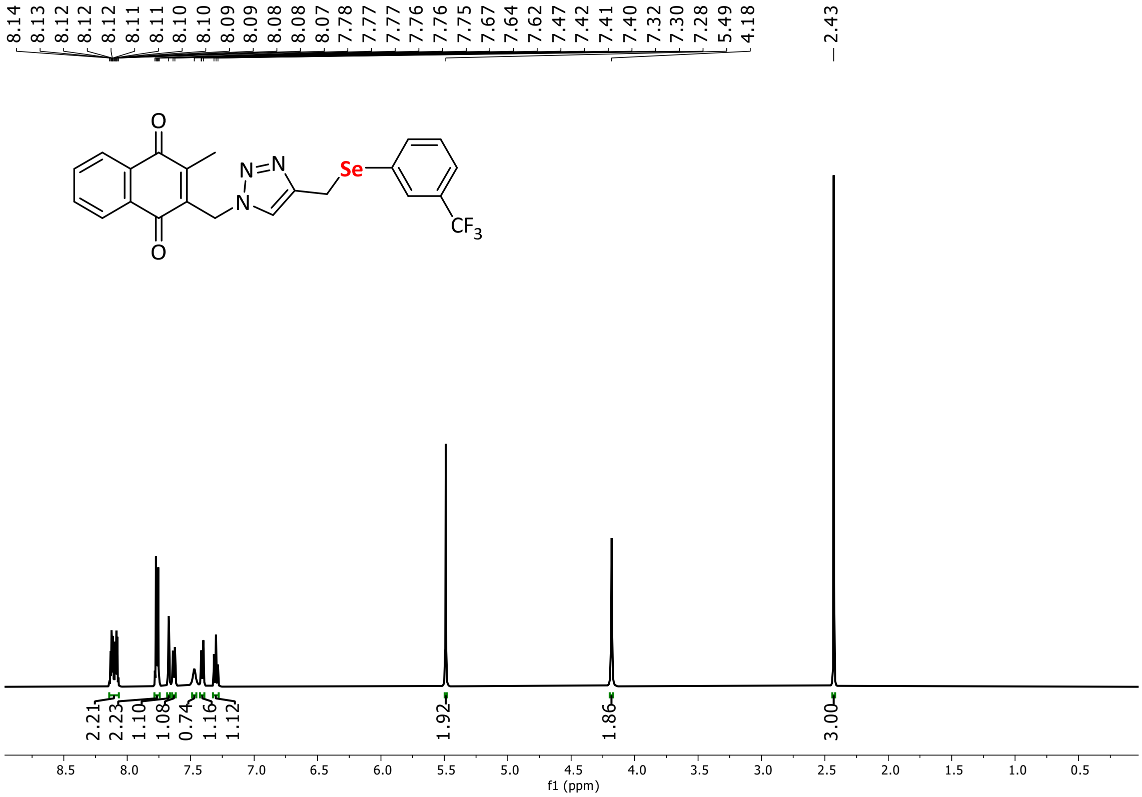

Figure S17. ^{1}H NMR spectrum of compound **9c** in $CDCl_3$ at 500MHz.

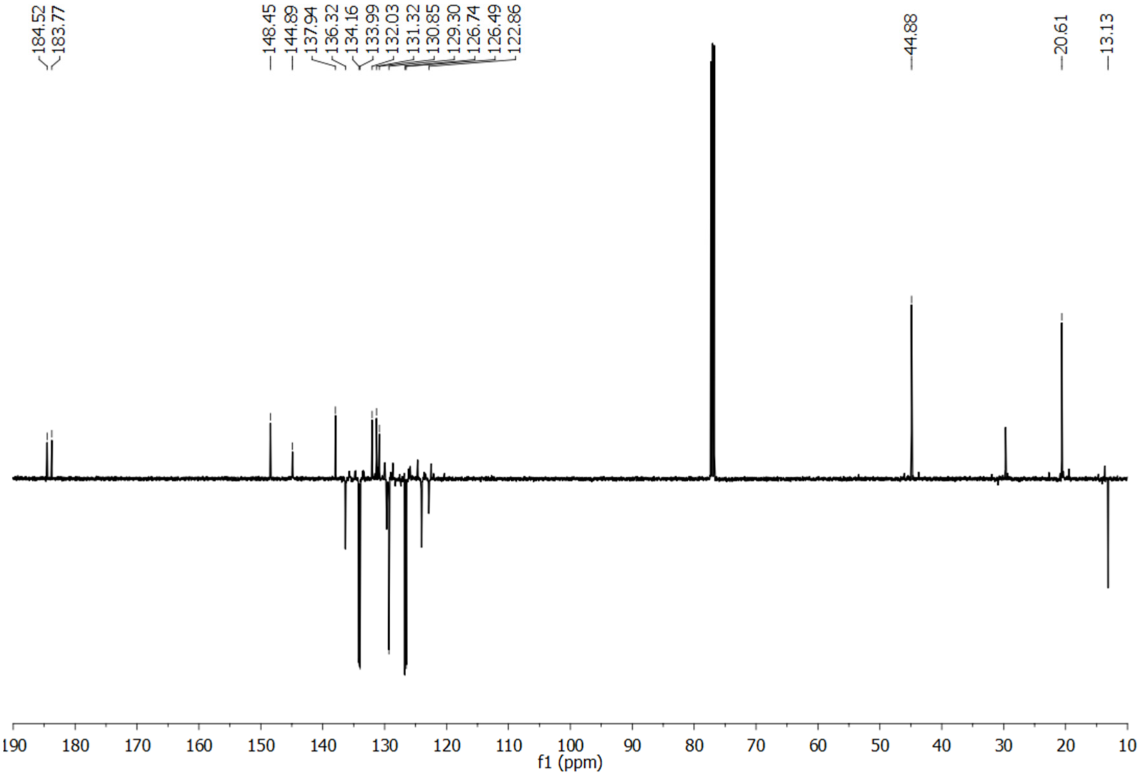

Figure S18. ^{13}C-APT NMR spectrum of compound **9c** in $CDCl_3$ at 75MHz.

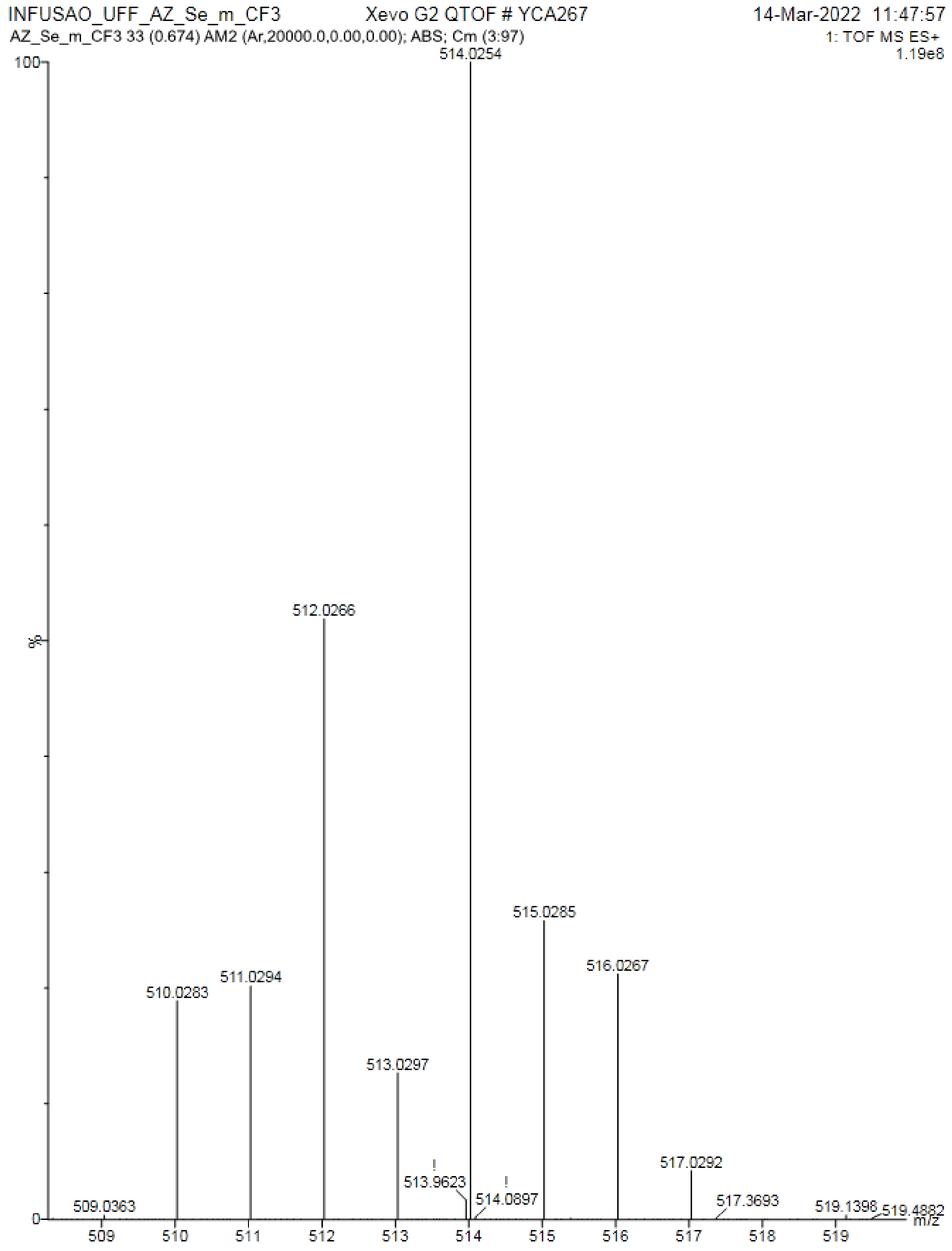

Figure S19. ESI MS spectrum of **9c**.

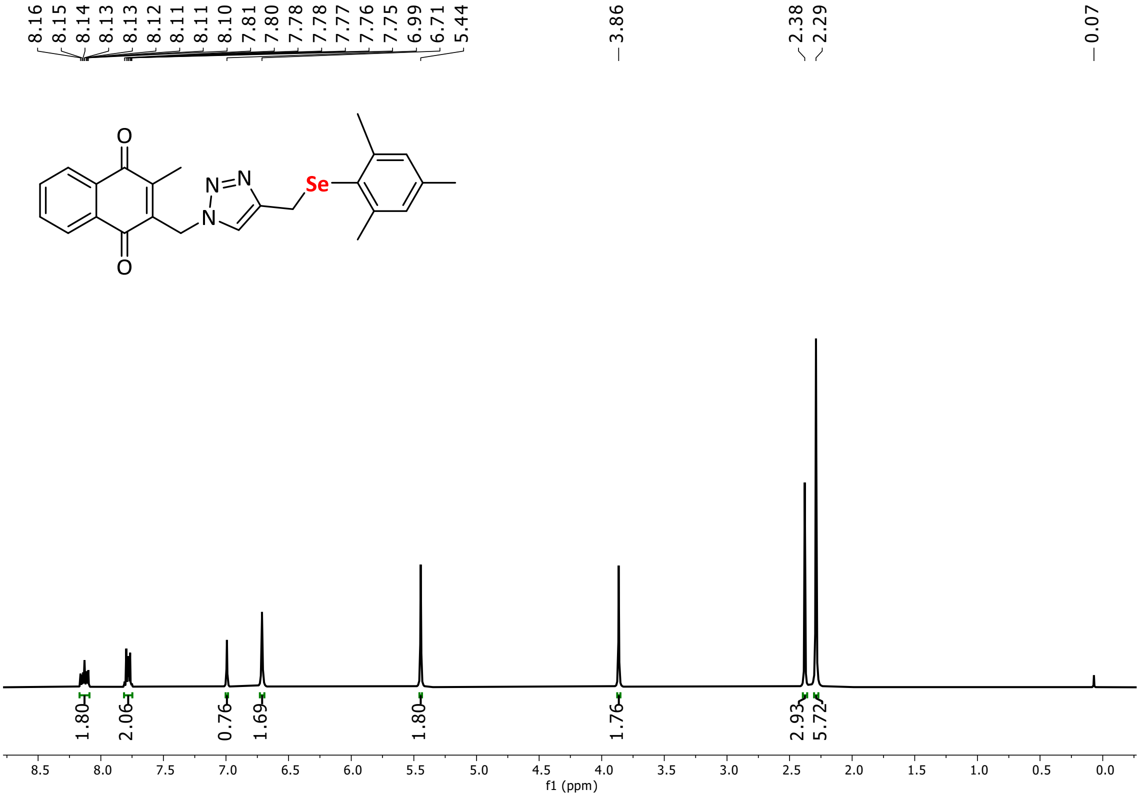

Figure S20. ^{1}H NMR spectrum of compound **9d** in $CDCl_3$ at 500MHz.

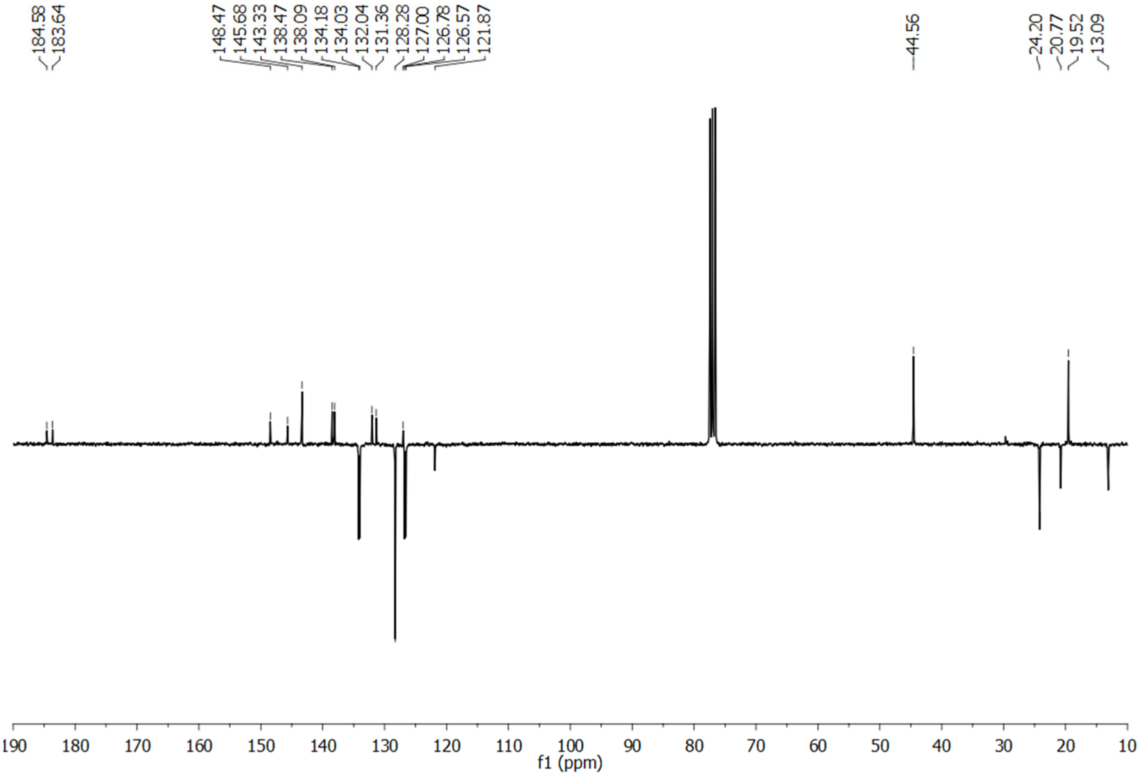

Figure S21. ^{13}C-APT NMR spectrum of compound **9d** in $CDCl_3$ at 75MHz.

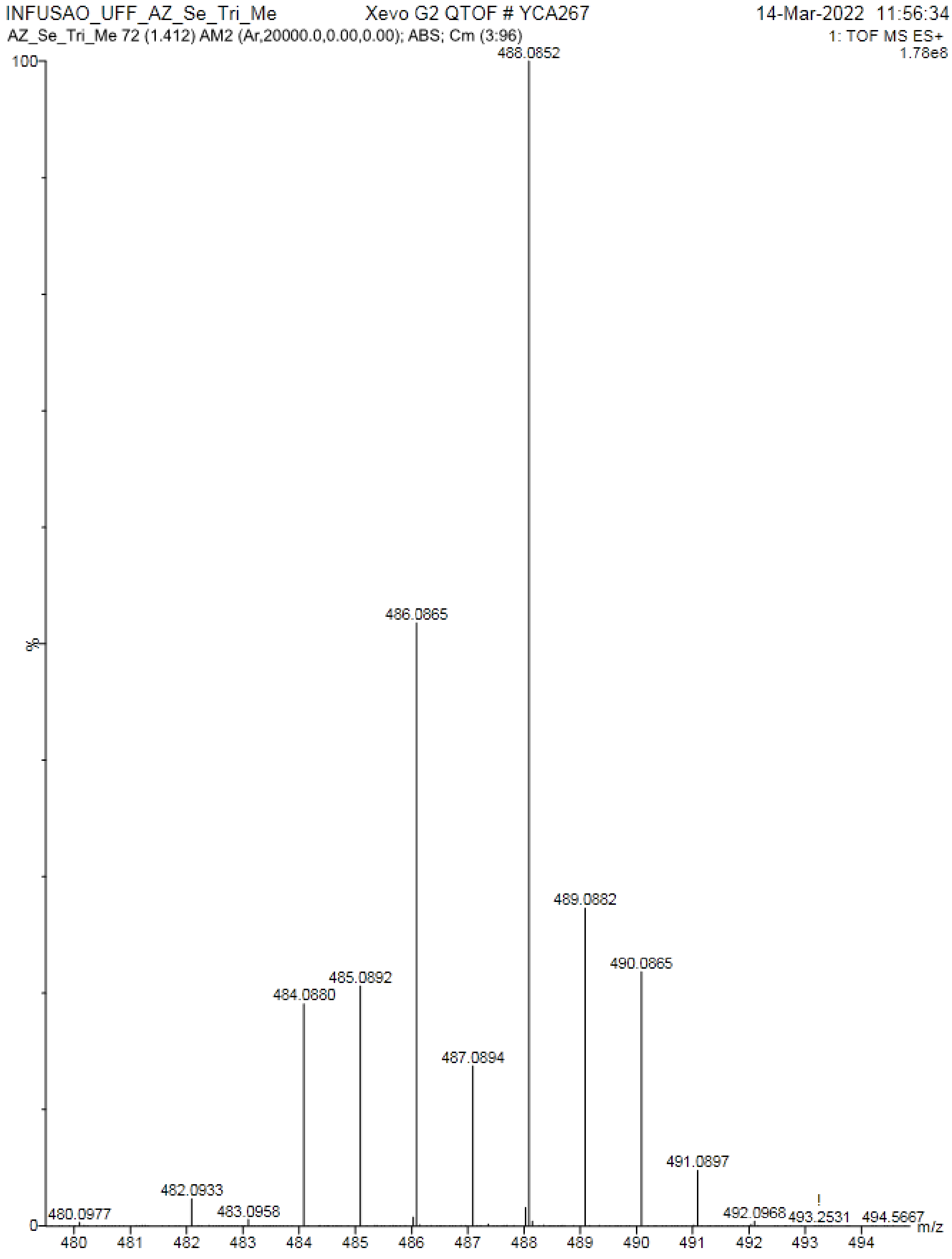

Figure S22. ESI MS spectrum of **9d**.

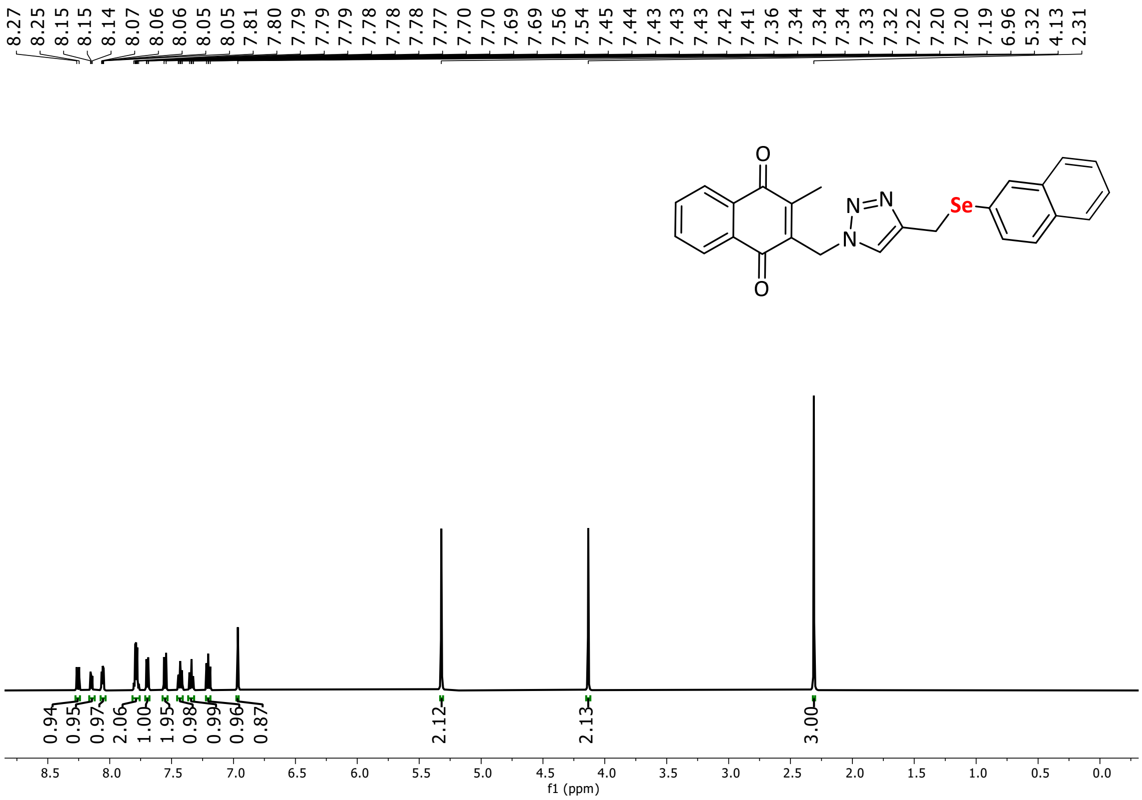

Figure S23. ^{1}H NMR spectrum of compound **9e** in $CDCl_3$ at 500MHz.

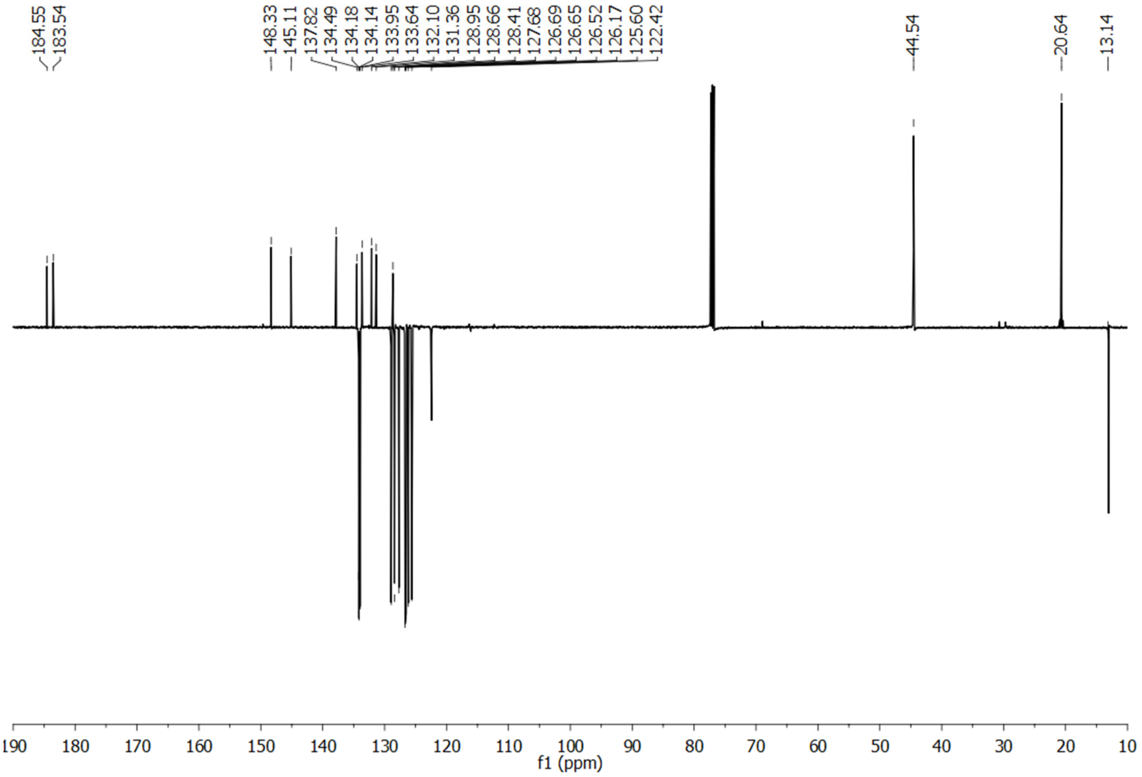

Figure S24. ^{13}C-APT NMR spectrum of compound **9e** in $CDCl_3$ at 75MHz.

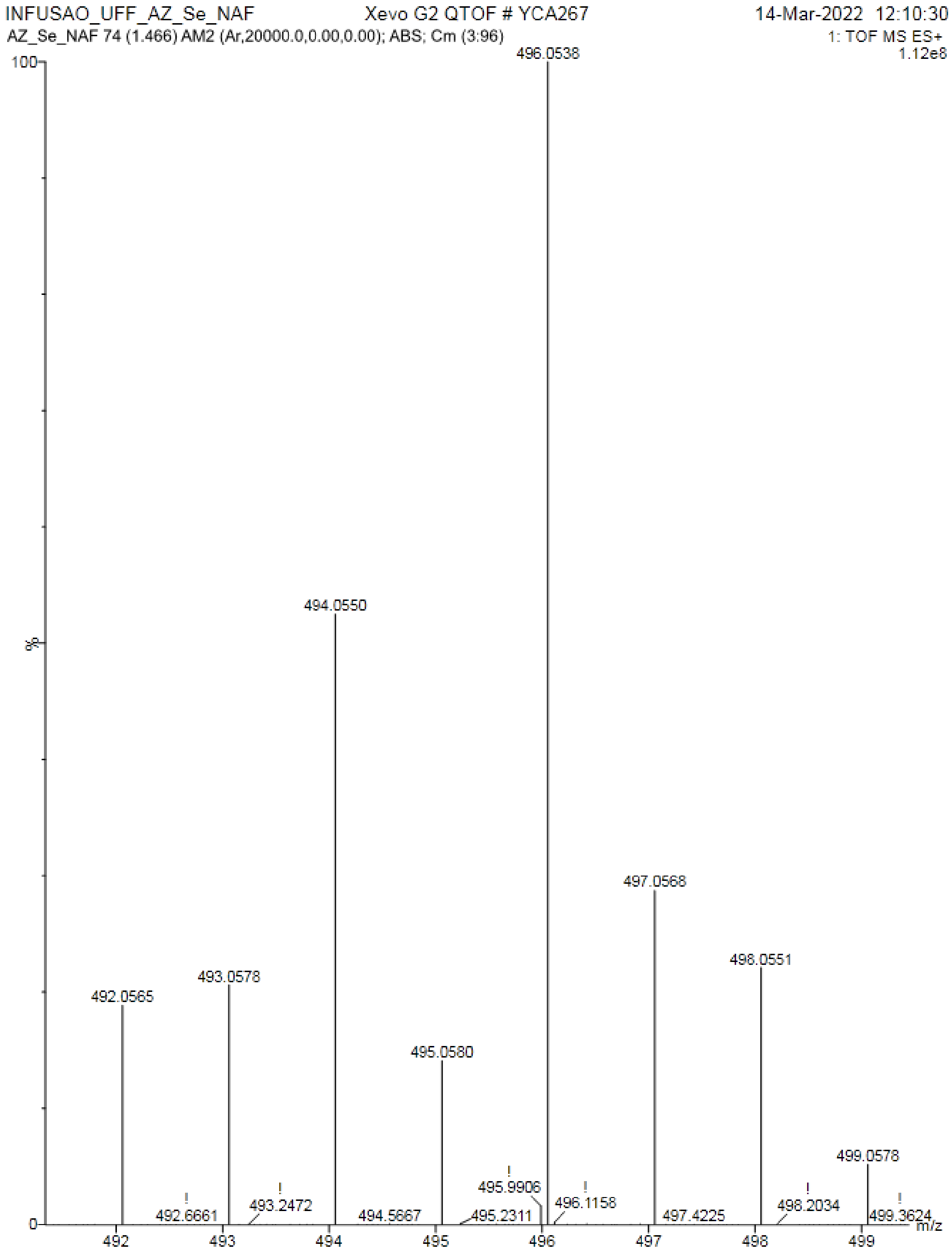

Figure S25. ESI MS spectrum of **9e**.

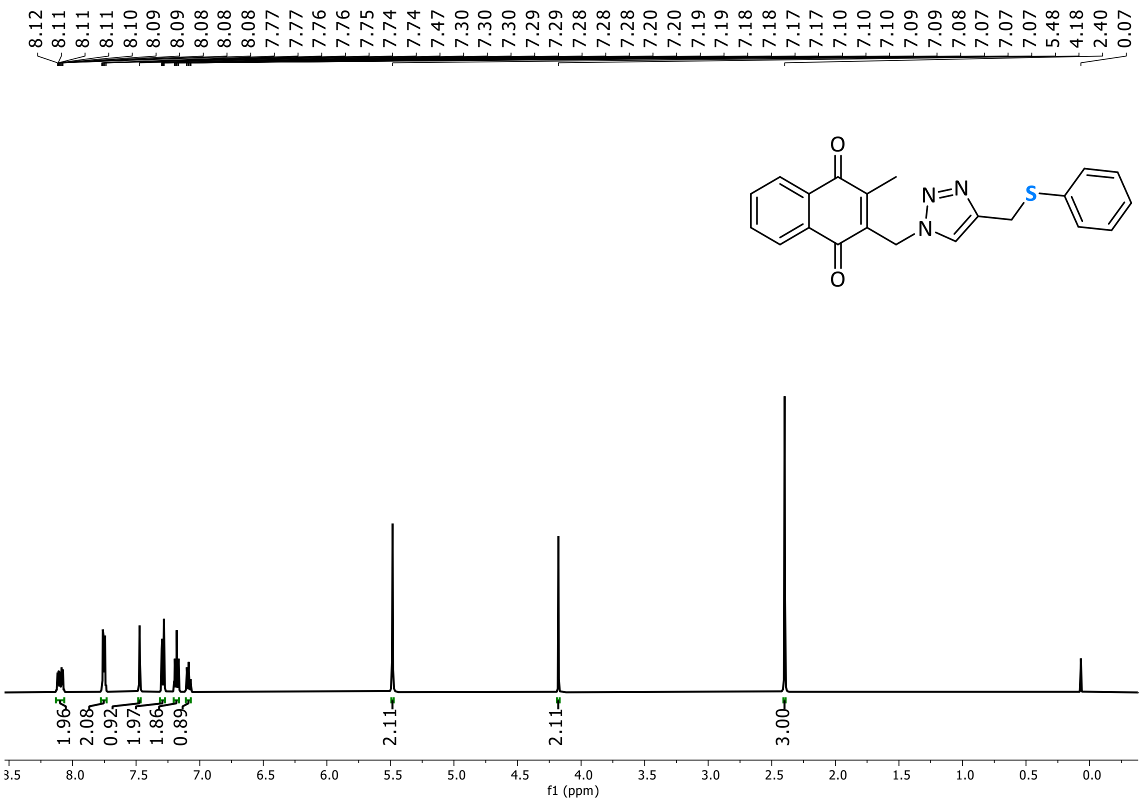

Figure S26. ^{1}H NMR spectrum of compound **10a** in $CDCl_3$ at 500MHz.

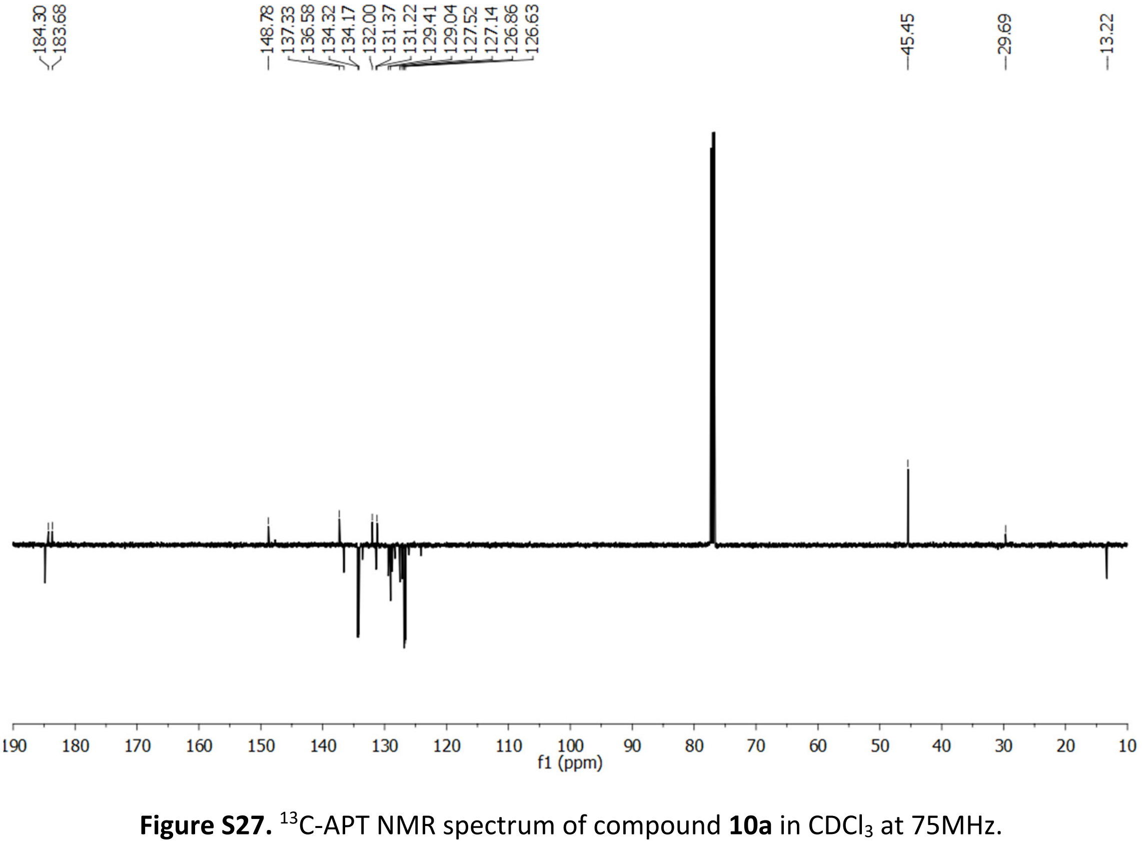

Figure S27. ^{13}C-APT NMR spectrum of compound **10a** in $CDCl_3$ at 75MHz.

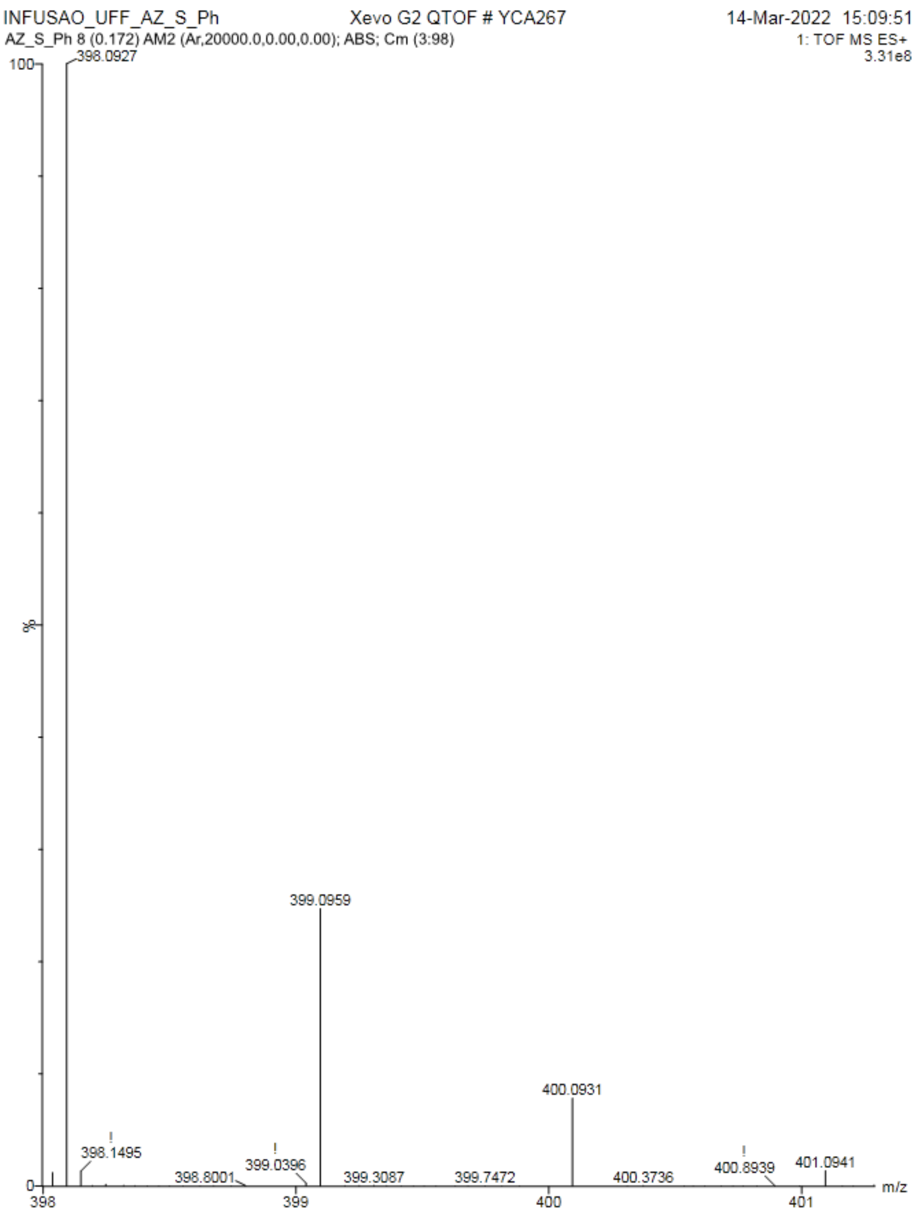

Figure S28. ESI MS spectrum of **10a**.

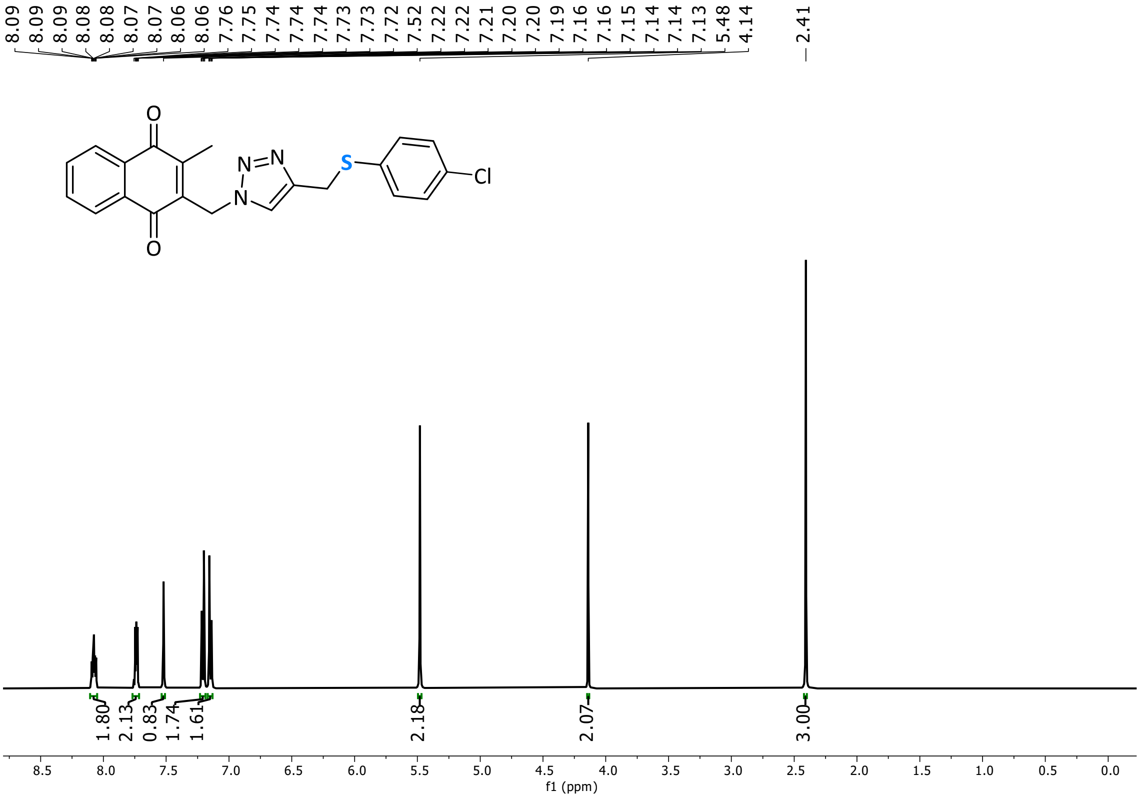

Figure S29. ^{1}H NMR spectrum of compound **10b** in $CDCl_3$ at 500MHz.

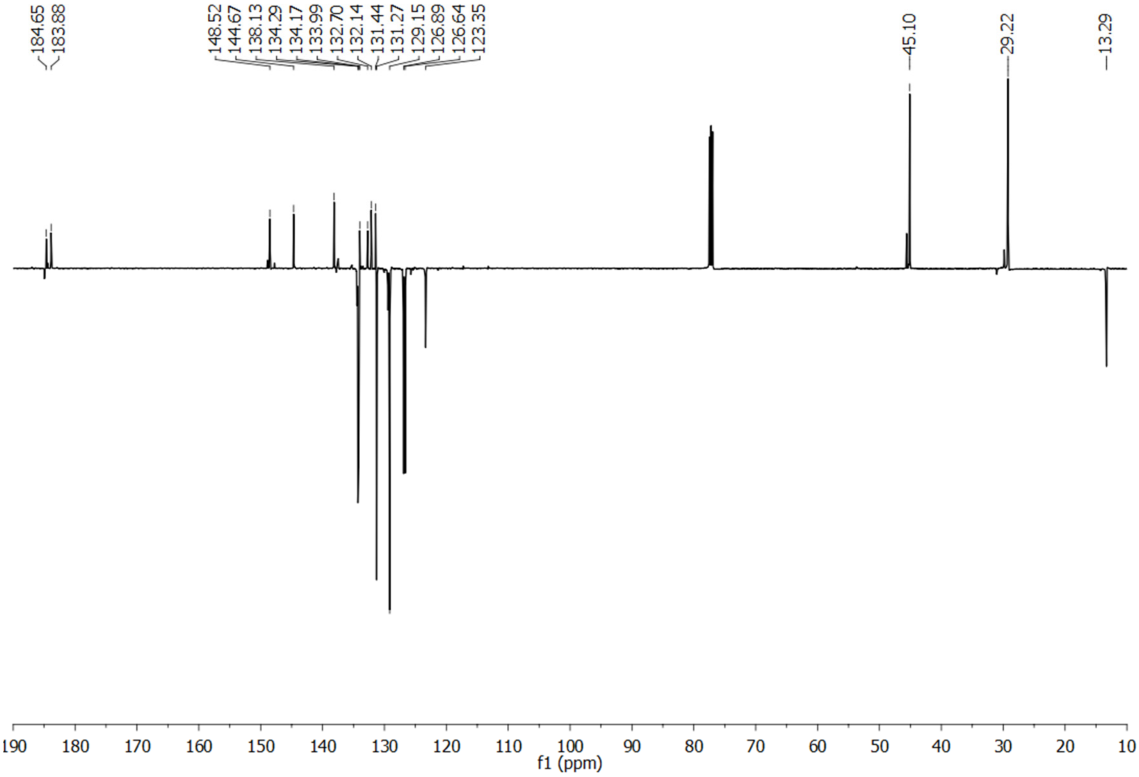

Figure S30. ^{13}C-APT NMR spectrum of compound **10b** in $CDCl_3$ at 75MHz.

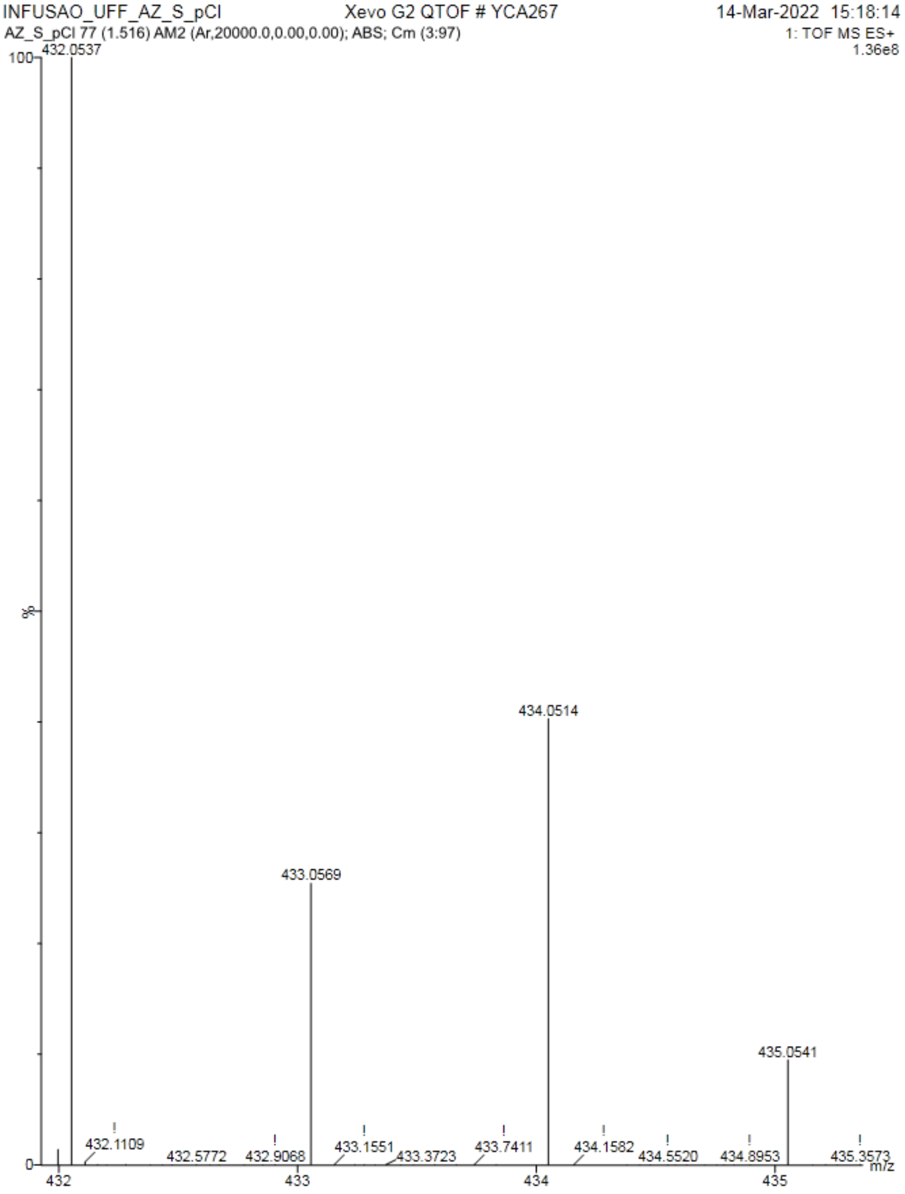

Figure S31. ESI MS spectrum of **10b**.

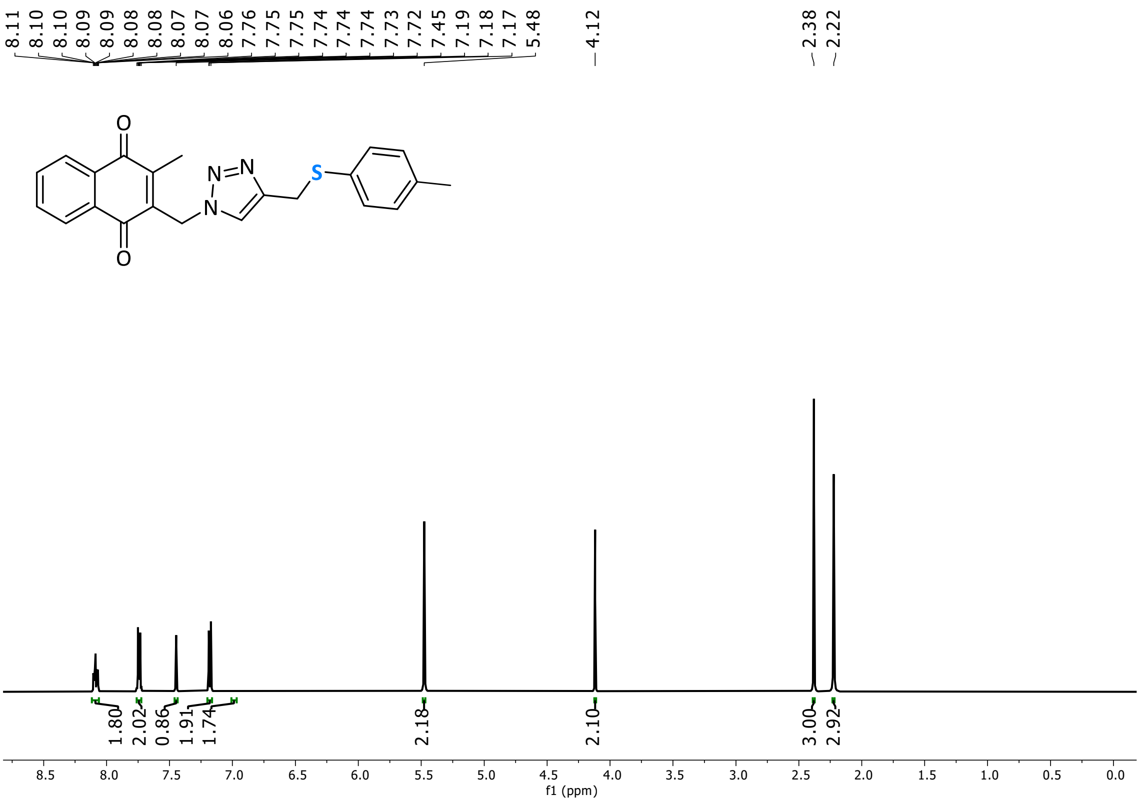

Figure S32. ^{1}H NMR spectrum of compound **10c** in $CDCl_3$ at 500MHz.

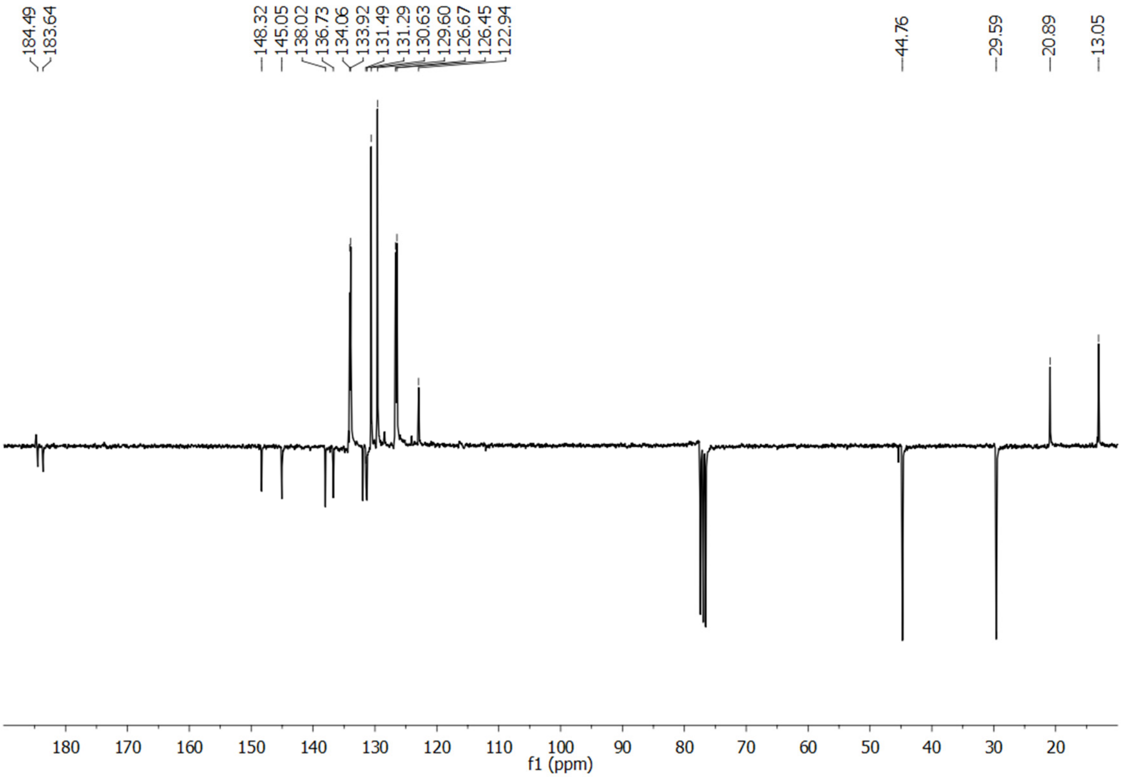

Figure S33. ^{13}C-APT NMR spectrum of compound **10c** in $CDCl_3$ at 75MHz.

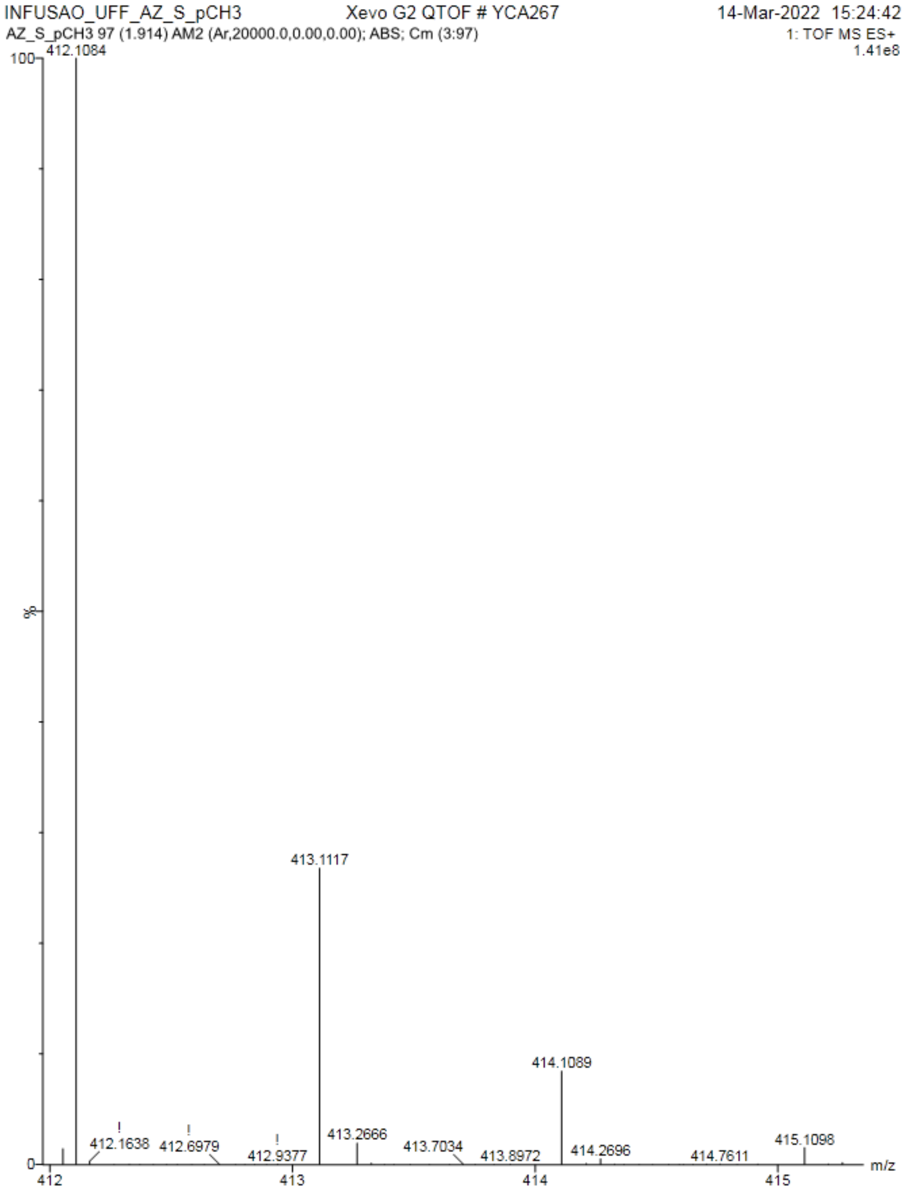

Figure S34. ESI MS spectrum of **10c**.

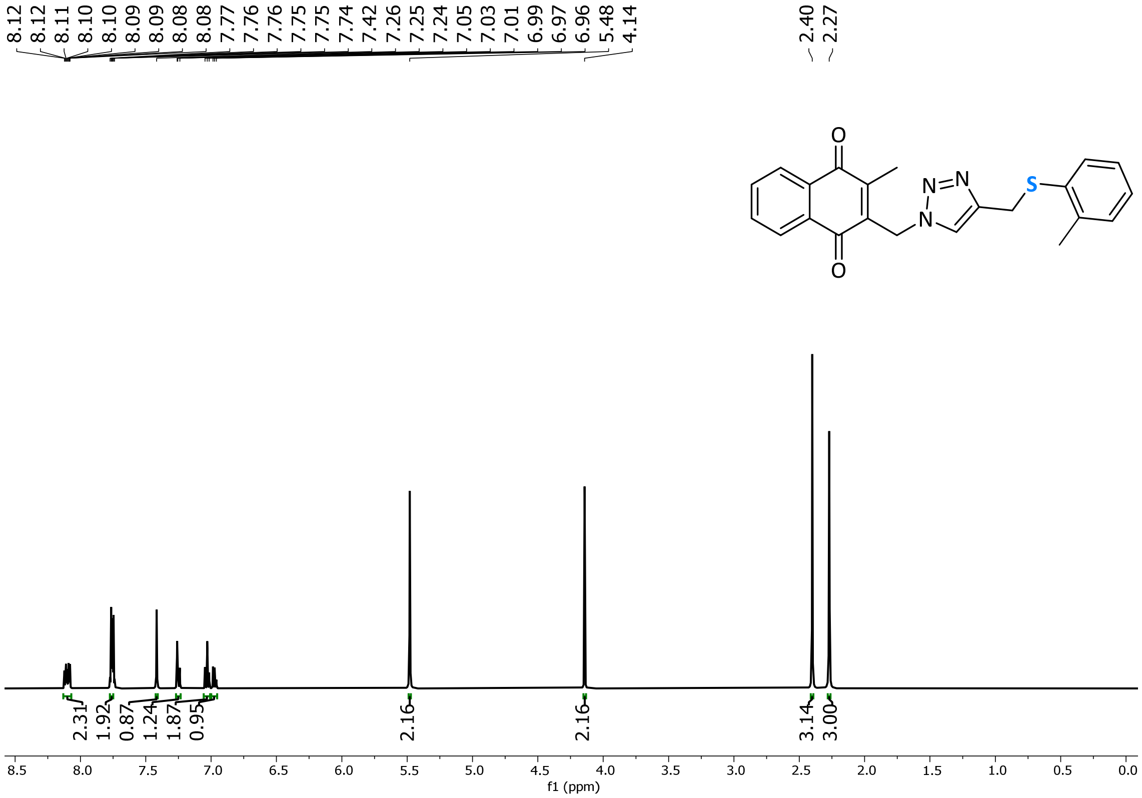

Figure S35. ^{1}H NMR spectrum of compound **10d** in $CDCl_3$ at 500MHz.

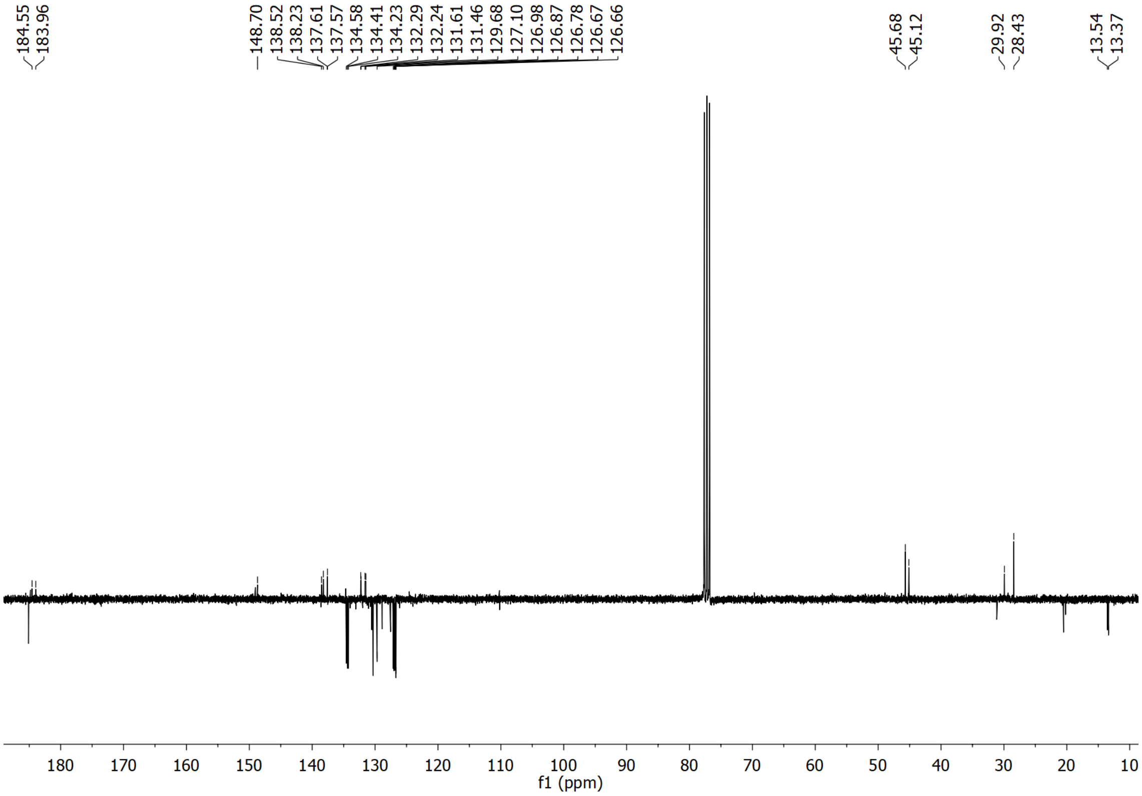

Figure S36. ^{13}C-APT NMR spectrum of compound **10d** in $CDCl_3$ at 75MHz.

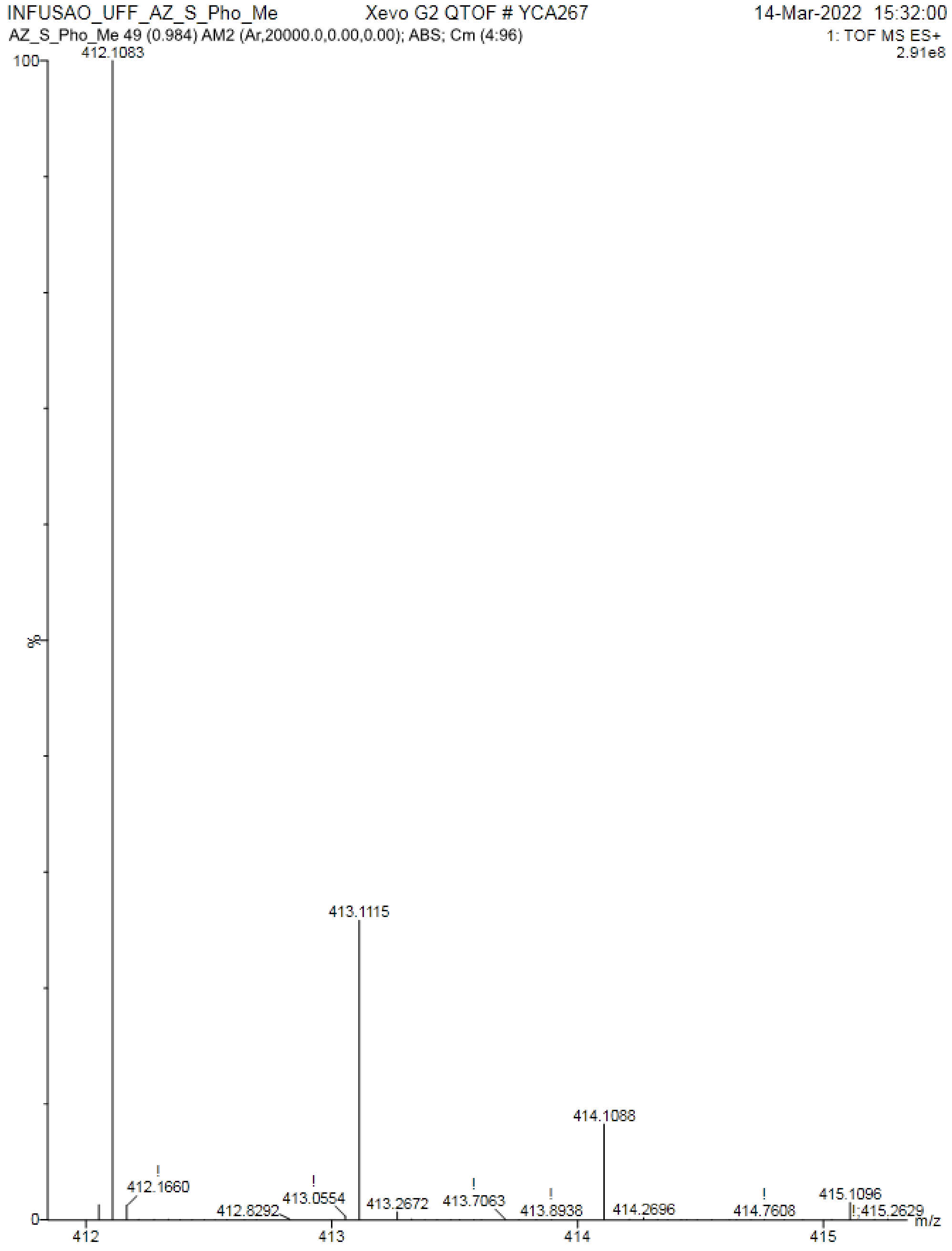

Figure S37. ESI MS spectrum of **10d**.

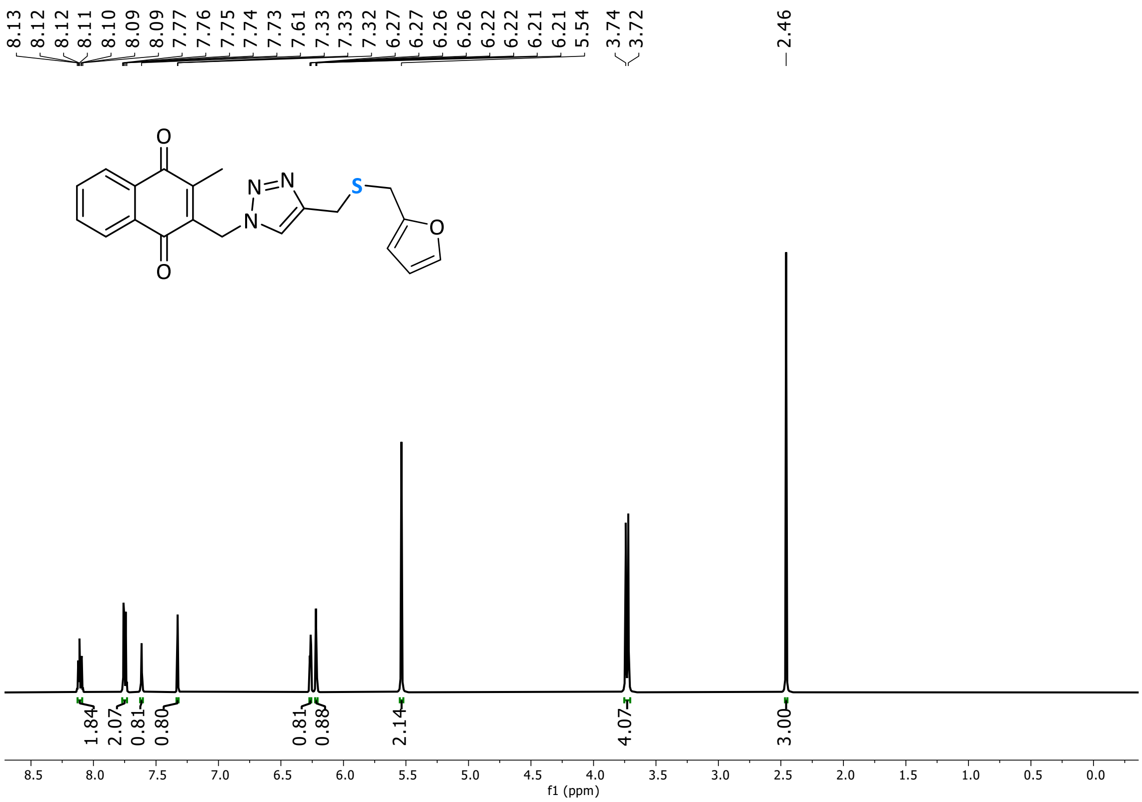

Figure S38. ^{1}H NMR spectrum of compound **10e** in $CDCl_3$ at 500MHz.

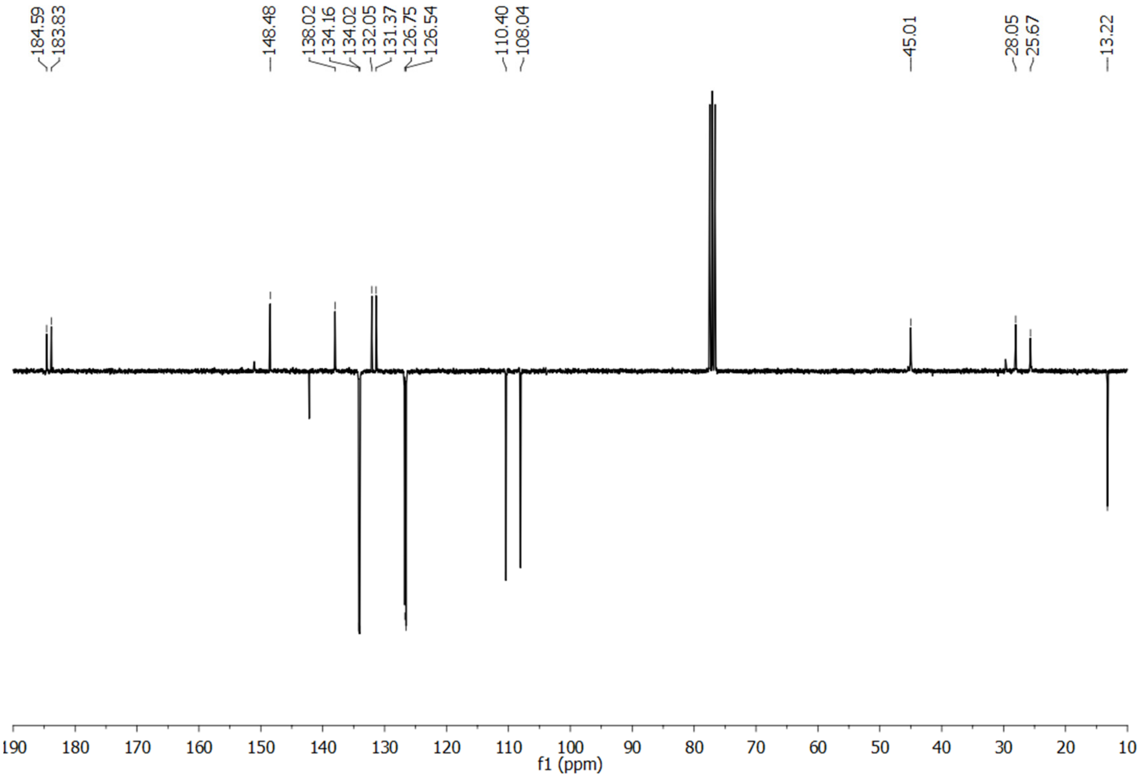

Figure S39. ^{13}C-APT NMR spectrum of compound **10e** in $CDCl_3$ at 75MHz.

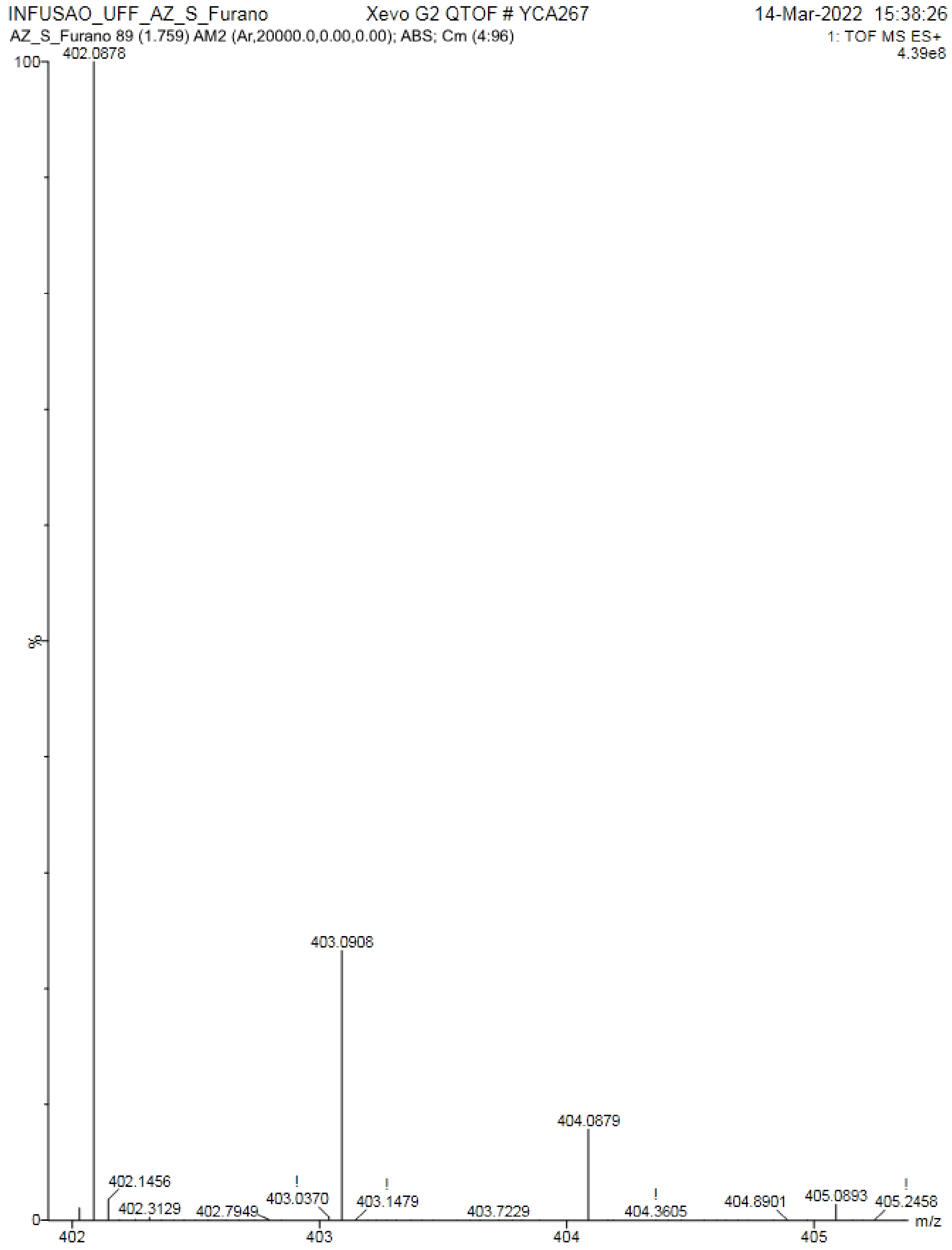

Figure S40. ESI MS spectrum of **10e**.

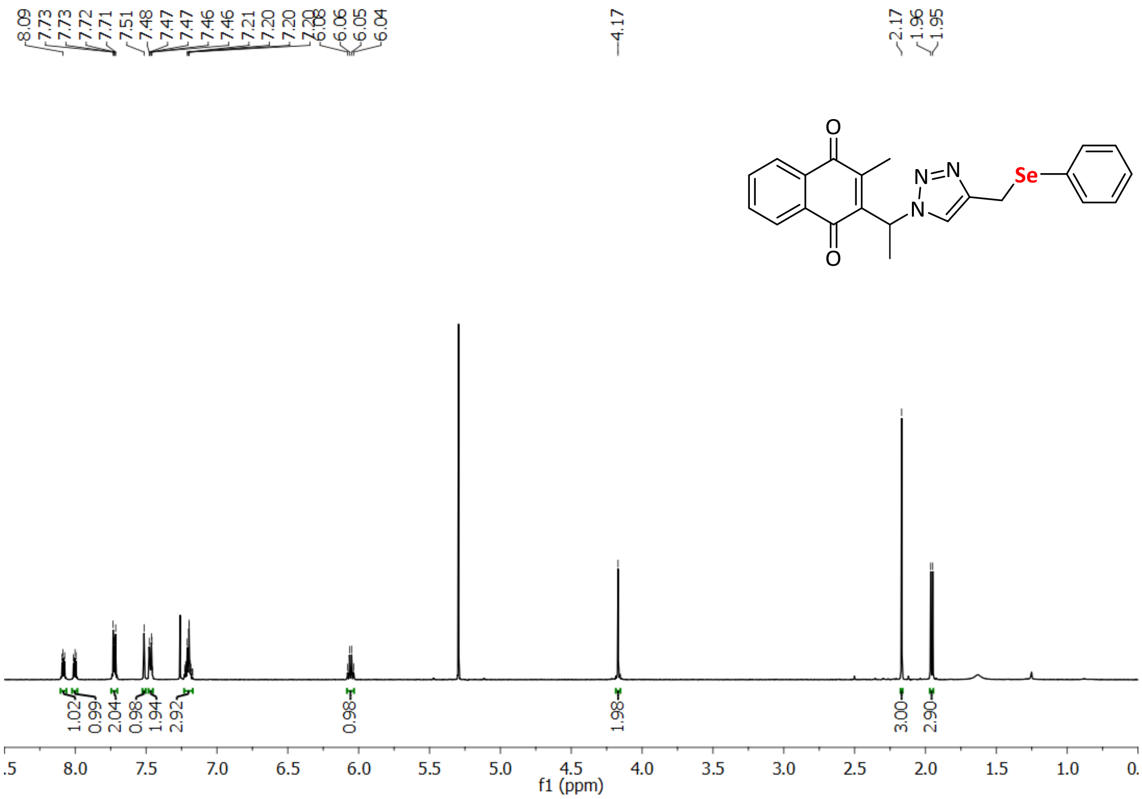

Figure S41. ^{1}H NMR spectrum of compound **9f** in $CDCl_3$ at 500MHz.

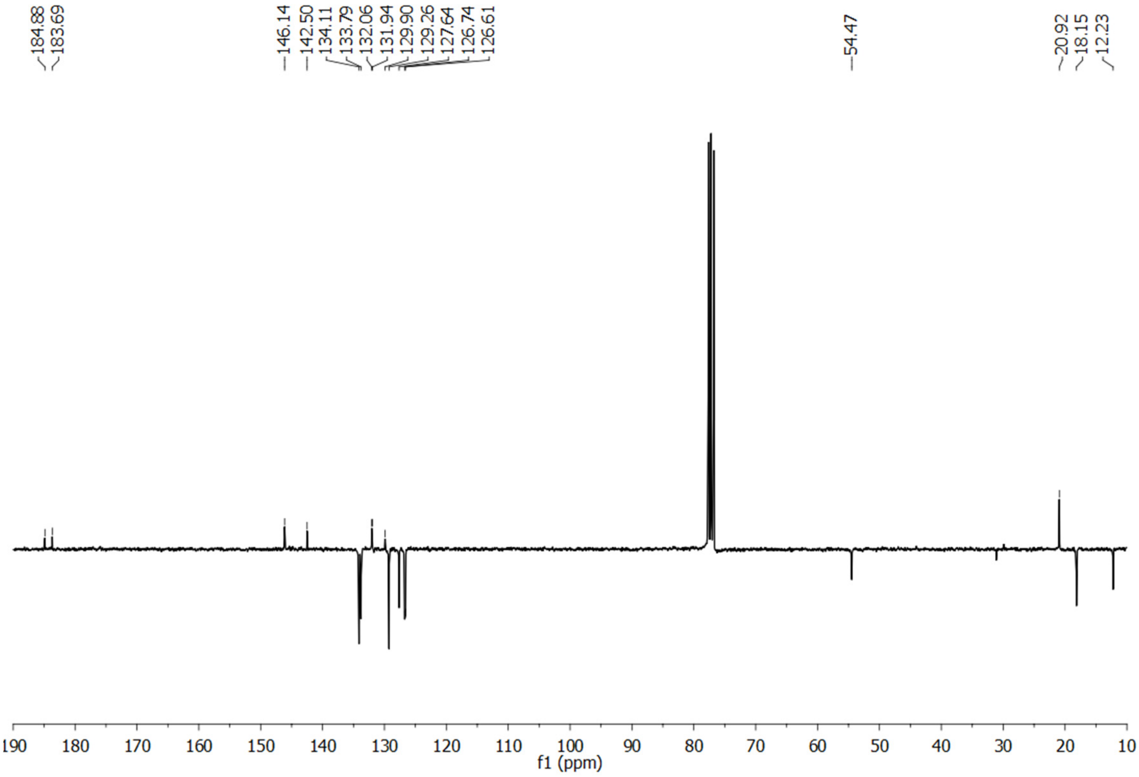

Figure S42. ^{13}C-APT NMR spectrum of compound **9f** in $CDCl_3$ at 75MHz.

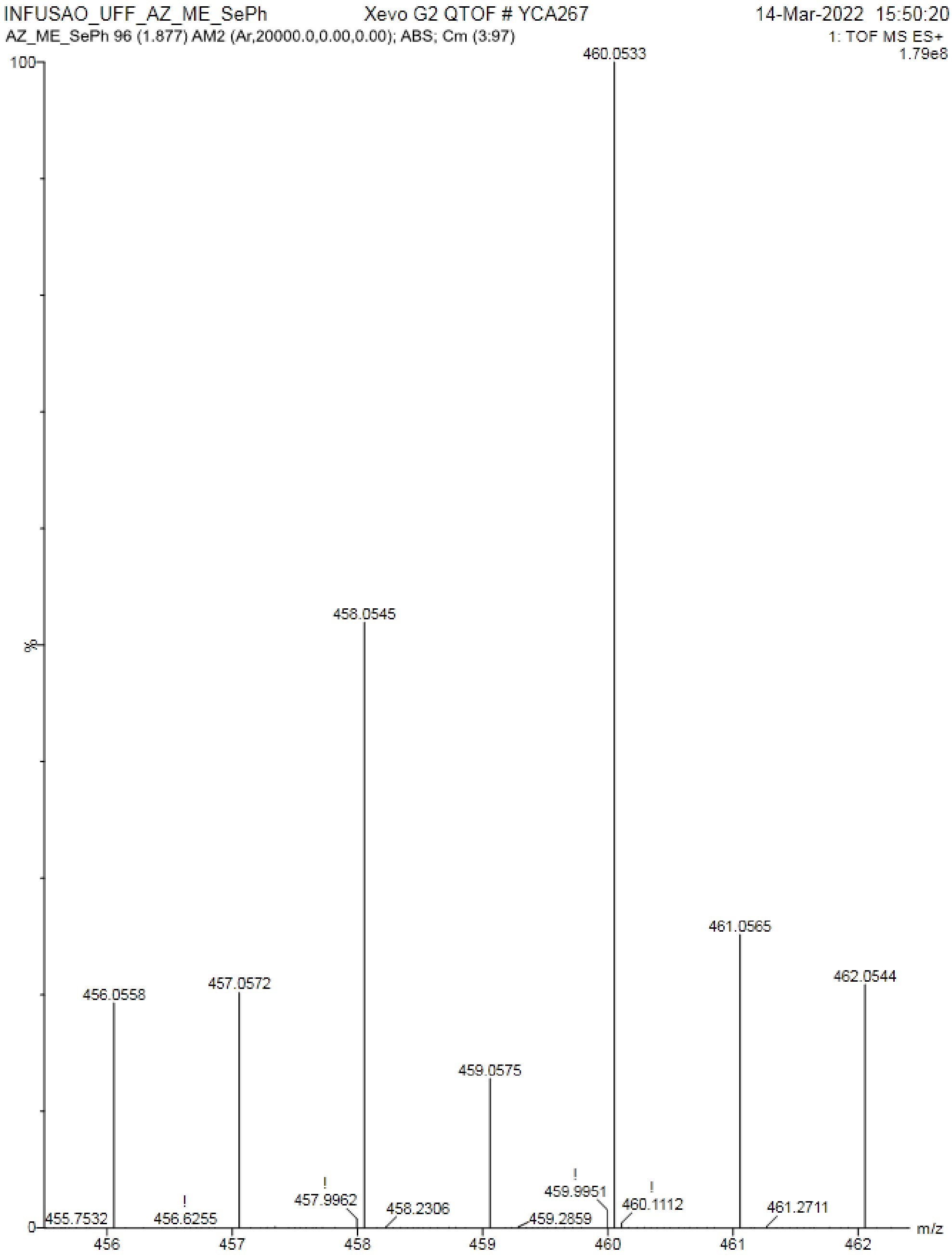

Figure S43. ESI MS spectrum of **9f**.

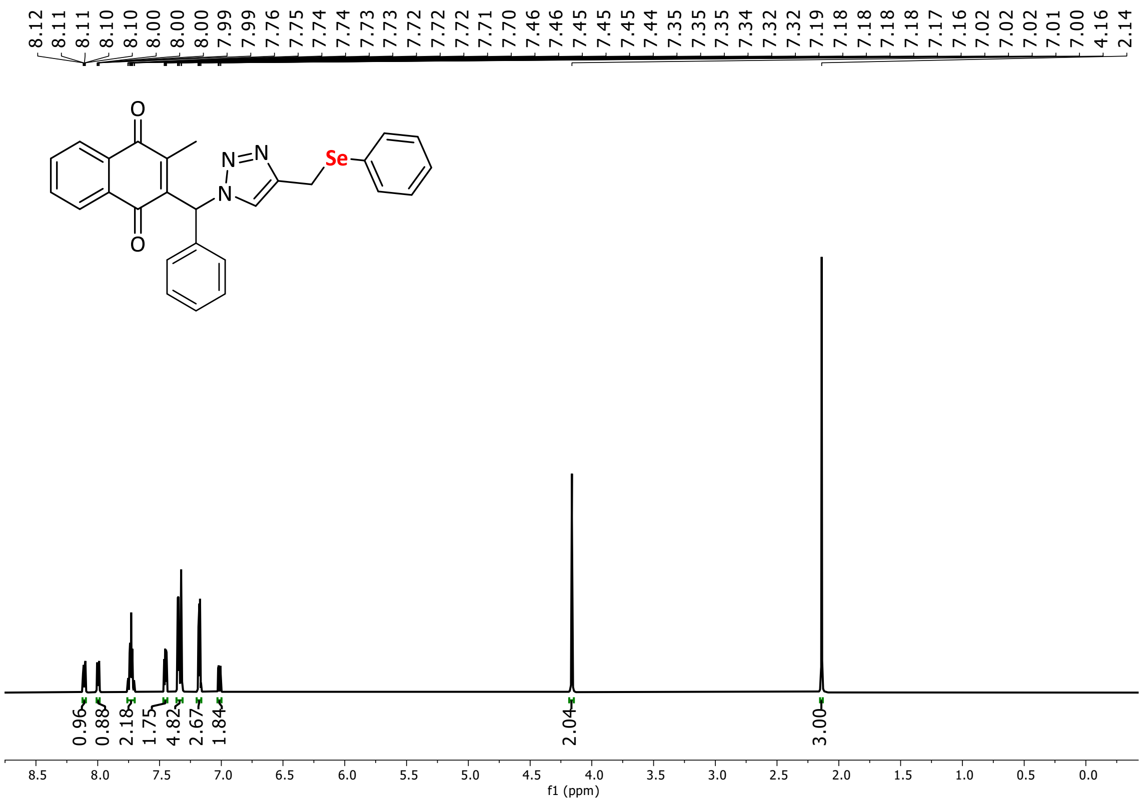

Figure S44. ^{1}H NMR spectrum of compound **9g** in $CDCl_3$ at 500MHz.

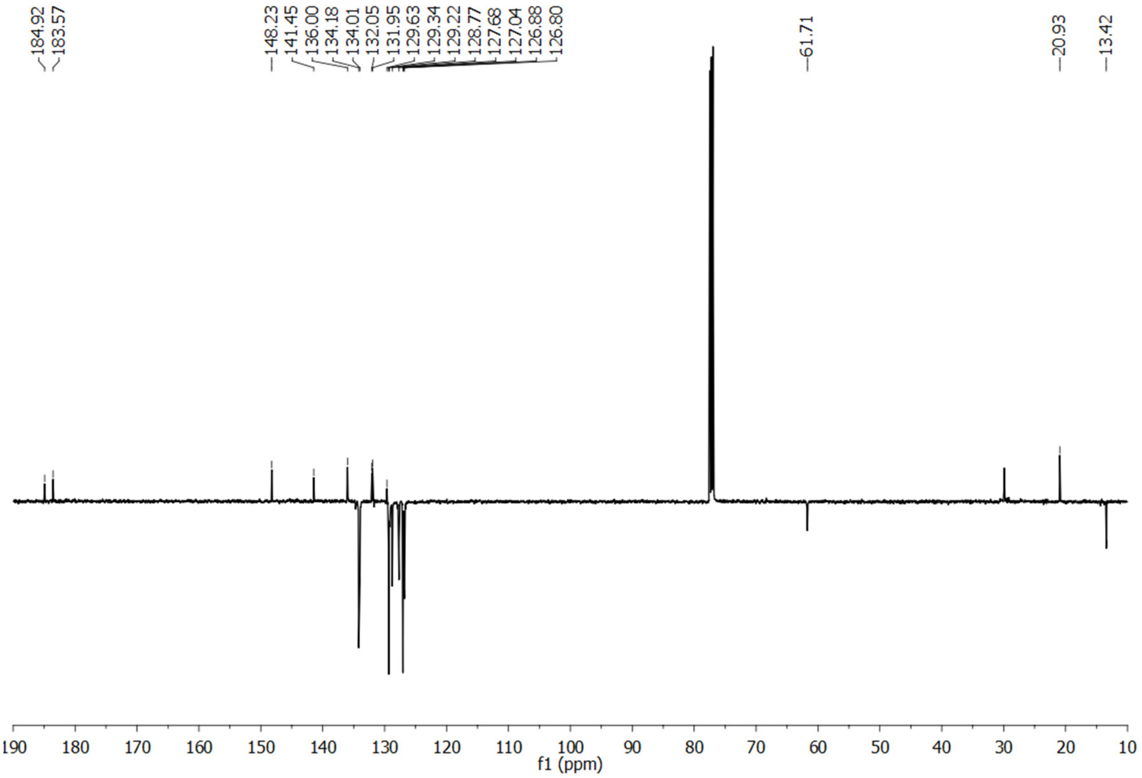

Figure S45. ^{13}C-APT NMR spectrum of compound **9g** in $CDCl_3$ at 75MHz.

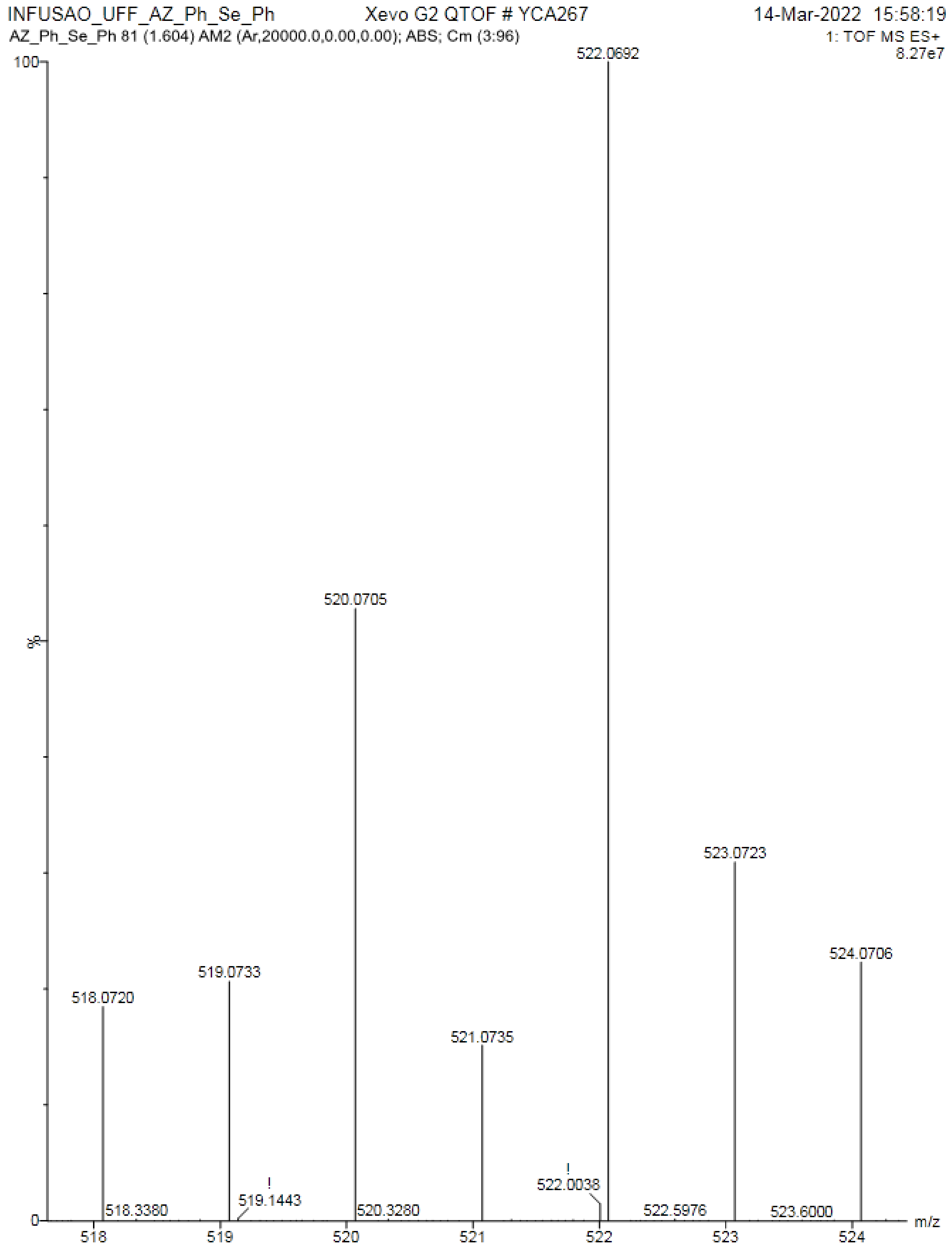

Figure S46. ESI MS spectrum of **9g**.

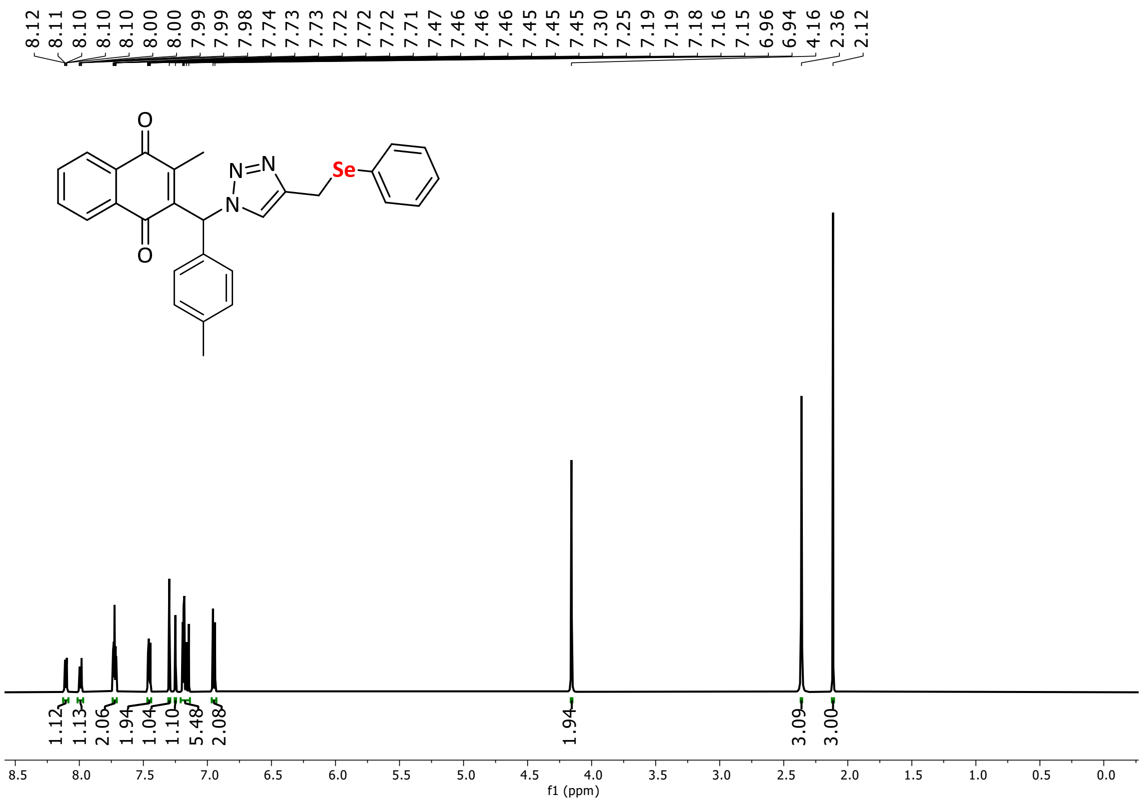

Figure S47. ^{1}H NMR spectrum of compound **9h** in $CDCl_3$ at 500MHz.

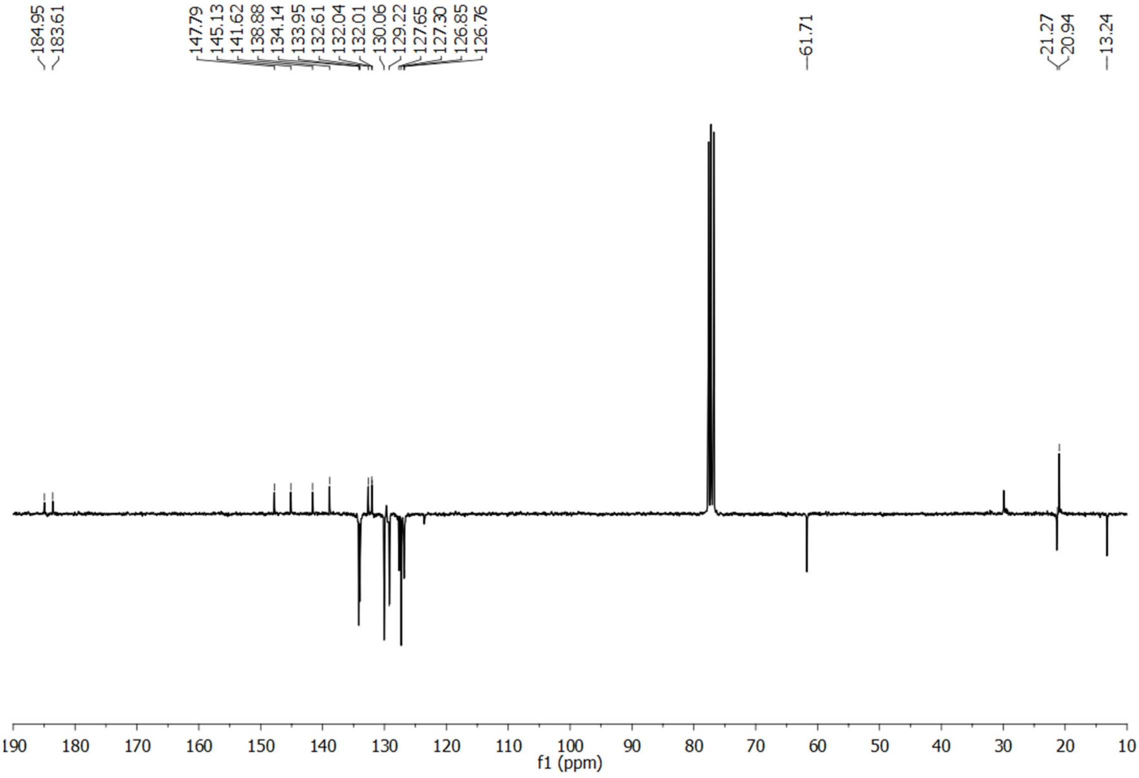

Figure S48. ^{13}C-APT NMR spectrum of compound **9h** in $CDCl_3$ at 75MHz.

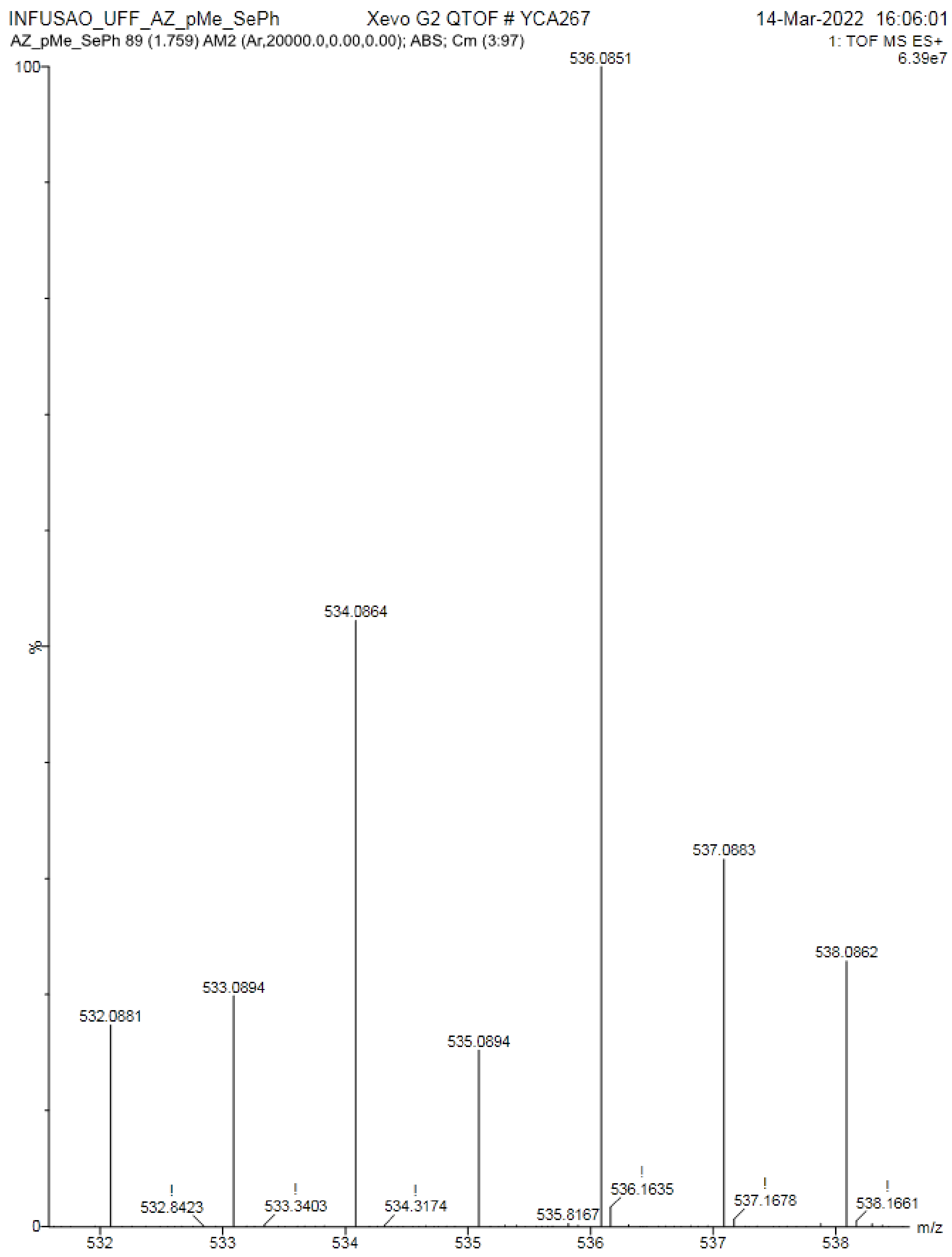

Figure S49. ESI MS spectrum of **9h**.

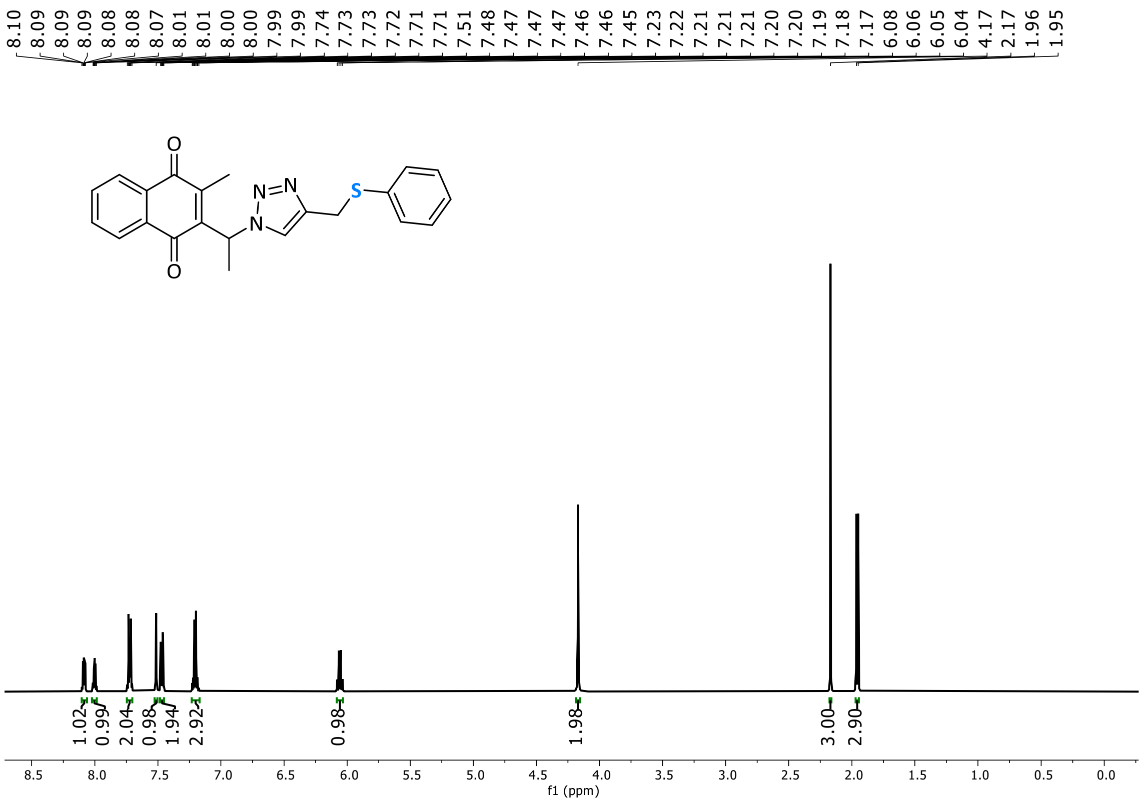

Figure S50. ^{1}H NMR spectrum of compound **10f** in $CDCl_3$ at 500MHz.

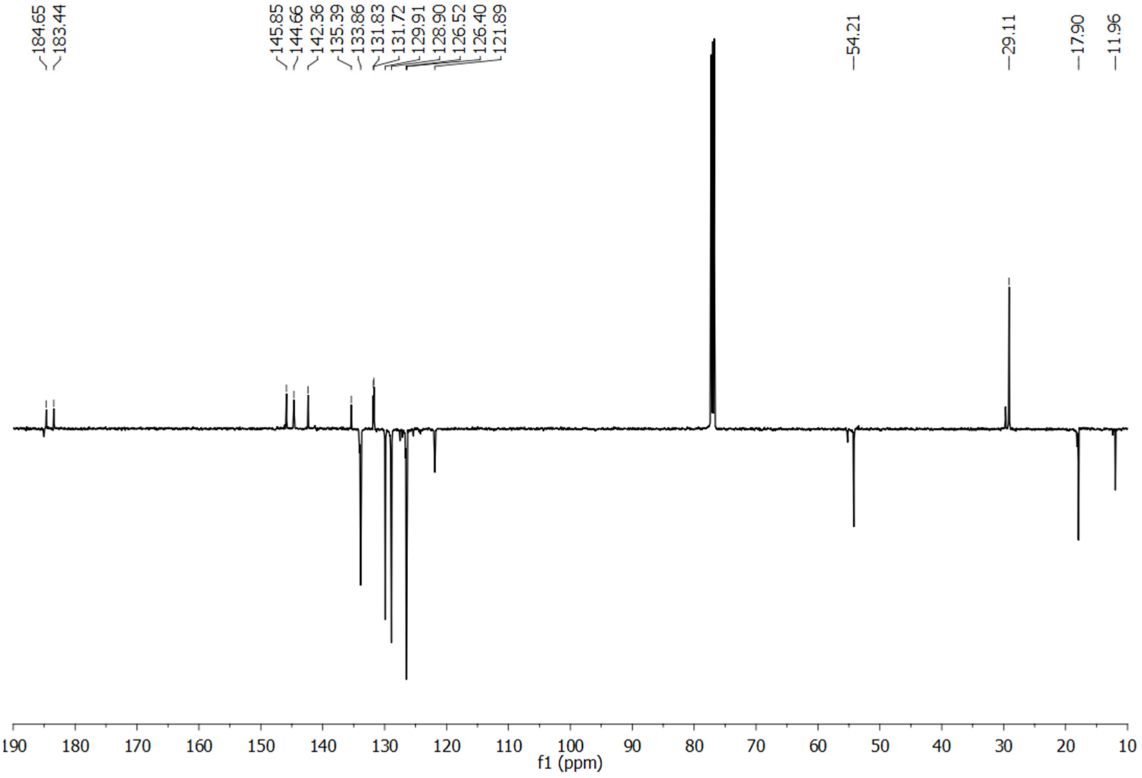

Figure S51. ^{13}C-APT NMR spectrum of compound **10f** in $CDCl_3$ at 75MHz.

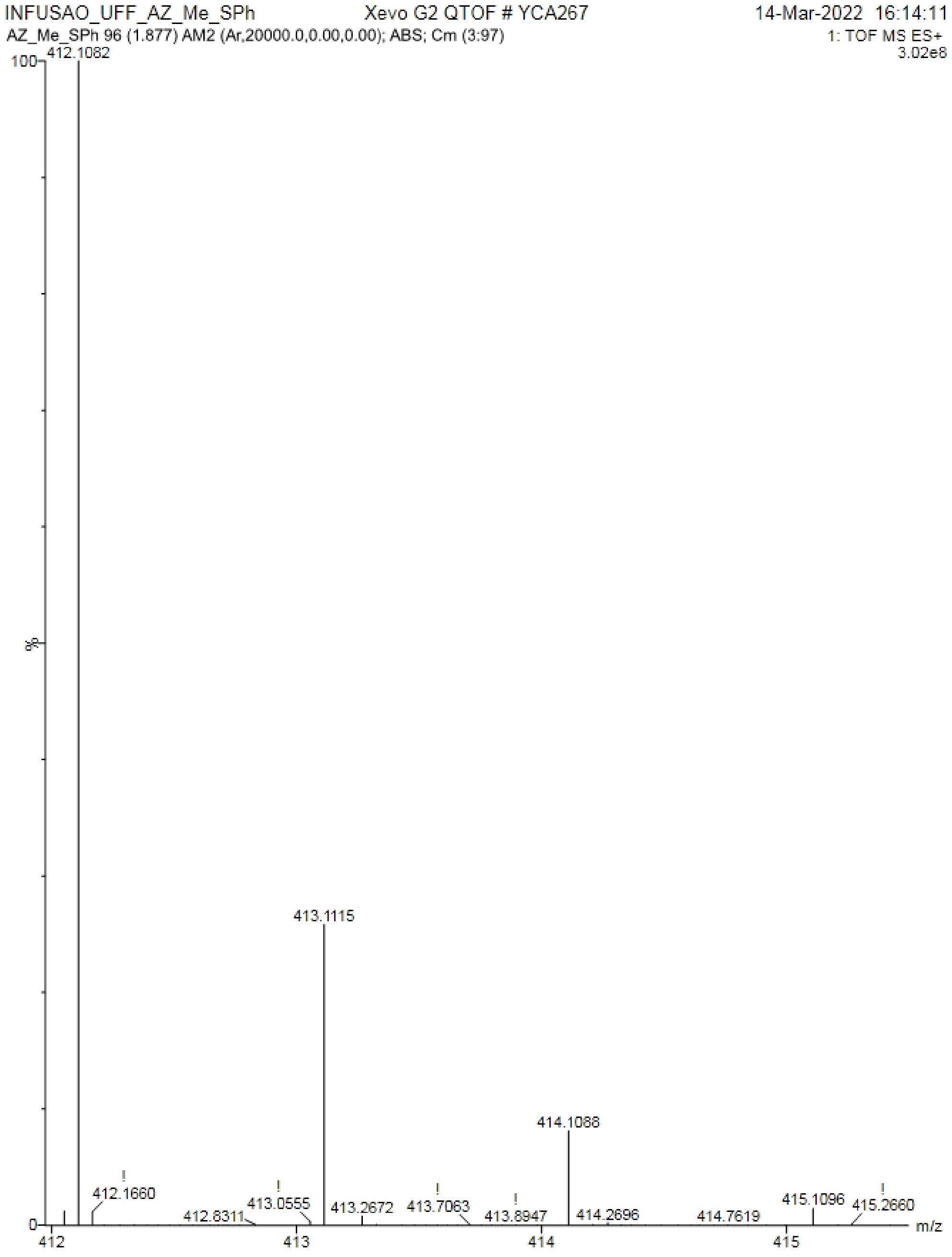

Figure S52. ESI MS spectrum of **10f**.

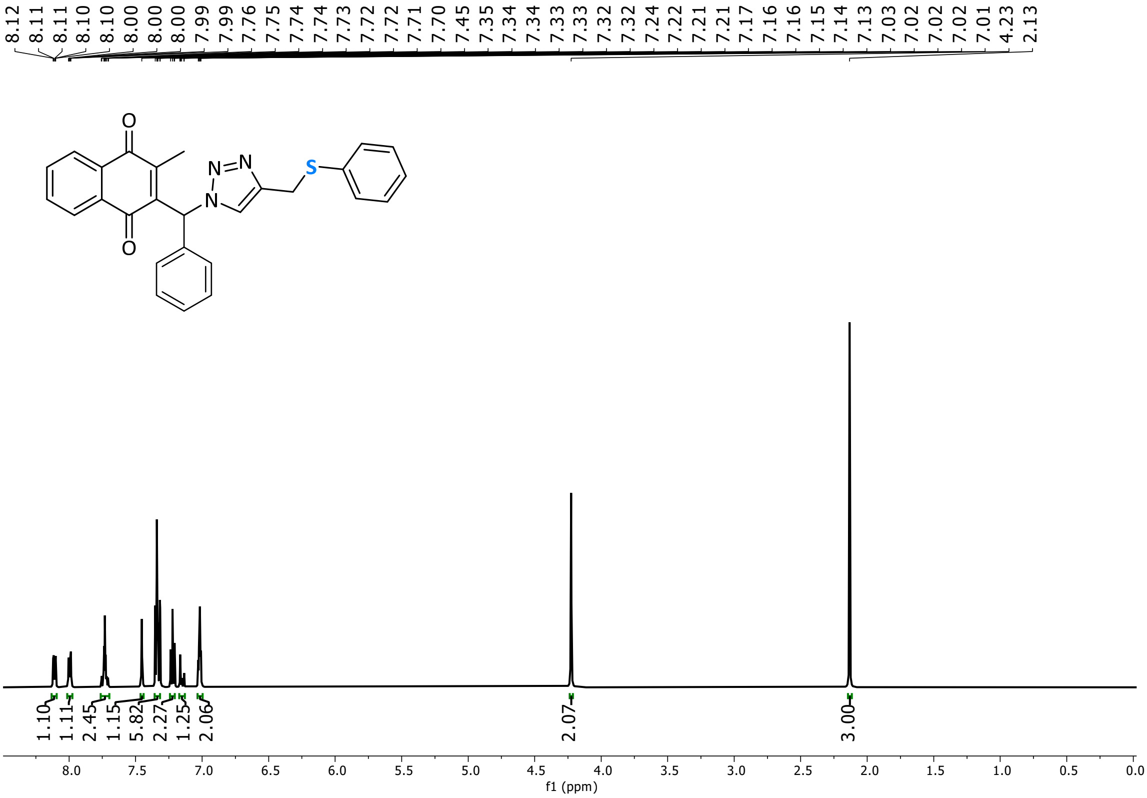

Figure S53. ^{1}H NMR spectrum of compound **10g** in $CDCl_3$ at 500MHz.

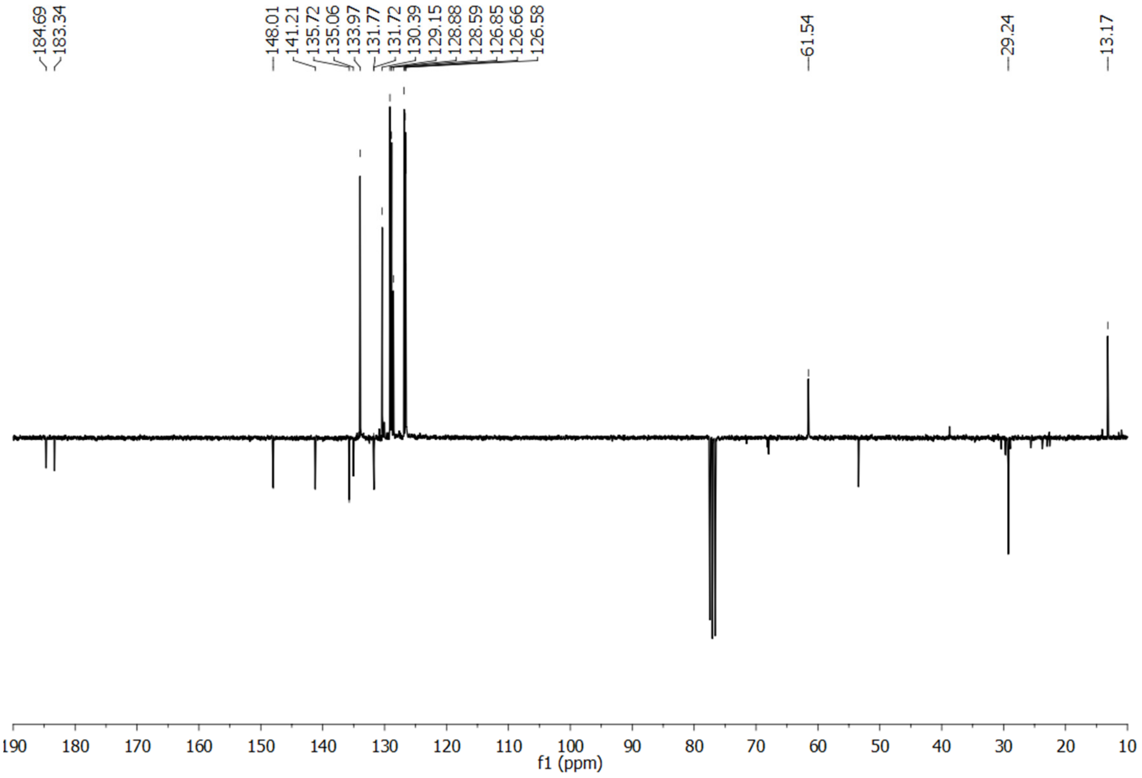

Figure S54. ^{13}C-APT NMR spectrum of compound **10g** in $CDCl_3$ at 75MHz.

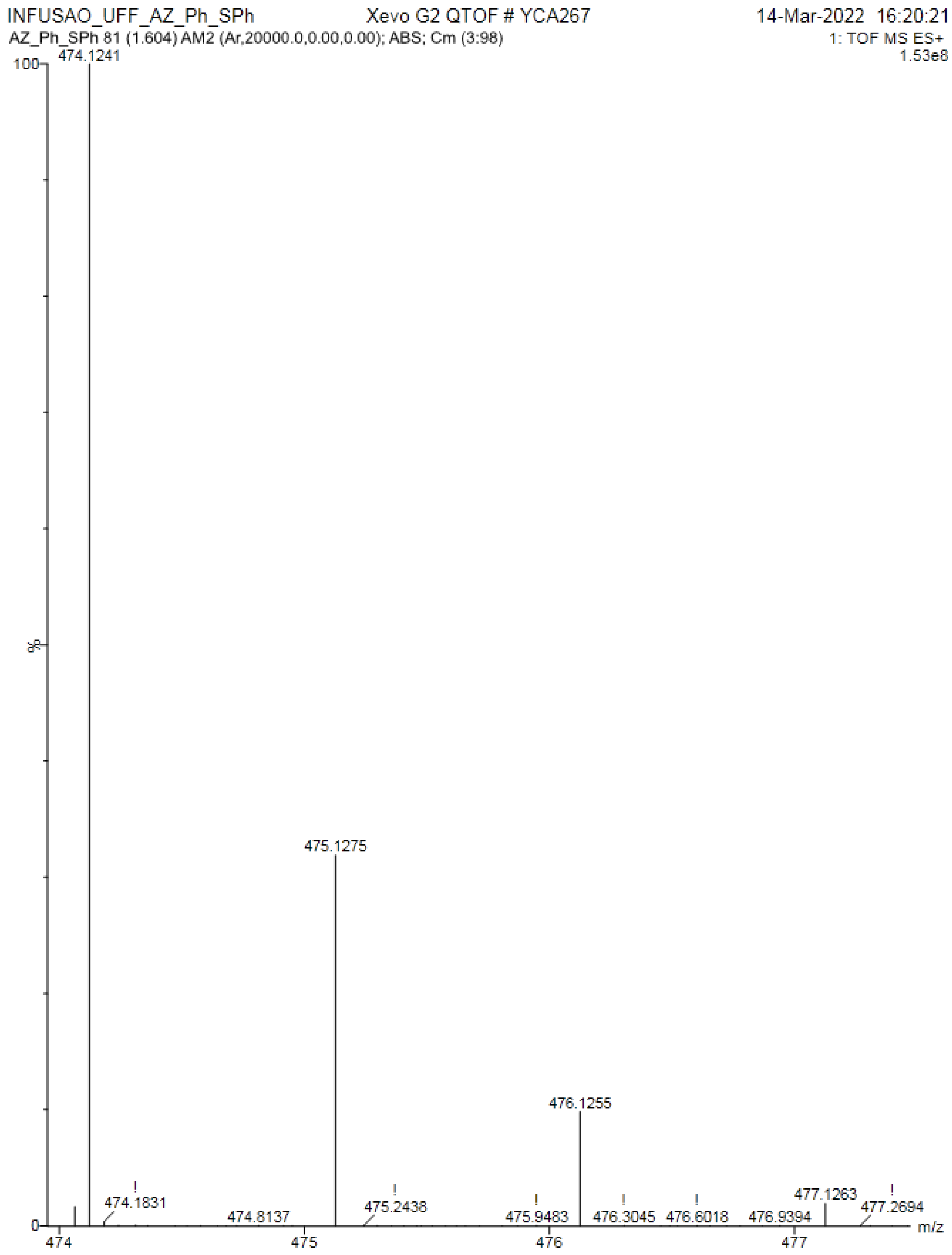

Figure S55. ESI MS spectrum of **10g**.

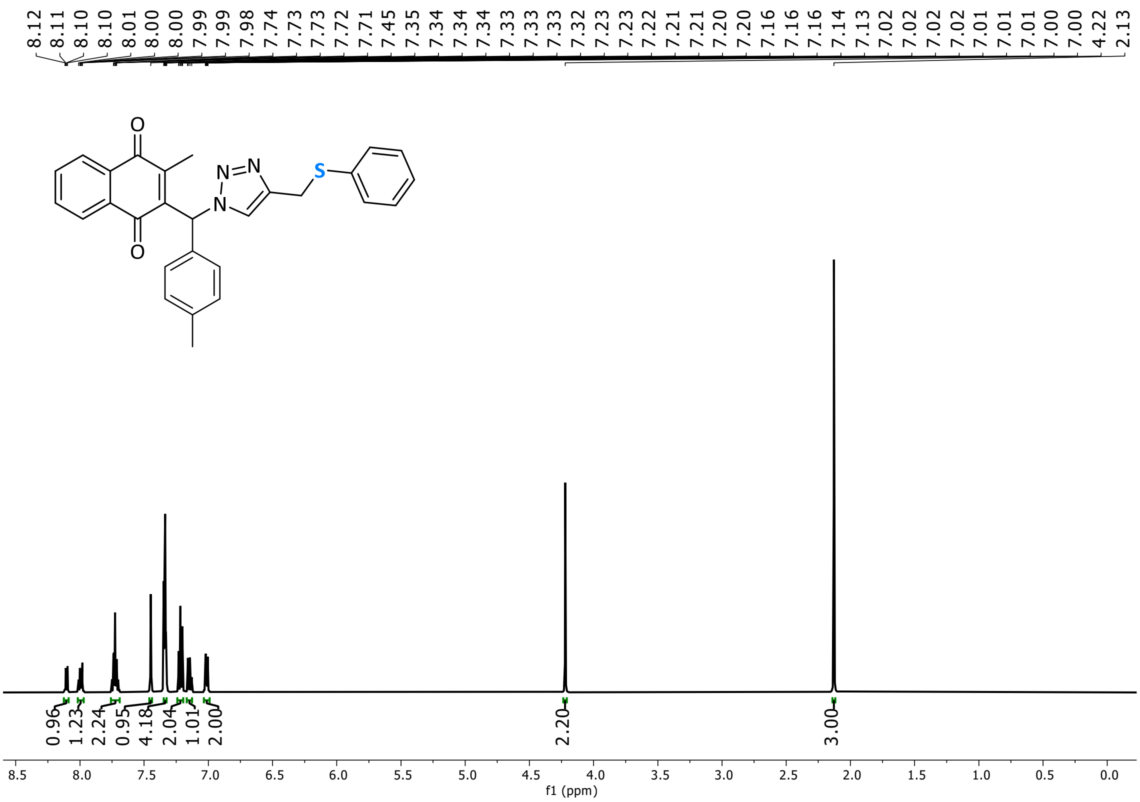

Figure S56. ^{1}H NMR spectrum of compound **10h** in $CDCl_3$ at 500MHz.

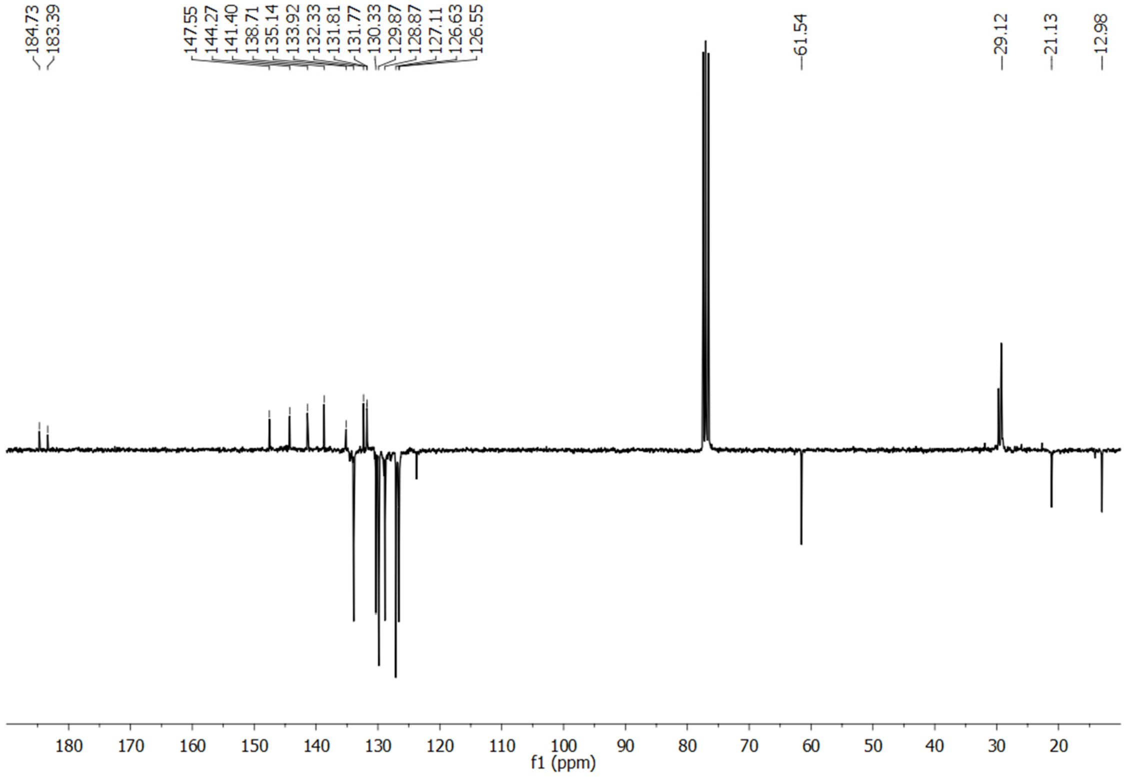

Figure S57. ^{13}C-APT NMR spectrum of compound **10h** in $CDCl_3$ at 75MHz.

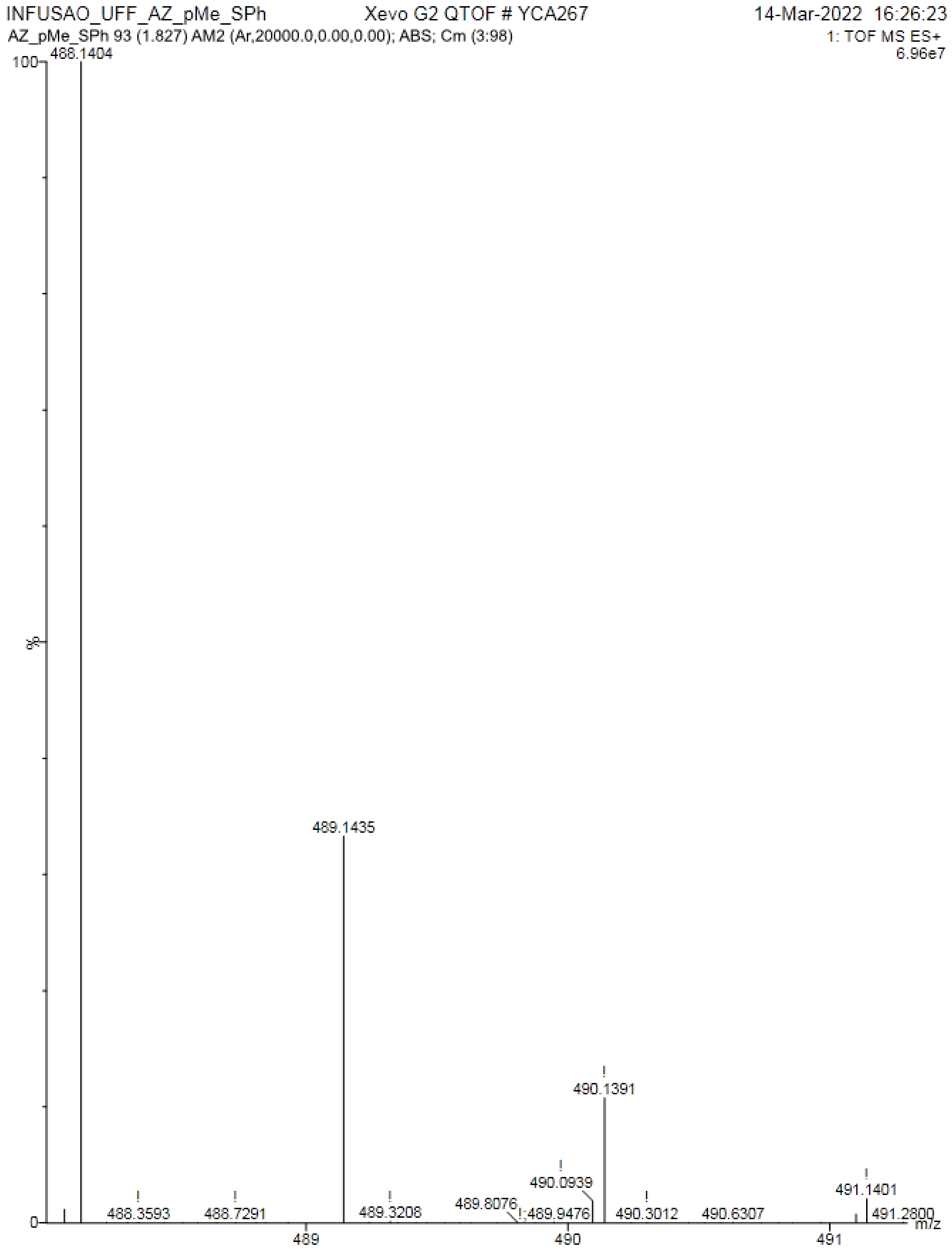

Figure S58. ESI MS spectrum of **10h**.

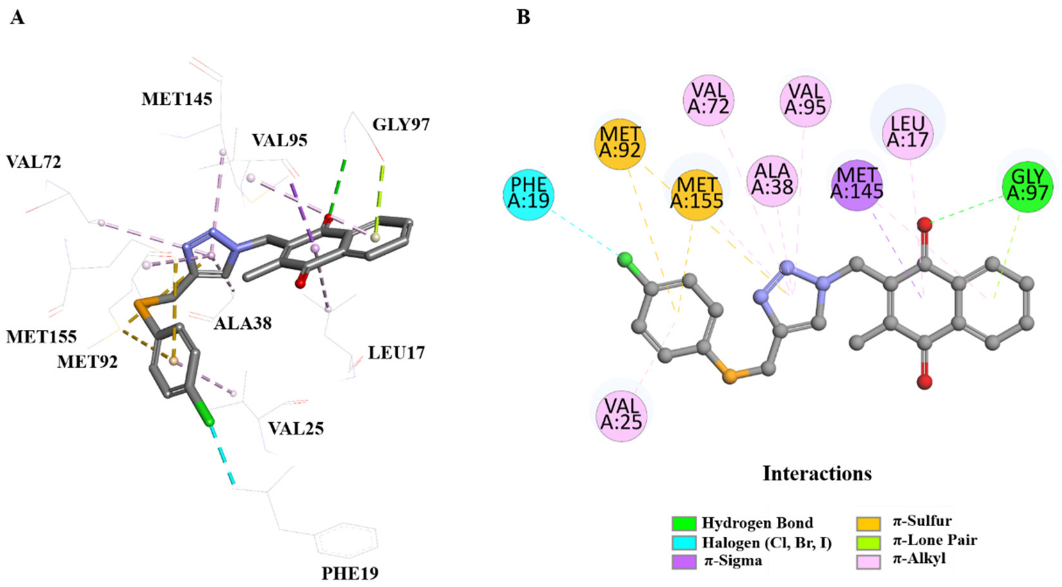

Figure S59. A: 3D view of ligand-receptor interactions between PknB and (**9b**). B: 2D diagram of ligand-receptor interactions between PknB and (**9b**). (PDB ID: 2FUM). Image generated by BIOVIA Discovery Studio Visualizer v24.1.0.23298 (Dassault Systèmes, 2023).
